# Nectar Chemistry or Flower Morphology—What Is More Important for the Reproductive Success of Generalist Orchid *Epipactis palustris* in Natural and Anthropogenic Populations?

**DOI:** 10.3390/ijms222212164

**Published:** 2021-11-10

**Authors:** Emilia Brzosko, Andrzej Bajguz, Justyna Burzyńska, Magdalena Chmur

**Affiliations:** Faculty of Biology, University of Bialystok, Ciolkowskiego 1J, 15-245 Bialystok, Poland; j.burzynska@uwb.edu.pl (J.B.); m.chmur@uwb.edu.pl (M.C.)

**Keywords:** floral display, fruiting, marsh helleborine, nectar amino acids, nectar sugars, pollinaria removal

## Abstract

The aim of this study was to determine the level of reproductive success (RS) in natural and anthropogenic populations of generalist orchid *Epipactis palustris* and its dependence on flower structure and nectar composition, i.e., amino acids and sugars. We found that both pollinaria removal and female reproductive success were high and similar in all populations, despite differences in flower traits and nectar chemistry. Flower structures were weakly correlated with parameters of RS. Nectar traits were more important in shaping RS; although, we noted differentiated selection on nectar components in distinct populations. Individuals in natural populations produced nectar with a larger amount of sugars and amino acids. The sucrose to (fructose and glucose) ratio in natural populations was close to 1, while in anthropogenic ones, a clear domination of fructose and glucose was noted. Our results indicate that the flower traits and nectar composition of *E. palustris* reflect its generalist character and meet the requirements of a wide range of pollinators, differing according to body sizes, mouth apparatus, and dietary needs. Simultaneously, differentiation of nectar chemistry suggests a variation of pollinator assemblages in particular populations or domination of their some groups. To our knowledge, a comparison of nectar chemistry between natural and anthropogenic populations of orchids is reported for the first time in this paper.

## 1. Introduction

To achieve the highest possible reproductive success, plants have evolved different strategies. In animal pollinated plants, the strategies are directed at relations with pollinators. The masters in building the most specialized interaction with their pollinating partners are representatives of Orchidaceae. The majority of them are specialists connected to only one pollinator species (67% of all orchids) or a single functional group [1,2,3,4]. On the opposite point of the continuum of the specialization–generalization scale are generalists, pollinated by a wide range of animals from different systematic and ecological groups. An example of the last group is the object of the present study of *Epipactis palustris*, which is pollinated by more than 100 species [5,6].

To attract pollinators, orchids adapted their flowers structurally and chemically. Many of them (30–40% species) have developed deceptive tactics (mainly food or sexual deception) [7,8,9,10,11]. The important part of Orchidaceae constitutes rewarding species, which reward pollinators through different attractants, such as nectar, fragrances, oils, resin, and wax [12]. The first of them is the most effective for pollination success in orchids [13]. Although the role of the presence of nectar for the reproductive success (RS) of orchids is unquestionable [9,11,13,14], its quantity and quality for pollination effectiveness are documented only for some species [15,16,17,18,19]. Most studies on nectar in orchids, although valuable, only reported about the presence of sugars without ratios between them, or even did not distinguish between the sugars in floral and extrafloral nectar [20,21]. Nevertheless, studies on other plants well document the great variation of nectar properties in different species, distinct populations of a given species, dependence on habitat, flower position on inflorescence, flower age, and other factors. One of the most important findings, due to an evolutionary point of view, is that nectar produced by a given plant species meets the requirements of their pollinators. Relationships between nectar properties and pollinator types confirm many studies [22,23,24,25,26]. Pollinators’ requirements of nectar properties are connected with their body size and behavior (energetic needs), the possibility for them to acquire nectar (mouth apparatus), and gustatory (taste caused by some amino acids (AAs)) [26,27,28,29,30,31]. Preferences of pollinators concern both nectar concentration, sugar proportion, and amino acids composition. For example, bats and hawkmoths feed on the nectar of lower concentrations of sugars, while bees prefer a higher concentration [22,32,33]. The concentration of sugars in orchid nectar sits within a wide range, from a low percentage to 90% [34]. Different pollinators also show distinct preferences to the ratio of main sugars, i.e., saccharose, glucose, and fructose. The extreme example of pollinators preferences to sugar components are some nectarivorous birds and ants, which prefer sucrose-free nectar due to their physiological constrains—the lack of invertase prevents them from attaining sucrose assimilation [35]. Pollinators also select nectar depending on its AA composition. Butterflies choose nectar with high AA concentration, while birds or flies prefer those with lower concentration [28]. Moreover, in the nectar of different species, distinct compositions of AAs were noted—with the domination of some AAs combined with lower concentrations or even absence of others [24,36,37]. The use of nectar by pollinators depends not only on its composition but also on its availability. In orchids, nectar is accumulated in shallow, cup-like structures, at the base of the labellum, in long spurs, in the base of the flower alongside the ovary, and on the side-lobes or along the central groove of the labellum [38]. Nectar located inside the corolla or in the spur is available for specific, restricted groups of pollinators with longer mouth apparatus, while exposed nectar is available for a wide range of pollinators, differing with respect to body sizes and dietary requirements. Exposed nectar is more vulnerable for evaporation and robbery than nectar located in deeper parts of flowers [24,37,39]. Moreover, nectar accumulated in open nectaries is often dominant in hexoses (e.g., fructose and glucose), while in concealed nectaries is most often sucrose dominant [21,24,36,40].

Flower structure plays an important role in shaping relationships between plants and their pollinators; therefore, structure shows adaptation to pollinating animals. The mutual match between pollinator and flower traits is the result of phenotypic selection [41,42]. Despite the fact that the general architecture of orchid flowers has the same scheme, details are differentiated in particular representatives of the family [12] that is strictly connected with the pollinator’s properties. One of the best examples of a match between flowers and pollinators are spurred orchids, for which pollinator-mediated selection on flower traits—especially nectar spur length and corolla tube width—are well documented [41,42,43,44,45,46,47,48,49,50]. Studies on such species show that increasing the mechanical fit between flower and pollinator increases the precision of pollen transfer, thus affecting plant fitness [51]. Pollinator-mediated selection on flower traits is also documented by studies on deceptive orchids [52,53,54,55,56].

The application of orchids’ distinct reproductive strategies translates into their level of reproductive success (RS). For example, rewarded species achieve higher RS than deceptive ones, and among rewarding species, those which produce nectar have the best effectiveness of pollination [7,11,13]. Many data document that reproductive success in orchids is strictly related to an important component of reproductive strategies—the flower’s properties [42,46,49,56]. Generally, orchids are known as a group with a relatively low fruit set, especially non-autogamous species, mainly due to their limited pollinators [11,13,57]. Pollinator deficiency is often noted in anthropogenic populations [58]. Under such circumstances, increasing competition for pollinators may cause intensification of selection on floral traits by increasing pollen limitation [51,59,60,61]. Anthropogenic habitats also offer distinct soil resources, which can shape plant traits such as their size, flower production, or nectar quantity and quality. The dependence of these traits on soil parameters is well documented in orchids [16,17,18,19]. Differentiation of the above-mentioned factors causes spatial and temporary variation of reproductive success [11,13,57,62,63,64]. Some orchids, including *E. palustris*, may colonize different types of secondary habitats [65,66,67,68]. This presents the opportunity for maintaining orchids’ diversity, since they are one of the plant groups that are the most sensitive to habitat loss and destruction due to human activity; therefore, they belong to the most endangered plant groups [69,70] with different threat levels of particular species [71]. The extinction risk of all known orchid species is c.a. 47% [72], and, as an example, 25% of globally extinct orchids are Australian [73]. Kull and Hutchings [74] compared changes in orchids distribution in the United Kingdom and Estonia and found that the mean decline in distribution range for 49 species in the United Kingdom was 50% and the mean decline for 33 orchid species in Estonia was 25%. Similar trends were observed by Jacquemyn, et al. [75] in Flanders and the Netherlands, where during 70 and 50 years, respectively, 81% species decreased distribution range in Flanders, and 78% species decreased distribution area in the Netherlands. Moreover, few species in each area went extinct. Reduction of distribution area and population number in Europe is noted for the object of our studies, *E. palustris* [67]. The threat to orchids is strengthened by the global decline of many plant pollinators, including those crucial for the pollination of orchids [58,76]. For example, the 25% loss of honey bee colonies in Central Europe between 1985 and 2005 has been observed [58].

The main aim of our study was to evaluate the level of pollinaria removal and fruiting and to determine the role of flower structure and nectar composition in shaping RS in natural and anthropogenic *E. palustris* populations. We supposed that RS should be high, due to the following traits of this orchid: (a) as a generalist, it is pollinated by a wide range of pollinators; (b) self-compatible properties enable autogamous and geitonogamous pollination; (c) the presence of nectar enhances the probability of pollination. We also hypothesized the differentiation of nectar characteristics and flower properties between populations, especially between natural and anthropogenic ones.

The answer to the question “what is more important for the reproductive success of generalist orchid *E. palustris* in natural and anthropogenic populations—nectar components or flower morphology?” can help elucidate the evolutionary pathways of different floral traits. Moreover, although the importance of nectar for RS is unquestionable, only a few studies document the role of nectar composition for RS in orchids. Additionally, to our knowledge, this is the first paper where a comparison of nectar chemistry between natural and anthropogenic populations of orchids has been reported.

## 2. Results

### 2.1. Floral Display and Flower Structure

*E. palustris* populations differ significantly in all floral display parameters, i.e., shoot height, inflorescence length, and flower number (Table 1 and Table S1, Figure S1). The highest shoots were observed in the natural ZAB population (62.6 ± 16.1 cm) and the lowest in the anthropogenic SIL population (42.46 ± 7.4 cm). Inflorescence length and number of flowers per shoot was the highest in the anthropogenic SOP population. In ZAB and SIL populations, all floral display traits were monotonically correlated to each other (r_s_ = [0.38, 0.74]; i.e., r_s_ = 0.74 for length of inflorescence vs. shoot height, r_s_ = 0.58 for length of inflorescence vs. shoot height, respectively), while in SOP, the length of inflorescence and number of flowers depended on shoot height (r_s_ = 0.65 and r_s_ = 0.41, respectively). In ROS, statistically significant correlation was found between inflorescence length and number of flowers (r_s_ = 0.53) (Table S2).

All measured flower traits differed between populations (Table 1, Figure S1). The smallest flowers (both their length and width) were noted in natural ROS populations (length of flowers (FH): 10.34 ± 0.56 mm; width of flowers (FW): 20.75 ± 1.34 mm), while the largest in the SOP population (FH: 11.9 ± 1.2 mm and FW: 24.3 ± 2.0 mm). Values of other traits most often shaped according to the same pattern as FH and FW. It should be highlighted that the isthmus area (AI), on which surface nectar is secreted, was larger in anthropogenic (especially in SOP) populations than in natural populations. Spearman’s correlation analysis revealed that almost all flower traits in SOP correlated positively with each other strongly or very strongly (Table S2). In the remaining 3 populations, FH was always correlated with LDS and LP (r_s_ = [0.72, 0.96]), and AI with LI and WI (r_s_ = [0.71, 0.85]).

**Figure 1:**
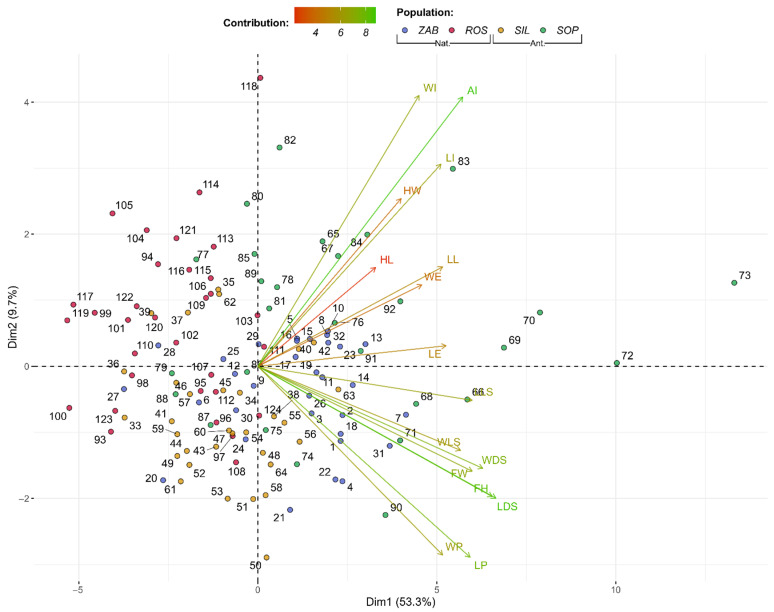
Biplot of flower structure profiles for *Epipactis palustris* natural (Nat.) and anthropogenic (Ant.) populations, showing the first two dimensions or factors (Dim1-2) of PCA that, together, explain 63% of the variance. Biplot vectors indicate the strength and direction of factor loading for the first two factors. Individuals (populations) are color-coded by population.

Furthermore, the flower structure dataset was subjected to principal component analysis (PCA) and its preliminary tests. The *p*-value from Bartlett’s test of sphericity was approximately equal to 0, while the calculated overall measure of sampling adequacy (MSA) from the Kaiser–Meyer–Olkin test was equal to 0.84. MSA for individual parameters ranged from 0.48 (for width of isthmus (WI)) to 0.96 for the width of flowers (FW) (Table S3). Thus, according to Kaiser [77], the MSA value is high enough to perform PCA. According to Cattell’s rule, one or two components should be selected (Figure S2) [78], while Kaiser’s rule indicates that three components should be retained [79]. On the basis of the first axis (Dim1), which accounts for 53.3% of the variation, a separation of all four populations is visible—the following pattern: SOP > ZAB > SIL > ROS usually occurs for all floral structure parameters. SOP and ZAB are mostly associated with positive values of Dim1 (thus higher than average values of floral parameters), while SIL and ROS– are mostly associated with negative values of Dim1. Thus, a sign-based distinction between natural and anthropogenic populations is not possible (Figure 1 and Figure S3).

### 2.2. Nectar Chemistry

#### 2.2.1. Sugars

**Figure 2:**
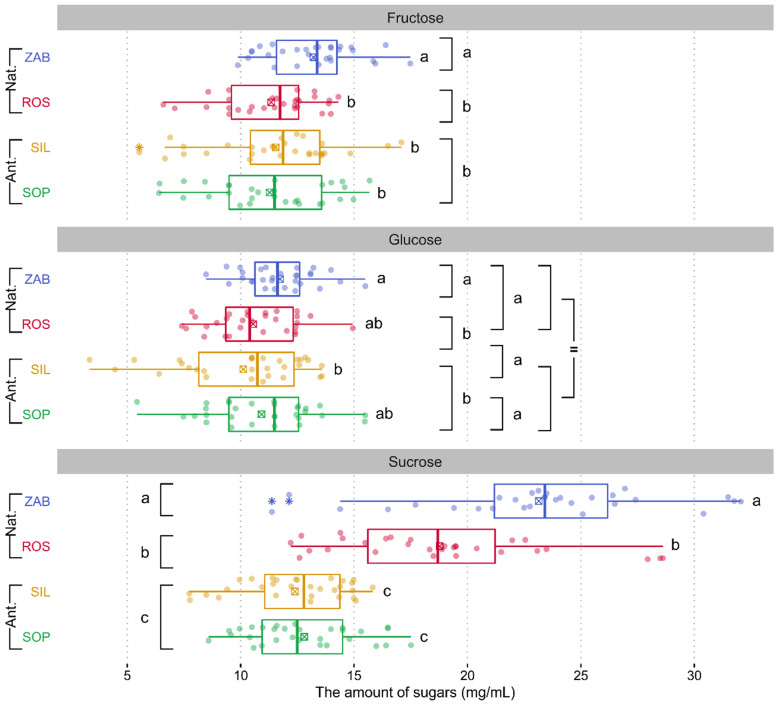
Boxplots of sugar amounts for *Epipactis palustris* natural (Nat.) and anthropogenic (Ant.) populations. Colored dots are individual samples. The crossed square shows the mean. The lower and upper hinges correspond to the lower (Q_1_) and upper (Q_3_) quartiles. Thus, box length shows the interquartile range (IQR). The thicker lines inside the boxes corresponds to the median. The lower whisker extends from the hinge to the smallest value at most Q_1_ − 1.5 × IQR of the hinge. The upper whisker extends from the hinge to the largest value no further than Q_3_ + 1.5 × IQR. Data beyond the end of the whiskers, indicated with an asterisk symbol, are outliers. Different lowercase letters indicate statistically significant differences according to Tukey’s post-hoc test (*p* < 0.05). Symbol “=” means they did not differ significantly. Additional comparisons on the left or right side were shown only when the Nat. or Ant. (or both) populations did not differ significantly.

Our analyses document very low *E. palustris* nectar amounts of three common sugars, i.e., sucrose, fructose, and glucose. We found statistically significant differences between populations in sugars quantity (sum of sugars), excluding glucose content. The total amount of sugars was significantly lower in anthropogenic than in natural populations (SIL: 34.05 mg/mL and SOP: 35.0 mg/mL vs. ZAB: 48.09 mg/mL and ROS: 40.68 mg/mL) (Table S4). Participation of sucrose in nectar was also significantly lower in anthropogenic than in natural populations (Figure 2, Table S5). On the other hand, the sucrose to (fructose and glucose) ratio was more balanced in natural ZAB and ROS populations (0.93 and 0.86, respectively), while in SIL and SOP anthropogenic ones, a clear domination of fructose and glucose was found (0.57 and 0.58, respectively). No statistically significant differences in fructose to glucose ratios were found among populations (Table S4).

#### 2.2.2. Amino Acids

The amount of AAs in *E. palustris* nectar ranged from 0.39 ± 0.002 mg/mL in SOP to 0.52± 0.002 mg/mL in ZAB. Statistically significant differences between populations were noted in the sum of AAs, and the largest differences were observed between natural and anthropogenic populations (Table 2 and Table S6, Figure S4,). In total, 27 distinct AAs were detected in *E. palustris* nectar (20 proteogenic and 7 non-proteogenic) with their different participation in particular populations. Nevertheless, some of them dominated in all populations (glutamic acid (Glu) and glutamine (Gln)—always above 10%). Glu, tyrosine (Tyr), arginine (Arg), and β-alanine (β-Ala) had a significantly higher percentage in natural than in anthropogenic populations. On the other hand, in anthropogenic populations, participation of proline (Pro), alanine (Ala), and phenylalanine (Phe) in nectar was higher than in natural places. It should be noted that β-Ala was observed only in natural populations (ZAB and ROS), citrulline (Cit) only in one natural population (ZAB) (but only in some individuals), and Tau was absent in one anthropogenic population (SOP). It is interesting that anthropogenic populations were characterized by a higher percentage of proteogenic AAs in nectar than natural ones, while non-proteogenic AAs had higher participation in natural populations.

In natural populations, strong monotonic correlations were found, i.e., leucine (Leu) vs. isoleucine (Ile) (r_s_ = {ZAB: 0.75, ROS: 0.74}), ornithine (Orn) vs. glutamine (Gln) (r_s_ = {ZAB: −0.78, ROS: 0.60}), taurine (Tau) vs. Orn (r_s_ = {ZAB: 0.55, ROS: 0.63}), and Tau vs. Gln (r_s_ = {ZAB: −0.60, ROS: 0.68}). Additionally, in ZAB, a correlation between methionine (Met) and lysine (Lys) was noted (r_s_ = −0.64), while in ROS, r_s_ = 0.67 was reported for tryptophan (Trp) vs. threonine (Thr) and Orn vs. glycine (Gly). In anthropogenic populations, no common strong or very strong correlations were reported. However, in the case of SIL, strong monotonic correlations (r_s_ = [0.60, 0.79]) were noted between the following: asparagine (Asn) vs. serine (Ser), histidine (His) vs Asn, Ile vs. alanine (Ala), valine (Val) vs. Leu and Trp, as well as Orn vs His. While, in case of SOP, strong monotonic correlations (r_s_ = [0.60, 0.79]) were noted between the following: Arg vs. Ala, Trp vs. Leu and Met, as well as Orn vs. Lys. It should be also highlighted that, between natural and anthropogenic populations, no intersection of strongly or very strongly correlated AA pairs exist (Table S7).

Different relations between production of sugars and AAs in particular populations was noted. In both natural populations, the sum of AAs positively correlated with the sum of sugars (ZAB: r_s_ = 0.43; *p* < 0.05 and ROS: r_s_ = 0.40; *p* < 0.05), in ZAB with fructose and sucrose amount (r_s_ = 0.44; *p* < 0.05 and r_s_ = 0.38; *p* < 0.05), and in ROS with sucrose amount (r_s_ = 0.44; *p* < 0.05). In anthropogenic populations, positive correlations between AAs amount and percentage of hexoses (i.e., sum of fructose and glucose) were observed (SIL: r_s_ = 0.44; *p* < 0.05 and SOP: r_s_ = 0.38; *p* < 0.05) and negative correlations were observed between AAs amount and percentage of sucrose (SIL: r_s_ = −0.44; *p* < 0.05 and SOP: r_s_ = 0.38; *p* < 0.05).

**Figure 3:**
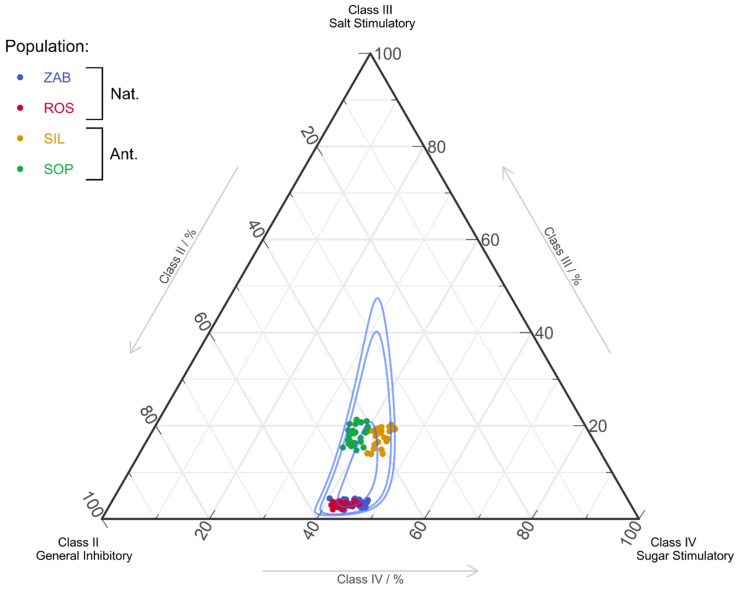
Ternary plot of amino acid classes for *Epipactis palustris* natural (Nat.) and anthropogenic (Ant.) populations: II (Asp, Glu, His, Arg, Lys), III (Pro), and IV (Val, Met, Trp, Phe, Ile, Leu). Blue lines show 50%, 90%, and 95% confidence intervals via the Mahalanobis Distance and use of the Log–Ratio Transformation. The first class of AAs (Asn, Gln, Ala, Cys, Gly, Ser, Thr, Tyr) does not affect the chemoreceptors of fly (data not shown). AAs’ abbreviations and full names are present in Table 2.

We found a notable difference between natural and anthropogenic populations in participation of AA from distinct taste classes (Figure 3). The percentage share of class II AAs was approximately 35–48% for natural populations and 48–56% for anthropogenic populations, while class IV was 36–42% for SOP and 42–48% for SIL. The class II of AAs had higher participation in natural populations. On the other hand, the class III group, represented in *E. palustris* nectar only by Pro, had about five times higher participation in anthropogenic populations than in natural populations (Table 2).

**Figure 4:**
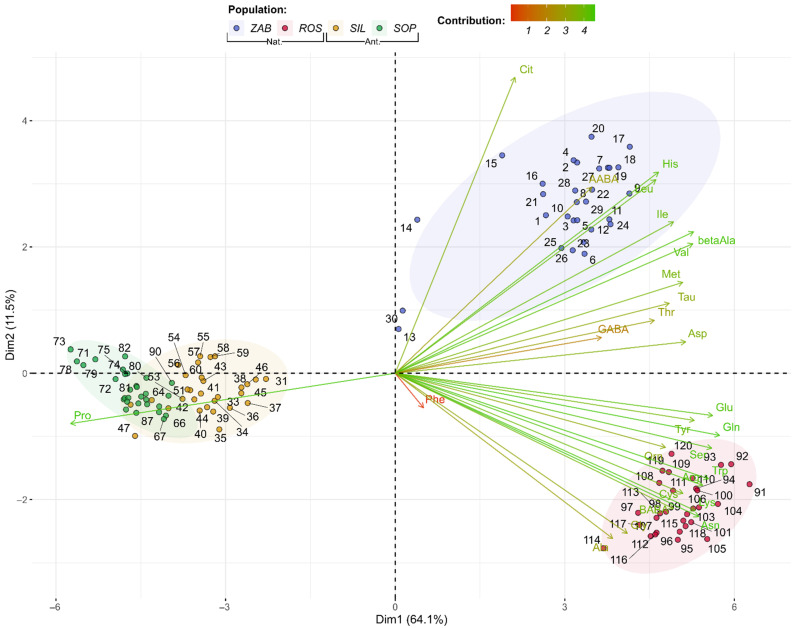
Biplot of amino acid profiles for *Epipactis palustris* natural (Nat.) and anthropogenic (Ant.) populations, showing the first two dimensions or factors (Dim1-2) of PCA that, together, explain 75.52% of the variance. Biplot vectors indicate the strength and direction of factor loading for the first two factors. Individuals (populations) are color-coded by population. Ellipses around the individuals show assumed 95% multivariate normal distribution.

Moreover, AAs were subjected to principal component analysis (PCA) and its preliminary tests. The *p*-value from Bartlett’s test of sphericity was approximately equal to 0, while the calculated overall measure of sampling adequacy (MSA) from the Kaiser–Meyer–Olkin test was equal to 0.95 (Table S8). MSA for individual AAs ranged from 0.44 (Phe was the only AA with almost no interpopulation differences and very high data deviation, Figure S2) to 0.98 for Tyr, Trp, and Val (Table S8). Thus, according to Kaiser [77], the MSA value is high enough to perform PCA. According to Cattell’s rule, one or two components should be selected (Figure S5) [78], while Kaiser’s rule indicated that three components should be retained [79]. Finally, the first two components that explain about 75.5% of the variance were preserved. PCA grouped together anthropogenic populations (SIL and SOP), as they had much higher average Pro level and lower levels of other AAs (Figure 4). Differences between ZAB and ROS populations are also visible, e.g., much higher average Cit, His, β-Ala, and Ile levels, as well as lower Arg, Asn, Trp, β-aminobutyric acid (BABA), and Lys. On the basis of the first axis (Dim1), which accounts for 64.1% of the variation, there is a clear separation of the natural vs. anthropogenic populations, particularly due to differences in Pro. Based on the second axis (Dim2, 11.5%), the two natural (Nat.) populations are also separated, while the anthropogenic (Ant.) are not (Figure 4 and Figure S6).

### 2.3. Reproductive Success

Reproductive success in *E. palustris* populations was high (Table 3). Female reproductive success (FRS—the proportion of developed fruits to the number of flowers on the inflorescence) shaped from 81.47 ± 4.19% in ROS to 90.60 ± 2.49% in SOP (Table 3) and did not differ between populations (F = 0.862; *p* = 0.46). Pollinaria removal (PR) was also similar in all populations (F = 1.289; *p* = 0.28); although, activity of insects was about 10% higher in SOP (96.55 ± 1.61%) than in other populations (Table 3). The efficiency of pollination was high—PR to FRS ratio was equal to about 1 in all populations. Although average values of indexes of reproductive success are similar in the populations studied, we observed some differences in details of the pollination process at an individual level. In ROS, SIL, and SOP populations, about one third of individuals (11, 9, and 9, respectively) had higher PR than FRS, while in ZAB only five of them had PR larger than FRS. On the other hand, we noted higher FRS than PR in ROS and SIL (7 and 9 individuals, respectively). In ZAB, only two shoots had higher fruiting than PR, while in SOP, we did not observe such cases.

## 3. Discussion

In line with our expectations and the results of earlier studies [5,67,80,81], high levels of PR and FRS (above 80%) were found in both natural and anthropogenic *E. palustris* populations. Jacquemyn, et al. [67], on the basis of Claessens and Kleynen [5] data from 24 populations of this species, reported that the average fruit set shaped at 77.6% %, and Jacquemyn and Brys [82] noted 70% fruiting in Belgian populations. We also found the high RS and pollination efficiency (in all places PR to FRS ratio equaled about 1). This result contrasts with the founding of Jacquemyn and Brys [82], who noted that fruiting in *E. palustris* populations was higher than the level of pollinaria removal.

Although pollinator deficiency is considered the main factor restricting RS in orchids [11,13,57], a high level of RS and pollination efficiency in our studies suggest that pollinators of *E. palustris* are abundant in all populations. Nevertheless, assemblages of insects and the dominant pollinators may differ from one part of geographical range to another [5,6,83]. High number of pollinators of this orchid (142 species [5]) and a wide range of their sizes and requirements increase the probability of pollination. Through a diversity of potential pollinators, we can also explain the lack of differences in RS between natural and anthropogenic populations. Our results indicate that in each of them, pollinators assemblages are large and diverse enough to ensure RS at the observed level. This result contrasts with the results of other studies, where fruiting was lower in anthropogenic than in natural populations. Exceptionally low levels of fruiting were observed by Jermakowicz and Brzosko [59] in anthropogenic populations of *Malaxis monophyllos*. On the other hand, Pellegrino and Bellusci [84] noted an almost seven times lower fruit set in anthropogenic than in natural populations of *Serapias cordigera* in Italy. In a population of *Oncidium ascendens* from rainforest from Mexico, fruit production was almost two times higher than in populations from synanthropic habitats [85]. The authors of these studies recognized that pollinator deficiency in altered habitats was the main factor, which decreased RS in these species. In our opinion, differences between species characters of orchids studied by Pellegrino and Bellusci [84] and Parra-Tabla, et al. [85] and *E. palustris* could also cause distinct answers for habitat types. *S. cordigera* is deceptive species and relies on relatively restricted groups of pollinators in comparison with *E. palustris*; additionally, *O. ascendens* is a self-incompatible species, whose sexual reproduction depends on cross-pollination by the native bee *Trigona nigra*. It could be suggested that the properties of *E. palustris* (its generalist character, presence of nectar, and spontaneous autogamy) and pollinators behavior (penetration of many flowers on inflorescence) are advantages, which ensure effective pollination regardless of habitat. Anthropogenic habitats are generally recognized as those with poorer assemblages of pollinators, which negatively influences plant RS, but some of them seem to be suitable for plant–pollinator interactions. For example, Rewicz, et al. [86] reported higher RS in anthropogenic than in natural populations of *E. helleborine*, due to the larger diversity of pollinators in the first type of habitat. Although two *E. palustris* populations exist within the city border, in changed places, within populations’ area, and in neighboring communities, other flowering species grew, which can attract many insects, including *E. palustris* pollinators. Moreover, allotments are placed in the vicinity of SIL population, which may increase pollinator numbers.

In the light of our results, it seems that generalists are less sensitive for pollinator deficiency, even in anthropogenic habitats. This is in accordance with findings of other authors, who state that a decrease of fruit set as a result of the reduction in insect movements is particularly strong for specialists that show a high degree of dependence on their pollinators for fruit production [11,84,87]. Higher specialization levels and anthropogenic declines in pollinator populations can also intensify selection on floral traits [51]. The generalist character of *E. palustris* and pollinator efficiency could explain the very weak selection on flower and floral display traits. Only ten correlations between them and parameters of reproductive success were observed (among 152 tested cases), and only four of these concerned flower structures. Five statistically significant correlations were found in the anthropogenic SOP population and all of them were negative. In this population, lower individuals with shorter inflorescences and a lower number of flowers were favored. This can reflect the behavior of pollinators in this locality. First, they may operate at the lower part of vegetation, and secondly, penetrating a given inflorescence, they are able (or need) to acquire nectar from a restricted number of flowers. On the other hand, in ZAB, fruiting was higher on longer inflorescences. In this place, *E. palustris* grows in tall sedges, and probably shoots should be higher than neighboring plants to be recognized by pollinators. Stronger pollinator-mediated selection on inflorescence was noted in taller than in shorter vegetation [46,88,89]. The case of ZAB population is also in accordance with the common expectation that more fruits often develop on larger inflorescences because they attract more pollinators, which visit more flowers on larger inflorescences [48,90,91,92]. On the other hand, the SOP population may illustrate situations, that smaller inflorescences are favored by natural selection when larger inflorescences suffer factors decreasing fitness, such as the higher probability of geitonogamy or intense herbivore activity [92,93,94].

One of the most important evolutionary mechanisms, crucial for successful pollination, is the mechanical fit between plants and their pollinators [2,7,8,41,95,96]. Such a match is generally stronger in specialized systems [51,97], which confirms, for example, the results of studies on long-spurred orchids [41,45,47,48]. These findings confirm the results of our studies because only four distinct flower traits influenced RS in three among four populations. These traits seem unimportant for pollinators, which may suggest that observed correlations are random or their functions are difficult to explain. The lack of strong selection pressures on these traits maintains variation in flower traits [98].

Nevertheless, it should be noted that the isthmus area was significantly larger in the anthropogenic population than in the natural population, suggesting that pollinators with distinct mouth apparatus operate in two population groups. The important point, which enables understanding the evolution of plant–pollinator interactions, is knowledge about the importance of floral rewards, including nectar, for RS [99,100]. Nectar properties shape the growth, survival, reproduction, and behavior of nectar-feeding animals [30,31,35,101,102]. Our results suggest stronger dependence of RS in *E. palustris* populations on nectar properties than flower structure. Nectar characteristics influenced mainly PR (16 statistically significant correlations observed among 21). This is in contrast to our study on another generalist orchid, *Neottia ovata*, where nectar properties shaped mainly FRS [19]. Similarly to Percival [21], we found three main sugars in *E. palustris* nectar (sucrose, fructose, and glucose) with their amount shown to be larger in natural than anthropogenic populations. Sugar components influenced RS only in the natural ROS population, where PR was positively influenced by hexose (i.e., fructose and glucose) amounts and FRS was positively influenced by fructose amount. This is interesting because in both natural populations, sucrose percentage is significantly higher than in anthropogenic ones. Positive selection on hexoses in ROS may suggest that in this population insects, which prefer nectar rich in monosaccharides are important pollinators, and the amount of hexoses in this population is not enough to provide for their needs. Similar insects could be abundant in anthropogenic populations, where hexoses were more abundant than in natural populations. Preferences for hexoses, taken up more easily than sucrose, show nonspecialized insects, i.e., syrphids, flies, and beetles [25]. Insects from these groups were noted as *E. palustris* pollinators [6,67,83,99]. Nonspecialized insects choose hexose-rich nectar (especially fructose) because it is easier absorbed due to lower viscosity [32]. Moreover, some ants (often observed by us on *E. palustris* shoots and noted by Jakubska-Busse and Kadej [103]) even prefer sucrose-free nectar because they are not able to assimilate this sugar due to lack of invertase [29]. The lack of selection on nectar sugars in three populations may suggest that these nectar components are not aimed at any of the pollinator group and sugar composition met the requirements of pollinated insects. Similar results were obtained for another generalist orchid, *N. ovata* [19]. Different sucrose to (fructose and glucose) ratios (~1 in natural and ~0.5 in anthropogenic populations) suggest distinct pollinator assemblages in these two population groups. Larger sucrose content in natural populations could indicate that such insects as honey bees and bumblebees, which prefer this sugar, are main pollinators in these places. These insects were recognized as main pollinators in some Polish *E. palustris* populations by Jakubska-Busse and Kadej [103]. It should be noted that different sugar ratios in natural and anthropogenic *E. palustris* populations only partially confirms the statement that nectar secreted in open flowers is dominated by glucose and fructose [36,40].

Nectar in particular populations also differed according to AAs composition. Their total amount was higher in natural populations, but proteogenic AAs have larger participation in anthropogenic ones. At the species level, we noted a high number of different AAs (27, including 20 proteogenic and 7 non-proteogenic), similarly to another generalist orchid *N. ovata* (28 AAs; [19]). Pais, et al. [40] found only 17 AAs in *E. atropurpurea*. Fewer AAs than those in our study were observed in the nectar of specialist spurred orchids [15,17]. Additionally, we found domination of different AAs in the two population groups—Glu, Tyr, Arg, and β-Ala were more abundant in natural populations and Pro, Ala, and Phe were more abundant in anthropogenic populations. The most common AAs in *E. palustris* nectar are Gln, Glu, and serine (Ser), which are always above 10%. These AAs were also found to be among the most abundant in the nectar of generalist *N. ovata* [19]. Gln and Glu are needed for energetically exhaustive flights, while Glu and Ser influence pollinator behavior [17,18,28].

Available studies document preferences of pollinators both to total AAs amount in nectar and to particular AAs. Although the importance of AAs for pollinator life is poorly studied, the role some of them are known. First of all, they play a nutritional function and attract or discourage pollinators. One of the most common AAs in plants [24,104]—important for many pollinators, especially Hymenoptera—is Pro, production of which is more expensive than other nectar components [105]. Its participation constitutes the greatest difference between natural and anthropogenic populations among measured nectar components. In anthropogenic populations, it was one of the three most abundant AAs, and its amount was about five times higher than in natural populations. Like the majority of amino acids, proline can be used in energy production [104]. This AA rewards pollinators, propels the lift phase of the flight [105,106], and stimulates insects’ salt receptors, which initiate feeding [26,35,107]. Carter, et al. [105] found that Pro accumulation is a plants’ answer to stress factors. Through the last function could be explained a few times, larger amounts of this AA in were found anthropogenic populations than in natural population, since changed habitats are stressful for plants. It can indicate that Pro plays an important role in the metabolism of *E. palustris* pollinators in anthropogenic populations. Bertazzini, et al. [108] documented a preference of honey bees for proline-enriched artificial nectar. The other AA, whose amount was larger in the anthropogenic than in the natural population, is Phe. It has a strong phagostimulatory effect on bees and its concentration is highly variable [102,109]. Petanidou, et al. [102] attributed the dominance of Phe in the Mediterranean to the high number of bees, especially long-tongued bees. The authors suggest that in the Mediterranean, such bees act as crucial selective factors for Phe-rich nectars. The last abundant AA in anthropogenic populations is Ala, which influences insect growth [17]. Aspartic acid (Asp), like Glu and Ser, influences pollinator behavior [17,28] and disgusts pollinators [102]. The second importance of Asp may explain its negative influence on PR in ROS. In the same way, a negative correlation between the percentage of Ser and FRS in SIL population could be explained. A negative response of honey bees to Ser was reported [108]. On the other hand, Kim and Smith [110] showed that Gly elicited a feeding response in honeybees.

Variation in amount and participation of nectar components, and the differentiation of selection on distinct constituents in particular populations suggest, again, that different pollinators with different nutritional needs operate in distinct populations. This supposition could be strengthened by the results showing preferences of pollinators to different taste classes. The most sensitive for nectar taste were pollinators in ROS, where we found positive correlations between PR and percentage of AAs from taste class IV (stimulation the sugar receptor cell), while negative between PR and taste class II (inhibition of the three types of chemosensory cell: salt, sugar, and water). Among the remaining three populations, only in SIL AAs from taste class I positively shaped PR. This confirms the results of earlier studies [28,111], in which it was recognized that nectar amino acids might be detectable by pollinators and may contribute to the overall taste of nectar. We noted the importance of nectar taste for pollinators, and consequently for RS, in other orchids, both in generalist (with open nectaries) and in specialist (with the accumulation of nectar in the spur) [15,19].

The nectar composition can be modified by habitat properties, especially soil nutrients [18,104]. These authors documented changes in the total concentration of amino acids, as well as changes in the amount of some of them after fertilization. Moreover, they stated that such changes may have implications for plant–insect interactions, as local populations of pollinators may benefit from the increased amino acid content of the nectar and preferentially visit plants growing in high nutrient conditions. The influence of soil parameters, especially carbon and the carbon to nitrogen ratio, influenced flower structure and nectar chemistry in generalist *N. ovata* [19]. We did not analyze soil chemistry because differences between natural and anthropogenic habitats are so evident (see Section 4.2), which, with high probability, may be recognized as one of the causes of differentiation of nectar traits between them. Nevertheless, soil in natural populations seems to be richer in elements required to produce more sugars and AAs.

Results of our studies confirm the generalist character of *E. palustris*. High levels of RS in all populations indicate that both flower traits and nectar chemistry, and variation of these properties meet the needs of wide, differentiated pollinator groups. Simultaneously, the lack of selection on flower traits and stronger selection on nectar components suggest that pollinators are more sensitive to nectar properties, including taste. Moreover, selection on distinct nectar characters in particular populations may indicate that different pollinator assemblages operate within them.

The most important finding of our research is documentation (to our knowledge, it is the first such report) of significant differences in nectar properties between natural and anthropogenic orchid populations. We suggest that they are caused by the differentiation of pollinators in these two habitat types or are stronger depending on soil characters (or both). However, to precisely point out the most important factor, more detailed studies should be conducted. The results of our studies importantly enrich the knowledge needed to explain mechanisms, which underlie plant–pollinator interactions.

## 4. Materials and Methods

### 4.1. Study Species

*E. palustris* is widely distributed throughout most of Europe but is absent in the Southern Mediterranean regions and extreme north [112]. This species usually occupies calcareous, nutrient-poor, and moist–wet substrates, mainly in full sunlight. It exists in wet dune slacks, calcareous fens, and on peat, but may also occur on sandy substrates overlying heavy clay or loamy soils [67].

*E. palustris* is rhizomatous species. Each shoot has about six leaves in the lower part and a few more scattered ones below the inflorescence. The inflorescence produces about twelve flowers (sometimes more than 20). The flowers are usually more or less tinged with a rose, red, or brown coloration [67,81]. A wide spectrum of insects visits *E. palustris* flowers, but the main pollinators are Diptera (i.e., *Empis* sp., and *Episyrphus* sp.) and Hymenoptera (i.e., *Apis mellifera*, *Bombus lapidarius*, and *Bombus lucorum*) [6,83]. Claessens and Kleynen [5] found 142 pollinators of this orchid in the literature. Insects are attracted by strong scent (with eugenol and vanillin, as the crucial components attracting Diptera) and nectar with attractants such as nonanal (pelargonaldehyde), decanal, eicosanol, and its derivatives [83]. The shallow nectary is located on the labellum, and nectar is secreted on the whole surface of lip callus and abaxial side of isthmus in hypochile [113]. E. palustris has the potential for spontaneous autogamy [67,80]. Fruiting takes place August–September, depending on location and weather conditions [81].

### 4.2. Study Area

This study was performed in July and August 2021 in four populations of *E. palustris* in northeast Poland, two of which were localized in well-preserved natural peat bogs. ZAB population is localized in the Biebrza National Park—one of the biggest areas of peatlands in Europe. ROS population is localized in Rospuda valley. It is a vast, moss-free, low, and transitional peat bog, with rich and unique flora (four plant species listed in Annex 2 of the EU Habitats Directive, fifteen plant species listed in *The Polish Red Book of Plants*). The bog is also the only refuge in Poland of a rare orchid species in Europe—*Herminium monorchis*. Natural populations are distanced c.a. 70–100 km from Bialystok, the largest city in NE Poland, and at least a few kilometers from the nearest villages. Two other populations (SIL and SOP) exist in anthropogenic habitats at the border of Bialystok city. The SIL population exists in an abandoned gravel pit (c.a. 3 ha). The SOP population exists within the highly damaged soligenic peat bog (0.5 ha), which is under advanced secondary succession, with a lowered level of groundwater and the presence of alien species. Both anthropogenic populations are surrounded by human-changed habitats, typical for urban areas.

### 4.3. Fieldwork and Floral Trait Measurements

In populations studies, 30–32 flowering individuals were chosen and marked. In the field, the floral display traits, i.e., the height of shoots, length of inflorescence, and the number of flowers, were quantified. The five lowest flowers from each inflorescence were collected and used for the evaluation of nectar composition and measurement of the morphological variables of flowers (full names of abbreviations are present in Table 4). The area of isthmus (AI), as the product of LI and WI, was amounted. The isthmus size was considered as a measure of nectar quantity. Flower traits of one individual are given as average from five measurements.

Samples from all populations were collected 7–12 July during the peak of flowering under sunny and hot weather (the temperature of each day was about 30 °C).

The morphological measures were taken using an opto-digital microscope DSX110 (Olympus Life Science, Waltham, MA, USA) in the Laboratory of Insect Evolutionary Biology and Ecology, Faculty of Biology, University of Bialystok.

To assess the level of reproductive success (RS), the shoots were marked and the number of flowers per inflorescence in full blooming were counted. During the maturation of capsules, FRS and PR were quantified. FRS was evaluated as the proportion of developed fruits to the number of flowers on the inflorescence and was given in percentages. PR was determined in percentages (PR to the total number of pollinaria for each inflorescence). The efficiencies of pollination were also evaluated, found as the ratio of PR to FRS—the higher the index, the lower the pollination efficiency within a population.

### 4.4. Nectar Analysis

#### 4.4.1. Nectar Isolation

Flower nectar isolation was performed using a water washing method [114]. Five flowers per sample were placed into a 2 mL Eppendorf tube, containing 1 mL of distilled water and shaken in a laboratory thermomixer (120 rpm, 21 °C, 45 min; Eppendorf Corporate, Hamburg, Germany) for the nectar efflux. Then, the flowers were removed from the tubes, and the mixture of water with nectar was evaporated to dryness by centrifugal vacuum concentrator (45 °C, Eppendorf Concentrator Plus, Eppendorf Corporate, Hamburg, Germany). The obtained pellet was dissolved in 20 µL of distilled water, then transferred into the centrifuge tube with a filter and centrifuged to remove impurities (9000× *g*, 5 min; MPW-55 Med. Instruments, Gliwice, Poland). The purged extract was collected in a glass vial with a 250 µL insert with polymer feet.

#### 4.4.2. Sugar and Amino Acid Determination

Determination and quantification of sugars and AAs were performed using the high-performance liquid chromatography (HPLC) method. An Agilent 1260 Infinity Series HPLC apparat (Agilent Technologies, Inc., Santa Clara, CA, USA) with quaternary pump with an in-line vacuum degasser, a thermostatted column, and a refrigerated autosampler with an autoinjector sample loop was used.

For sugar analysis, a ZORBAX Carbohydrate Analysis Column (4.6 mm × 250 mm, 5 µm) (Agilent Technologies, Inc., Santa Clara, CA, USA), at a temperature of 30 °C and a refractive index detector, was applied. The mobile phase was a solution of acetonitrile and water (70:30, *v*/*v*) at a flow rate of 1.4 mL/min. The injection volume was 10 µL. The total time of analysis was 15 min [15].

Meanwhile, for AA detection (Table 5), an automatic program of derivatization was set. Thus, the *o*-phthalaldehyde and 9-fluorenylmethyl chloroformate reagents were used for the derivatization of primary and secondary AAs [15]. The Agilent Zorbax Eclipse Plus C_18_ (4.6 × 150 mm, 5 µm) column (Agilent Technologies, Inc., Santa Clara, CA, USA), at a temperature of 40 °C, was used to separate individual AAs. Detection of primary AAs was performed by a photodiode array detector at 388 nm, while detection of secondary AAs was performed by a fluorescence detector with an excitation wavelength of 266 nm and an emission wavelength of 305 nm. The injection volume was 5 µL. The flow rate was 1 mL/min. Eluent A of the mobile phase was 40 mM NaH_2_PO_4_ (pH 7.8, adjusted by 10 M NaOH solution), while eluent B was a mixture including acetonitrile, methanol, and water (45:45:10, *v*/*v*/*v*). The gradient was the following: 0–5 min, 100–90% A; 5–25 min, 90–59.5% A; 25–30 min, 59.5–37% A; 30–35 min, 37–18% A; 35–37 min, 18–0% A; 37–40 min, 0% A; and 40–43 min, 100% A.

The analytical data were integrated using the Agilent OpenLab CDS ChemStation software (Agilent Technologies, Inc., Santa Clara, CA, USA) for liquid chromatography systems. Identification of sugars and AAs was performed by comparing retention times of individual sugars and AAs in the reference vs. test solution. The concentration of these compounds was assayed based on comparisons of peak areas obtained for the samples, investigated with those of the reference solutions.

### 4.5. Statistical Analysis

The R programming language or statistical environment was used to perform all statistical computations and analyses, as well as to prepare graphics and transform data for tabular representation [115,116]. The dataset of sugars was subjected to two-way ANOVA followed by Tukey’s post-hoc test, while AAs, floral display, and flower structure datasets were supplied to either (a) two-way ANOVA followed by Tukey’s post-hoc test or (b) the Kruskal–Wallis test followed by a pairwise Wilcoxon Rank Sum test with Benjamini–Hochberg adjustment, which compared the median values of different parameters between populations, depending on ANOVA pre-conditions (verified using Shapiro–Wilk test and Bartlett’s test) (Table S1, Table S5, Table S6, Figure S1, Figure S4) [116,117,118,119,120]. Furthermore, a set of descriptive statistics (mean, standard error, quartiles, and interquartile range) was calculated for AAs, sugars, floral display, and flower structure. For all tests, the significance level was α = 0.05. In order to check if a monotonic relationship exists between floral display and flower structure parameters, Spearman’s rank correlations were calculated (Table S2) using the ‘rcorr’ function from the ‘Hmisc’ package. Spearman’s correlations were also calculated between AAs (Table S7). Correlations were considered significant for *p* < 0.05.

To analyze the effect of AAs on insect chemoreceptors, all identified and determined AAs were grouped into four classes [24] (full names of abbreviations are present in Table 5): I. Asn, Gln, Ala, Cys, Gly, Ser, Thr, and Tyr (no effect on the chemoreceptors of fly); II. Arg, Asp, Glu, His, and Lys (inhibition of fly chemoreceptors); III. Pro (stimulation of the salt cell); and IV. Ile, Leu, Met, Phe, Trp, and Val (ability to stimulate the sugar cell) and presented as a ternary plot [121]. Principal component analysis (PCA) was used to simplify the exploration of AAs. To build the PCA model, the FactoMineR package was used [122]. Two tests that indicate the suitability of the AA dataset for structure detection and reduction were performed—Bartlett’s test of sphericity [123] and the Kaiser–Meyer–Olkin test of factorial adequacy (psych package [124]). Unit variance scaling of the data was applied; thus, PCA was performed on a correlation matrix, rather than on a covariance matrix. Number of principal components to retain was selected with the help of Cattell’s and Kaiser’s rules [78,79]. All biplots were created using the factoextra package [125]. Moreover a PCA was also applies to flower structure dataset using an approach identical to that used for AAs dataset.

## Figures and Tables

**Table 1:** Variation of floral display and flower structure in *Epipactis palustris* natural (Nat.) and anthropogenic (Ant.) populations. Data (*n* = 30) represent the mean ($\bar{x}$) ± standard error (SE), lower quartile (Q_1_), median (Q_2_), upper quartile (Q_3_), and interquartile range (IQR). Different lowercase letters indicate statistically significant differences according to Tukey’s post-hoc test (*p* < 0.05). Different uppercase letters indicate statistically significant differences according to the pairwise Wilcoxon Rank Sum test with Benjamini–Hochberg adjustment (*p* < 0.05). Additional comparisons were shown only when populations within Nat. or Ant. (or both) do not differ significantly.

Parameter	Statistic/Comparison	Natural (Nat.) Populations		Anthropogenic (Ant.) Populations	
		ZAB	ROS	SIL	SOP
Shoot height (cm)	$\bar{x}\;$± SE	62.59 ± 3.05 ^a^	51.69 ± 1.61 ^b^	42.47 ± 1.36 ^c^	54.62 ± 2.14 ^b^
	Q_1_	51.50	47.01	37.5	43.25
	Q_2_ (IQR)	64.75 (18.75)	53 (10)	42.75 (9)	56.75 (16.75)
	Q_3_	70.25	57.00	46.5.	60.00
Inflorescence length (cm)	$\bar{x}\;$± SE	9.43 ± 0.58 ^b^	10.43 ± 0.53 ^b^	10.26 ± 0.33 ^b^	13.46 ± 0.79 ^a^
	Q_1_	7.25	9.00	9.00	10.62
	Q_2_ (IQR)	9.25 (3.75)	10 (3.00)	10.00 (3.00)	13.50 (5.38)
	Q_3_	11.00	12.00	12.00	16.00
	Nat. vs. SIL vs. SOP	b		b	a
Flower number	$\bar{x}\;$± SE	9.00 ± 0.54	10.66 ± 0.46	9.53 ± 0.43	11.85 ± 0.81
	Q_1_	7.00	9.00	9.00	9.25
	Q_2_ (IQR)	8.00 (4.50) ^B^	11.00 (3.00) ^A^	9.00 (1.75) ^B^	11.00 (5.75) ^A^
	Q_3_	11.50	12.00	10.75	15.00
Length of dorsal sepal (LDS) (mm)	$\bar{x\;}$± SE	11.22 ± 0.13 ^a^	10.01 ± 0.09 ^c^	10.70 ± 0.10 ^b^	11.55 ± 0.18 ^a^
	Q_1_	10.64	9.56	10.33	10.99
	Q_2_ (IQR)	11.37 (1.04)	9.99 (0.81)	10.69 (0.66)	11.34 (1.07)
	Q_3_	11.68	10.37	10.99	12.06
Width of dorsal sepal (WDS) (mm)	$\bar{x}$ ± SE	4.48 ± 0.06 ^a^	3.83 ± 0.05 ^a^	3.99 ± 0.04 ^b^	4.37 ± 0.11 ^b^
	Q_1_	4.34	3.64	3.87	4.02
	Q_2_ (IQR)	4.53 (0.28)	3.84 (0.31)	4.02 (0.27)	4.34 (0.68)
	Q_3_	4.61	3.95	4.15	4.70
	ZAB vs. ROS vs. Ant.	a	c	b	
	Nat. vs. SIL vs. SOP	ab		b	a
	Nat. vs. Ant.	do not differ significantly			
Length of petal (LP) (mm)	$\bar{x}$ ± SE	9.38 ± 0.12 ^a^	8.18 ± 0.11 ^b^	9.27 ± 0.10 ^a^	9.36 ± 0.23 ^a^
	Q_1_	9.06	7.74	8.77	8.63
	Q_2_ (IQR)	9.41 (0.74)	8.03 (0.95)	9.29 (0.86)	9.31 (1.34)
	Q_3_	9.80	8.70	9.63	9.97
	ZAB vs. ROS vs. Ant.	a	b	a	
Width of petal (WP) (mm)	$\bar{x}$ ± SE	4.01 ± 0.05 ^a^	3.44 ± 0.06 ^b^	3.83 ± 0.06 ^a^	3.78 ± 0.11 ^a^
	Q_1_	3.89	3.18	3.69	3.48
	Q_2_ (IQR)	4.06 (0.29)	3.38 (0.42)	3.86 (0.34)	3.67 (0.53)
	Q_3_	4.17	3.60	4.03	4.01
	ZAB vs. ROS vs. Ant.	a	c	b	
Length of lateral sepal (LLS) (mm)	$\bar{x}$ ± SE	11.34 ± 0.13	11.01 ± 0.12	11.35 ± 0.10	12.80 ± 0.19
	Q_1_	10.82	10.53	11.05	11.93
	Q_2_ (IQR)	11.35 (1.01) ^BC^	10.87 (0.91) ^C^	11.40 (0.65) ^B^	12.63 (1.36) ^A^
	Q_3_	11.81	11.44	11.70	13.29
	Nat. vs. SIL vs. SOP	B		B	A
Width of lateral sepal (WLS) (mm)	$\bar{x}$ ± SE	4.17 ± 0.04	3.76 ± 0.04	3.83 ± 0.04	4.33 ± 0.10
	Q_1_	4.03	3.63	3.69	3.96
	Q_2_ (IQR)	4.15 (0.29) ^B^	3.71 (0.34) ^A^	3.88 (0.32) ^A^	4.18 (0.56) ^B^
	Q_3_	4.32	3.96	4.01	4.52
Width of flowers (FW) (mm)	$\bar{x}$ ± SE	22.70 ± 0.35	20.83 ± 0.24	22.59 ± 0.16	24.31 ± 0.38
	Q_1_	22.26	20.22	21.87	22.66
	Q_2_ (IQR)	22.94 (1.64) ^B^	20.89 (1.43) ^A^	22.47 (1.40) ^B^	24.01 (2.70) ^C^
	Q_3_	23.90	21.65	23.26	25.37
Length of flowers (FH) (mm)	$\bar{x}$ ± SE	11.47 ± 0.14 ^ab^	10.36 ± 0.10 ^c^	11.01 ± 0.12 ^b^	11.94 ± 0.22 ^a^
	Q_1_	11.04	10.02	10.69	11.33
	Q_2_ (IQR)	11.45 (0.83)	10.35 (0.76)	10.97 (0.77)	11.82 (1.30)
	Q_3_	11.86	10.78	11.46	12.63
Length of labellum (LL) (mm)	$\bar{x}$ ± SE	11.91 ± 0.16	11.46 ± 0.15	11.45 ± 0.14	11.93 ± 0.26
	Q_1_	11.56	11.01	11.23	11.42
	Q_2_ (IQR)	12.06 (0.81) ^B^	11.47 (0.89) ^A^	11.54 (0.64) ^A^	11.99 (1.20) ^B^
	Q_3_	12.37	11.88	11.87	12.62
	ZAB vs. ROS vs. Ant.	A	B	AB	
Width of hypochile (HW) (mm)	$\bar{x}$ ± SE	5.81 ± 0.13 ^a^	4.90 ± 0.10 ^b^	4.84 ± 0.14 ^b^	5.54 ± 0.12 ^a^
	Q_1_	5.25	4.64	4.31	5.06
	Q_2_ (IQR)	5.79 (1.15)	4.95 (0.61)	4.69 (0.98)	5.53 (0.83)
	Q_3_	6.40	5.25	5.29	5.9
Length of hypochile (HL) (mm)	$\bar{x}$ ± SE	5.03 ± 0.13	4.58 ± 0.07	4.44 ± 0.06	4.80 ± 0.10
	Q_1_	4.62	4.38	4.20	4.36
	Q_2_ (IQR)	4.89 (0.61) ^C^	4.65 (0.36) ^AB^	4.47 (0.38) ^A^	4.76 (0.77) ^BC^
	Q_3_	5.23	4.74	4.58	5.12
Length of epichile (LE) (mm)	$\bar{x}$ ± SE	7.11 ± 0.08	6.74 ± 0.11	7.03 ± 0.09	7.44 ± 0.11
	Q_1_	6.87	6.38	6.82	7.14
	Q_2_ (IQR)	7.10 (0.51) ^B^	6.88 (0.74) ^A^	7.05 (0.44) ^B^	7.51 (0.64) ^C^
	Q_3_	7.38	7.12	7.26	7.78
Length of isthmus (LI) (mm)	$\bar{x}$ ± SE	2.07 ± 0.04 ^ab^	1.79 ± 0.03 ^ab^	1.94 ± 0.03 ^b^	2.24 ± 0.06 ^a^
	Q_1_	1.92	1.66	1.82	2.09
	Q_2_ (IQR)	2.08 (0.33)	1.79 (0.29)	1.95 (0.26)	2.31 (0.41)
	Q_3_	2.25	1.94	2.08	2.50
	Nat. vs. SIL vs. SOP	b		b	a
Width of epichile (WE) (mm)	$\bar{x}$ ± SE	7.98 ± 0.11 ^ab^	7.71 ± 0.09 ^ab^	7.56 ± 0.09 ^b^	8.07 ± 0.16 ^a^
	Q_1_	7.52	7.46	7.27	7.47
	Q_2_ (IQR)	7.89 (0.84)	7.66 (0.49)	7.51 (0.54)	8.11 (0.94)
	Q_3_	8.36	7.95	7.81	8.42
	Nat. vs. SIL vs. SOP	ab		b	a
Width of isthmus (WI) (mm)	$\bar{x}$ ± SE	0.84 ± 0.01	0.90 ± 0.02	0.82 ± 0.01	1.02 ± 0.03
	Q_1_	0.82	0.81	0.75	0.94
	Q_2_ (IQR)	0.85 (0.06) ^B^	0.91 (0.15) ^A^	0.82 (0.11) ^B^	1.01 (0.13) ^C^
	Q_3_	0.88	0.96	0.86	1.07
Isthmus area (AI) (mm^2^)	$\bar{x}$ ± SE	1.75 ± 0.05 ^b^	1.60 ± 0.05 ^b^	1.59 ± 0.05 ^b^	2.32 ± 0.11 ^a^
	Q_1_	1.65	1.42	1.41	1.94
	Q_2_ (IQR)	1.75 (0.29)	1.58 (0.37)	1.54 (0.34)	2.28 (0.71)
	Q_3_	1.94	1.79	1.75	2.65
	Nat. vs. SIL vs. SOP	b		b	a

**Table 2:** The concentration of amino acids (μM) and total amount of amino acids (mg/mL) in *Epipactis palustris* nectar. The number of classes represents the effect of amino acids on insect chemoreceptors: I—no effect; II—inhibition of chemoreceptors; III—stimulate the salt cell; IV—the ability to stimulate the sugar cell. Data (*n* = 30) represent the mean ($\bar{x}$) ± standard error (SE), lower quartile (Q_1_), median (Q_2_), upper quartile (Q_3_), and interquartile range (IQR). Different lowercase letters indicate statistically significant differences, according to Tukey’s post-hoc test (*p* < 0.05). Different uppercase letters indicate statistically significant differences according to the pairwise Wilcoxon Rank Sum test with Benjamini–Hochberg adjustment (*p* < 0.05). ND—not detected. Additional comparisons were shown only when populations within Nat. or Ant. (or both) do not differ significantly.

Amino Acid	Class	Statistic	Natural (Nat.) Populations		Anthropogenic (Ant.) Populations	
			ZAB	ROS	SIL	SOP
Proteogenic amino acids (μM)						
Aspartic acid (Asp)	I	$\bar{x}$ ± SE	357.55 ± 6.47	377.50 ± 6.73	200.25 ± 6.81	259.51 ± 5.06
		Q_1_	329.36	351.40	178.80	228.94
		Q_2_ (IQR)	359.31 (56.82) ^B^	380.49 (50.09) ^A^	198.98 (48.50) ^D^	267.79 (49.53) ^C^
		Q_3_	386.17	401.49	227.30	278.48
Glutamic acid (Glu)	I	$\bar{x}$ ± SE	740.90 ± 8.71 ^b^	884.43 ± 7.46 ^a^	430.24 ± 6.87 ^d^	525.11 ± 4.78 ^c^
		Q_1_	706.06	858.27	400.67	509.22
		Q_2_ (IQR)	730.92 (71.49)	885.59 (48.87)	421.47 (64.14)	522.14 (24.38)
		Q_3_	777.55	907.13	464.81	533.60
Alanine (Ala)	I	$\bar{x}$ ± SE	92.59 ± 2.48	116.35 ± 1.74	84.42 ± 1.10	89.31 ± 2.40
		Q_1_	80.82	111.75	79.64	81.50
		Q_2_ (IQR)	89.79 (23.47) ^B^	117.99 (9.50) ^A^	84.07 (9.36) ^C^	83.99 (12.90) ^C^
		Q_3_	104.29	121.25	89.01	94.40
		ZAB vs. ROS vs. Ant.	B	A	B	
Cysteine (Cys)	I	$\bar{x}$ ± SE	163.35 ± 3.07	229.32 ± 3.52	149.33 ± 3.41	93.10 ± 1.10
		Q_1_	152.41	217.42	136.75	89.49
		Q_2_ (IQR)	168.28 (20.05) ^B^	229.17 (17.12) ^A^	143.13 (26.49) ^B^	92.88 (7.34) ^C^
		Q_3_	172.46	234.54	163.24	96.83
Glycine (Gly)	I	$\bar{x}$ ± SE	68.81 ± 1.92	88.60 ± 1.03	69.36 ± 1.60	57.25 ± 0.69
		Q_1_	61.34	83.57	63.75	54.38
		Q_2_ (IQR)	71.38 (16.50) ^B^	88.39 (9.56) ^A^	66.38 (7.20) ^B^	56.96 (6.03) ^C^
		Q_3_	77.83	93.13	70.96	60.41
Serine (Ser)	I	$\bar{x}$ ± SE	273.61 ± 3.77	326.20 ± 2.31	225.79 ± 2.77	184.75 ± 1.71
		Q_1_	263.55	318.96	214.73	180.13
		Q_2_ (IQR)	275.68 (23.06) ^B^	326.89 (12.68) ^A^	227.17 (16.76) ^C^	185.01 (9.16) ^D^
		Q_3_	286.61	331.64	231.50	189.29
Threonine (Thr)	I	$\bar{x}$ ± SE	141.01 ± 4.28 ^a^	139.20 ± 1.34 ^a^	123.17 ± 1.01 ^b^	80.10 ± 0.99 ^c^
		Q_1_	119.21	133.71	118.35	76.39
		Q_2_ (IQR)	139.94 (41.64)	139.5 (9.77)	122.99 (9.15)	80.38 (7.99)
		Q_3_	160.85	143.48	127.49	84.38
		Nat. vs. SIL vs. SOP	a		b	c
Tyrosine (Tyr)	I	$\bar{x}$ ± SE	12.29 ± 0.43	15.35 ± 0.34	6.91 ± 0.40	6.67 ± 0.41
		Q_1_	10.49	13.64	6.41	5.64
		Q_2_ (IQR)	12.89 (2.81) ^B^	15.39 (3.48) ^A^	7.41 (1.91) ^C^	7.38 (2.63) ^C^
		Q_3_	13.30	17.12	8.32	8.28
		ZAB vs. ROS vs. Ant.	B	A	C	
Arginine (Arg)	II	$\bar{x}$ ± SE	24.84 ± 0.79 ^b^	34.94 ± 0.42 ^a^	15.65 ± 0.29 ^c^	16.15 ± 0.42 ^c^
		Q_1_	21.95	32.70	14.42	14.39
		Q_2_ (IQR)	24.59 (5.29)	34.39 (3.80)	16.13 (2.08)	16.39 (3.10)
		Q_3_	27.25	36.50	16.49	17.49
		ZAB vs. ROS vs. Ant.	b	a	c	
Asparagine (Asn)	II	$\bar{x}$ ± SE	313.16 ± 4.99	432.35 ± 2.86	246.77 ± 3.26	237.69 ± 1.99
		Q_1_	302.5	421.59	232.62	228.42
		Q_2_ (IQR)	317.31 (25.06) ^B^	430.94 (20.08) ^A^	241.48 (31.30) ^C^	238.41 (15.70) ^C^
		Q_3_	327.56	441.67	263.92	244.12
		ZAB vs. ROS vs. Ant.	B	A	C	
Glutamine (Gln)	II	$\bar{x}$ ± SE	529.06 ± 6.84 ^b^	623.95 ± 5.04 ^a^	414.67 ± 2.64 ^c^	351.19 ± 3.72 ^d^
		Q_1_	509.06	600.73	406.49	332.55
		Q_2_ (IQR)	521.50 (30.51)	623.48 (41.83)	412.54 (15.84)	349.53 (31.96)
		Q_3_	539.57	642.56	422.33	364.51
Histidine (His)	II	$\bar{x}$ ± SE	133.04 ± 3.02 ^a^	101.81 ± 1.01 ^b^	71.05 ± 1.10 ^c^	68.62 ± 0.71 ^c^
		Q_1_	128.42	97.40	66.89	65.64
		Q_2_ (IQR)	132.50 (15.12)	102.48 (8.75)	69.65 (8.37)	68.49 (5.53)
		Q_3_	143.54	106.15	75.27	71.17
		ZAB vs. ROS vs. Ant.	a	b	c	
Lysine (Lys)	II	$\bar{x}$ ± SE	167.59 ± 3.61	231.48 ± 2.75	128.46 ± 1.29	115.63 ± 0.74
		Q_1_	152.75	219.45	124.67	113.68
		Q_2_ (IQR)	162.49 (25.32) ^B^	230.45 (18.80) ^A^	128.49 (7.92) ^C^	116.39 (4.53) ^D^
		Q_3_	178.07	238.24	132.60	118.21
Proline (Pro)	III	$\bar{x}$ ± SE	87.53 ± 2.92 ^b^	82.45 ± 2.56 ^b^	362.10 ± 7.85 ^a^	373.20 ± 8.13 ^a^
		Q_1_	76.60	71.53	331.58	335.57
		Q_2_ (IQR)	89.14 (25.72)	83.13 (18.10)	374.09 (69.02)	380.04 (69.61)
		Q_3_	102.32	89.62	400.60	405.18
		ZAB vs. ROS vs. Ant.	b	b	a	
		Nat. vs. SIL vs. SOP	b		a	a
		Nat. vs. Ant.	differ significantly			
Isoleucine (Ile)	IV	$\bar{x}$ ± SE	162.45 ± 3.79 ^a^	143.93 ± 1.61 ^b^	117.30 ± 1.60 ^c^	100.93 ± 0.62 ^d^
		Q_1_	148.25	138.68	111.08	98.77
		Q_2_ (IQR)	162.48 (30.83)	144.89 (12.13)	118.29 (10.70)	101.39 (4.52)
		Q_3_	179.08	150.80	121.78	103.28
Leucine (Leu)	IV	$\bar{x}$ ± SE	333.40 ± 4.77 ^a^	290.65 ± 3.82 ^b^	240.59 ± 3.13 ^c^	242.72 ± 1.78 ^c^
		Q_1_	312.63	278.40	224.67	237.50
		Q_2_ (IQR)	331.99 (31.35	291.74 (24.45)	239.15 (28.87)	243.39 (11.74)
		Q_3_	343.97	302.85	253.54	249.23
		ZAB vs. ROS vs. Ant.	a	b	c	
Methionine (Met)	IV	$\bar{x}$ ± SE	92.10 ± 3.11 ^a^	87.79 ± 0.79 ^a^	67.46 ± 0.66 ^b^	50.42 ± 0.79 ^c^
		Q_1_	79.01	84.48	66.03	47.66
		Q_2_ (IQR)	89.19 (24.54)	87.33 (5.79)	67.43 (3.47)	50.20 (5.55)
		Q_3_	103.55	90.27	69.50	53.21
		Nat. vs. SIL vs. SOP	a		b	c
Phenylalanine (Phe)	IV	$\bar{x}$ ± SE	44.87 ± 1.66 ^ab^	46.20 ± 0.61 ^ab^	48.72 ± 1.17 ^a^	42.50 ± 0.89 ^b^
		Q_1_	37.49	43.47	44.35	39.93
		Q_2_ (IQR)	44.95 (14.70)	46.42 (4.90)	49.4 (8.94)	43.38 (6.41)
		Q_3_	52.19	48.37	53.29	46.33
		Nat. vs. SIL vs. SOP	ab		a	b
Tryptophan (Trp)	IV	$\bar{x}$ ± SE	189.82 ± 3.71	255.90 ± 2.97	145.15 ± 1.20	128.45 ± 1.22
		Q_1_	176.42	244.04	140.58	124.39
		Q_2_ (IQR)	188.14 (25.84) ^B^	254.37 (22.42) ^A^	145.04 (9.16) ^C^	127.39 (5.84) ^D^
		Q_3_	202.26	266.46	149.74	130.23
Valine (Val)	IV	$\bar{x}$ ± SE	76.77 ± 1.51 ^a^	69.89 ± 0.94 ^b^	49.85 ± 0.82 ^c^	47.51 ± 0.83 ^c^
		Q_1_	71.41	66.29	46.37	44.62
		Q_2_ (IQR)	77.69 (12.03)	68.52 (8.12)	50.05 (6.13)	47.39 (6.46)
		Q_3_	83.44	74.40	52.50	51.08
		ZAB vs. ROS vs. Ant.	a	b	c	
Non-proteogenic amino acids (μM)						
Ornithine (Orn)		$\bar{x}$ ± SE	94.95 ± 3.81	116.33 ± 2.55	67.20 ± 1.24	ND
		Q_1_	78.61	109.38	64.80	ND
		Q_2_ (IQR)	89.72 (37.50) ^B^	114.95 (9.11) ^A^	68.54 (6.44) ^C^	ND
		Q_3_	116.11	118.50	71.24	ND
Citrulline (Cit)		$\bar{x}$ ± SE	4.15 ± 0.49	ND	ND	ND
		Q_1_	2.48	ND	ND	ND
		Q_2_ (IQR)	4.45 (3.89)	ND	ND	ND
		Q_3_	6.38	ND	ND	ND
Taurine (Tau)		$\bar{x}$ ± SE	16.18 ± 0.67	14.98 ± 0.51	10.32 ± 0.28	7.61 ± 0.18
		Q_1_	13.92	12.59	9.38	6.60
		Q_2_ (IQR)	15.69 (2.84) ^A^	14.39 (3.90) ^A^	10.18 (2.11) ^B^	7.51 (1.90) ^C^
		Q_3_	16.76	16.50	11.50	8.50
α-aminobutyric acid (AABA)		$\bar{x}$ ± SE	11.67 ± 0.42 ^a^	9.34 ± 0.36 ^b^	7.04 ± 0.30 ^c^	10.41 ± 0.34 ^c^
		Q_1_	9.95	8.40	5.50	8.91
		Q_2_ (IQR)	11.50 (2.84)	8.57 (2.13)	7.45 (2.24)	10.02 (2.98)
		Q_3_	12.8	10.53	7.74	11.89
		ZAB vs. ROS vs. Ant.	a	b	c	
		Nat. vs. SIL vs. SOP	a		b	b
		Nat. vs. Ant.	differ significantly			
β-aminobutyric acid (BABA)		$\bar{x}$ ± SE	14.01 ± 0.38	21.74 ± 0.64	8.55 ± 0.31	4.55 ± 0.23
		Q_1_	12.51	18.76	7.52	3.58
		Q_2_ (IQR)	13.68 (2.26) ^B^	21.08 (4.52) ^A^	8.54 (1.84) ^D^	4.4 (1.92) ^C^
		Q_3_	14.77	23.28	9.36	5.50
		ZAB vs. ROS vs. Ant.	B	A	C	
γ-aminobutyric acid (GABA)		$\bar{x}$ ± SE	6.84 ± 0.55	7.29 ± 0.35	3.91 ± 0.42	ND
		Q_1_	6.54	5.50	2.52	ND
		Q_2_ (IQR)	7.89 (2.12) ^A^	6.96 (3.01) ^A^	3.72 (2.98) ^B^	ND
		Q_3_	8.66	8.50	5.5	ND
		ZAB vs. ROS vs. Ant.	A	A	B	
		Nat. vs. SIL vs. SOP	A		B	B
		Nat. vs. Ant.	differ significantly			
β-alanine (β-Ala)		$\bar{x}$ ± SE	22.81 ± 1.12	16.25 ± 0.53	ND	ND
		Q_1_	18.46	14.52	ND	ND
		Q_2_ (IQR)	21.53 (8.88) ^A^	16.04 (2.96) ^B^	ND	ND
		Q_3_	27.34	17.48	ND	ND
Total amount of amino acids (mg/mL)						
		$\bar{x}$ ± SE	0.52 ± 0.003 ^b^	0.60 ± 0.002 ^a^	0.41 ± 0.002 ^c^	0.39 ± 0.002 ^d^
		Q_1_	0.51	0.59	0.41	0.39
		Q_2_ (IQR)	0.52 (0.01)	0.60 (0.01)	0.42 (0.01)	0.39 (0.01)
		Q_3_	0.53	0.60	0.42	0.40

**Table 3:** Spatial and temporal variation of female reproductive success (FRS) and pollinaria removal (PR) in *Epipactis palustris* populations. Data (*n* = 30) show the mean ± standard error.

Parameter	Natural Populations		Anthropogenic Populations	
	ZAB	ROS	SIL	SOP
FRS (%)	94.40 ± 2.83	81.47 ± 4.19	87.99 ± 3.36	90.60 ± 2.49
PR (%)	97.03 ± 2.68	87.48 ± 3.46	85.66 ± 4.36	96.55 ± 1.61
PR/FRS	1.07 ± 0.06	1.32 ± 0.22	1.07 ± 0.10	1.08 ± 0.03

**Table 4:** Measured flower structure properties.

Abbreviation	Full Name
AI	isthmus area
FH	length of flowers
FW	width of flowers
HL	length of hypochile
HW	width of hypochile
LDS	length of dorsal sepal
LE	length of epichile
LI	length of isthmus
LL	length of labellum
LLS	length of lateral sepal
LP	length of petal
WDS	width of dorsal sepal
WE	width of epichile
WI	width of isthmus
WLS	width of lateral sepal
WP	width of petal

**Table 5:** Amino acids evaluated during HPLC analysis.

Abbreviation	Full Name
AABA	α-aminobutyric acid
Ala	alanine
Arg	arginine
Asn	asparagine
Asp	aspartic acid
BABA	β-aminobutyric acid
Cit	citrulline
Cys	cysteine
GABA	γ-aminobutyric acid
Gln	glutamine
Glu	glutamic acid
Gly	glycine
His	histidine
Ile	isoleucine
Leu	leucine
Lys	lysine
Met	methionine
Orn	ornithine
Phe	phenylalanine
Pro	proline
Ser	serine
Tau	taurine
Thr	threonine
Trp	tryptophan
Tyr	tyrosine
Val	valine
β-Ala	β-alanine

## Data Availability

Data is contained within the current article and supplementary material.

## Supplementary Materials

The following are available online at https://www.mdpi.com/article/10.3390/ijms222212164/s1.
