# Supplementary material for: Nectar Chemistry or Flower Morphology—What Is More Important for the Reproductive Success of Generalist Orchid Epipactis palustris in Natural and Anthropogenic Populations?
Source: Int J Mol Sci. 2021 Nov 10;22(22):12164. doi: 10.3390/ijms222212164 (PMC8618778; doi:10.3390/ijms222212164)
Supplement: Supplementary file 1 [file ijms-22-12164-s001.zip › ijms-1430445-supplementary.pdf]

Supplementary materials

Figure S1. Boxplots of floral display and flower structure in *Epipactis palustris* natural (Nat.) and anthropogenic (Ant.) populations (n = 30). Colored dots are individual samples. The crossed square shows the mean. The lower and upper hinges correspond to the lower (Q_1) and upper (Q_3) quartiles. Thus box length shows the interquartile range (IQR). The thicker line inside boxes corresponds to the median. The lower whisker extends from the hinge to the smallest value at most Q_1 - 1.5 × IQR of the hinge. The upper whisker extends from the hinge to the largest value no further than Q_3 + 1.5 × IQR. Data beyond the end of the whiskers, indicated with an asterisk symbol, are outliers. Different lowercase letters indicate statistically significant differences according to Tukey's post-hoc test ($p < 0.05$). Different uppercase letters indicate statistically significant differences according to the pairwise Wilcoxon Rank Sum test with Benjamini-Hochberg adjustment ($p < 0.05$). Symbol '=' means 'do not differ significantly'. Additional comparisons on the right side were shown only when populations within Nat. and/or Ant. do not differ significantly.

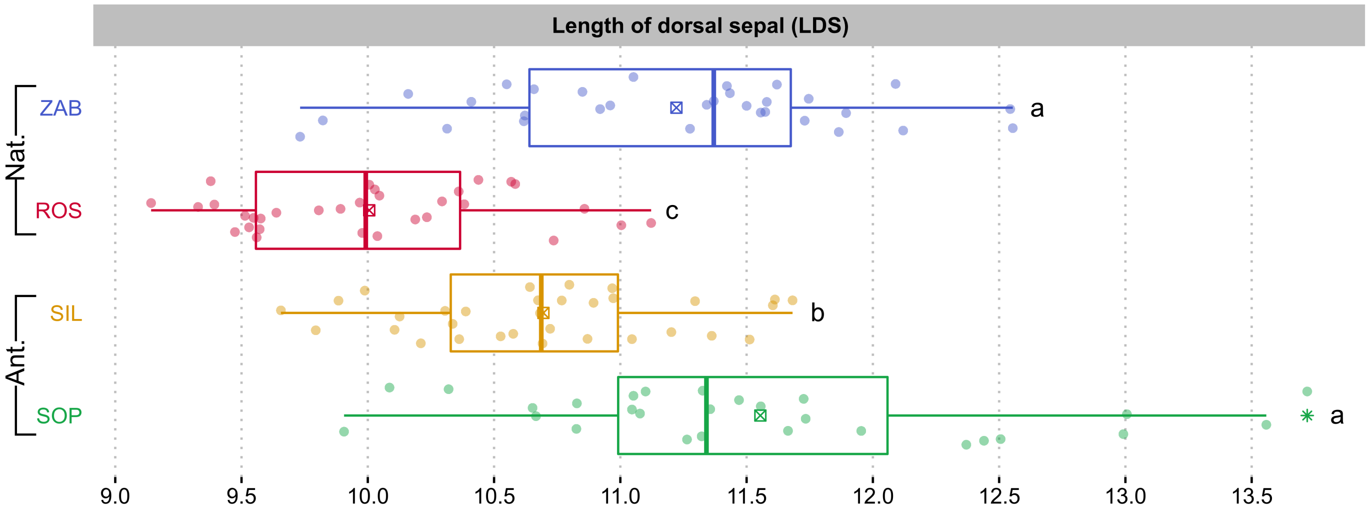

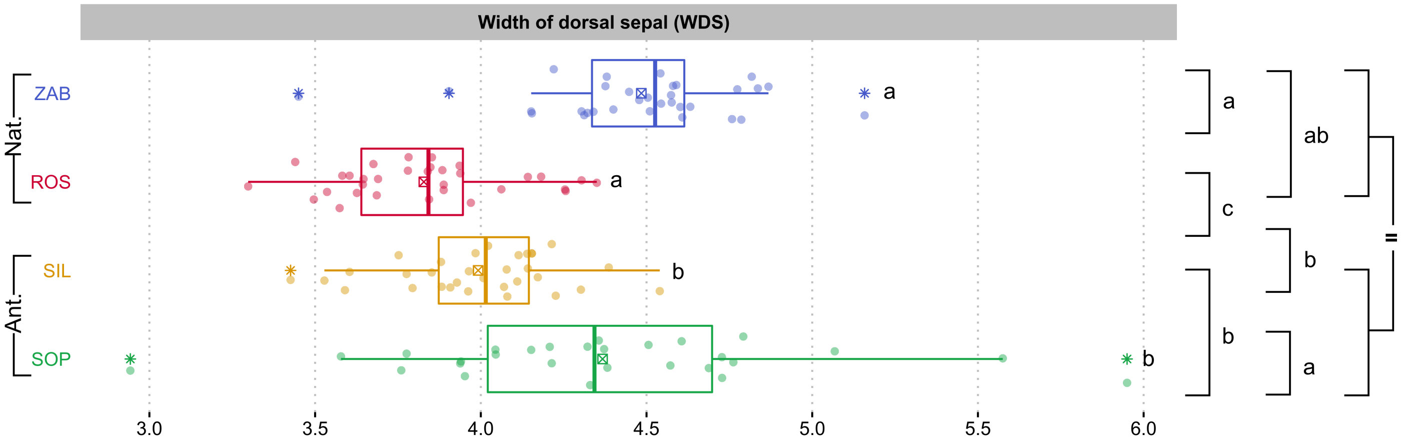

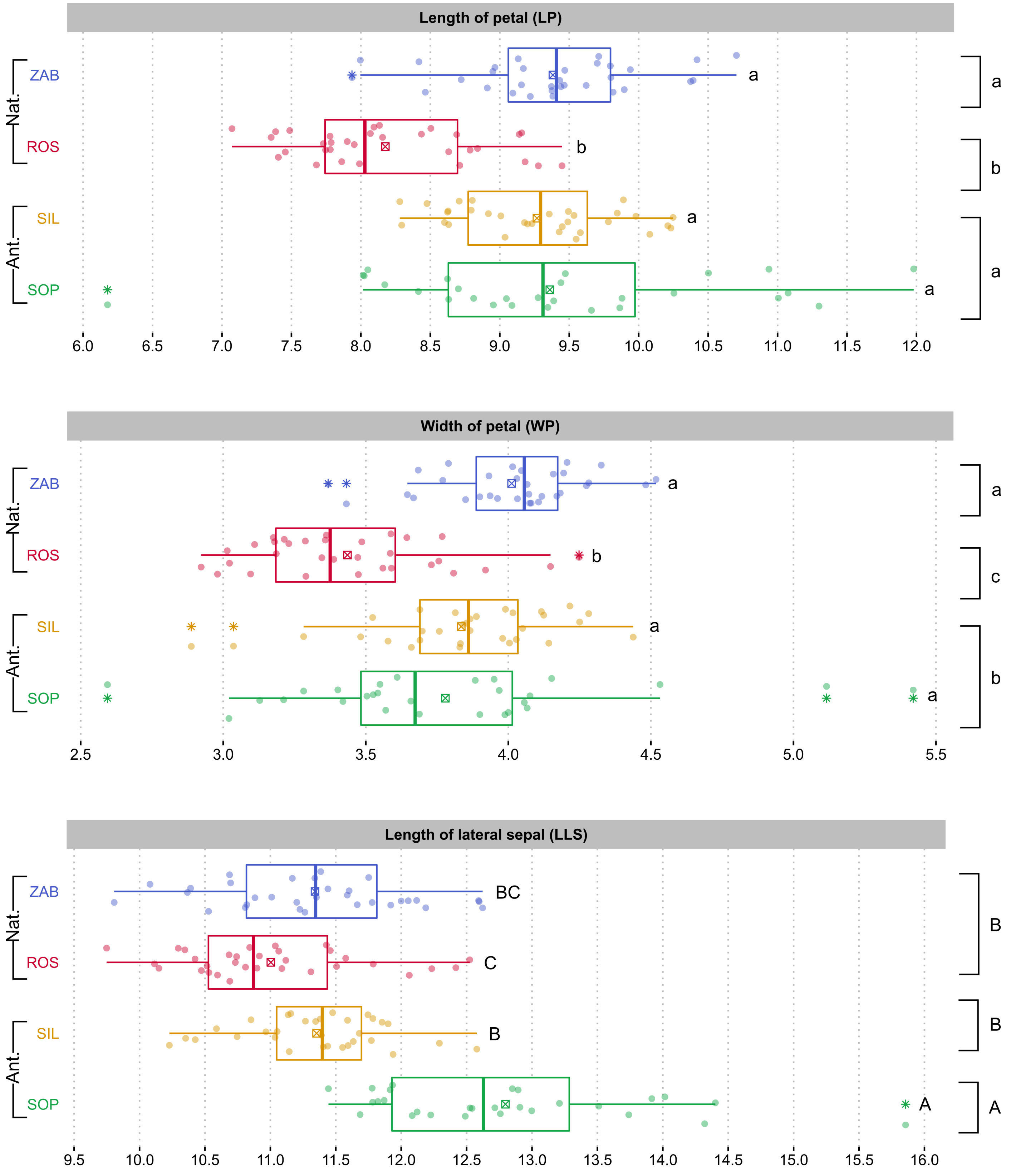
Length of petal (LP)
Nat.
ZAB
ROS
Ant.
SIL
SOP
a
b
a
a
a
b
a
6.0
6.5
7.0
7.5
8.0
8.5
9.0
9.5
10.0
10.5
11.0
11.5
12.0
Width of petal (WP)
Nat.
ZAB
ROS
Ant.
SIL
SOP
a
b
a
a
a
c
b
2.5
3.0
3.5
4.0
4.5
5.0
5.5
Length of lateral sepal (LLS)
Nat.
ZAB
ROS
Ant.
SIL
SOP
BC
C
B
A
B
B
A
9.5
10.0
10.5
11.0
11.5
12.0
12.5
13.0
13.5
14.0
14.5
15.0
15.5
16.0

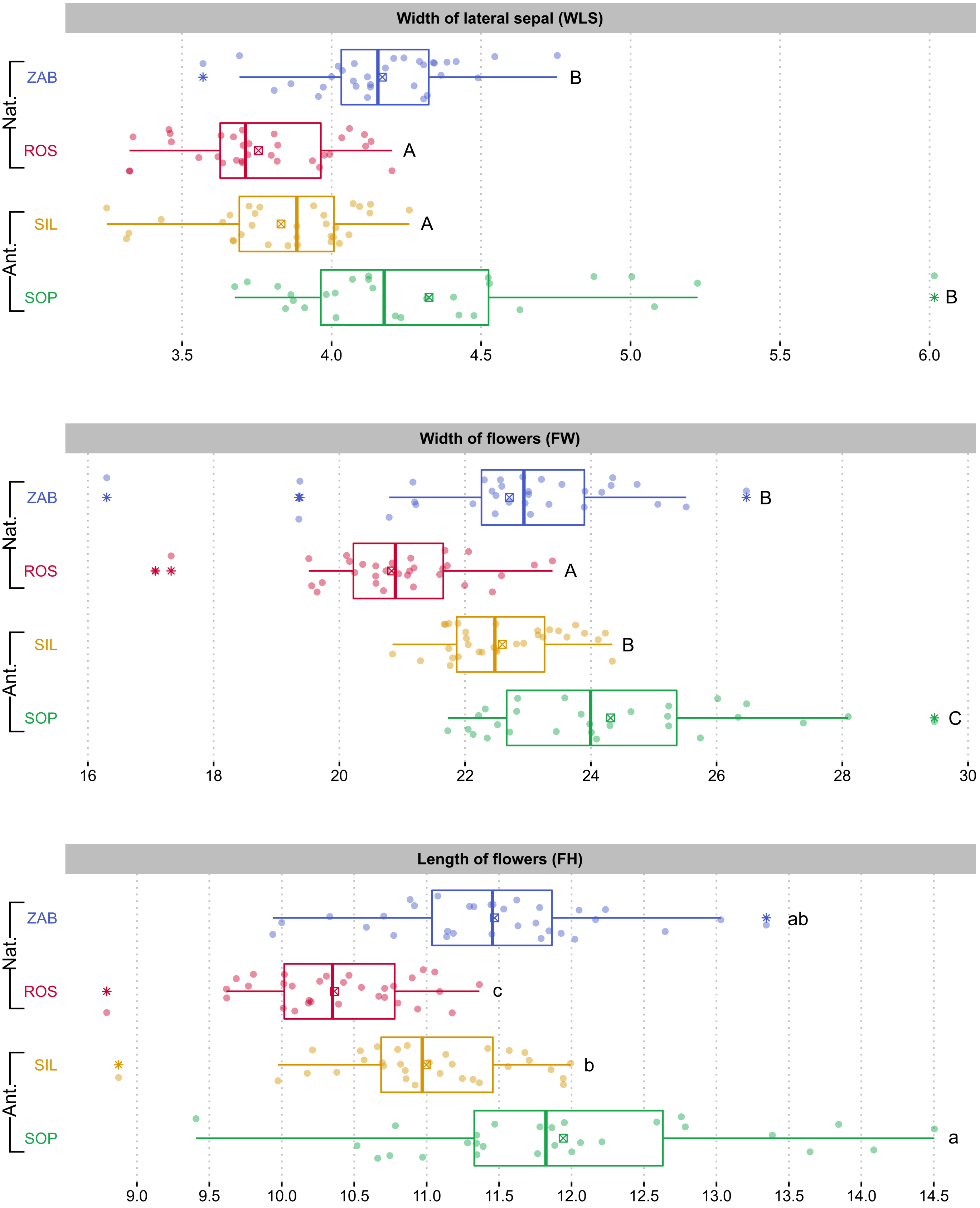
Width of lateral sepal (WLS)
Nat.
ZAB
ROS
Ant.
SIL
SOP
B
A
A
B
3.5
4.0
4.5
5.0
5.5
6.0
Width of flowers (FW)
ZAB
ROS
SIL
SOP
B
A
B
C
16
18
20
22
24
26
28
30
Length of flowers (FH)
ZAB
ROS
SIL
SOP
ab
c
b
a
9.0
9.5
10.0
10.5
11.0
11.5
12.0
12.5
13.0
13.5
14.0
14.5

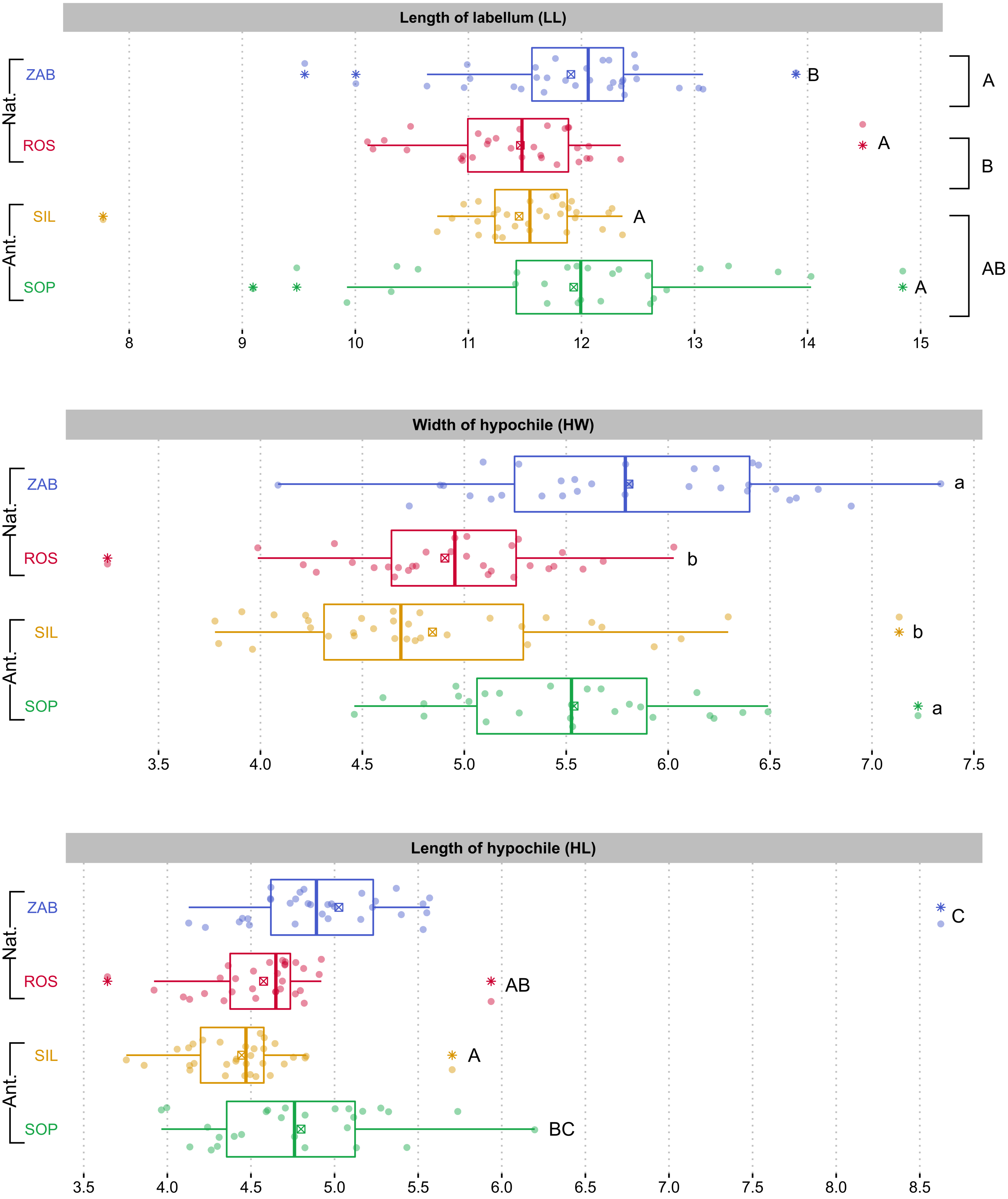
Length of labellum (LL)
ZAB
ROS
SIL
SOP
Nat.
Ant.
B
A
A
A
A
B
AB
8
9
10
11
12
13
14
15
Width of hypochile (HW)
ZAB
ROS
SIL
SOP
Nat.
Ant.
a
b
b
a
3.5
4.0
4.5
5.0
5.5
6.0
6.5
7.0
7.5
Length of hypochile (HL)
ZAB
ROS
SIL
SOP
Nat.
Ant.
C
AB
A
BC
3.5
4.0
4.5
5.0
5.5
6.0
6.5
7.0
7.5
8.0
8.5

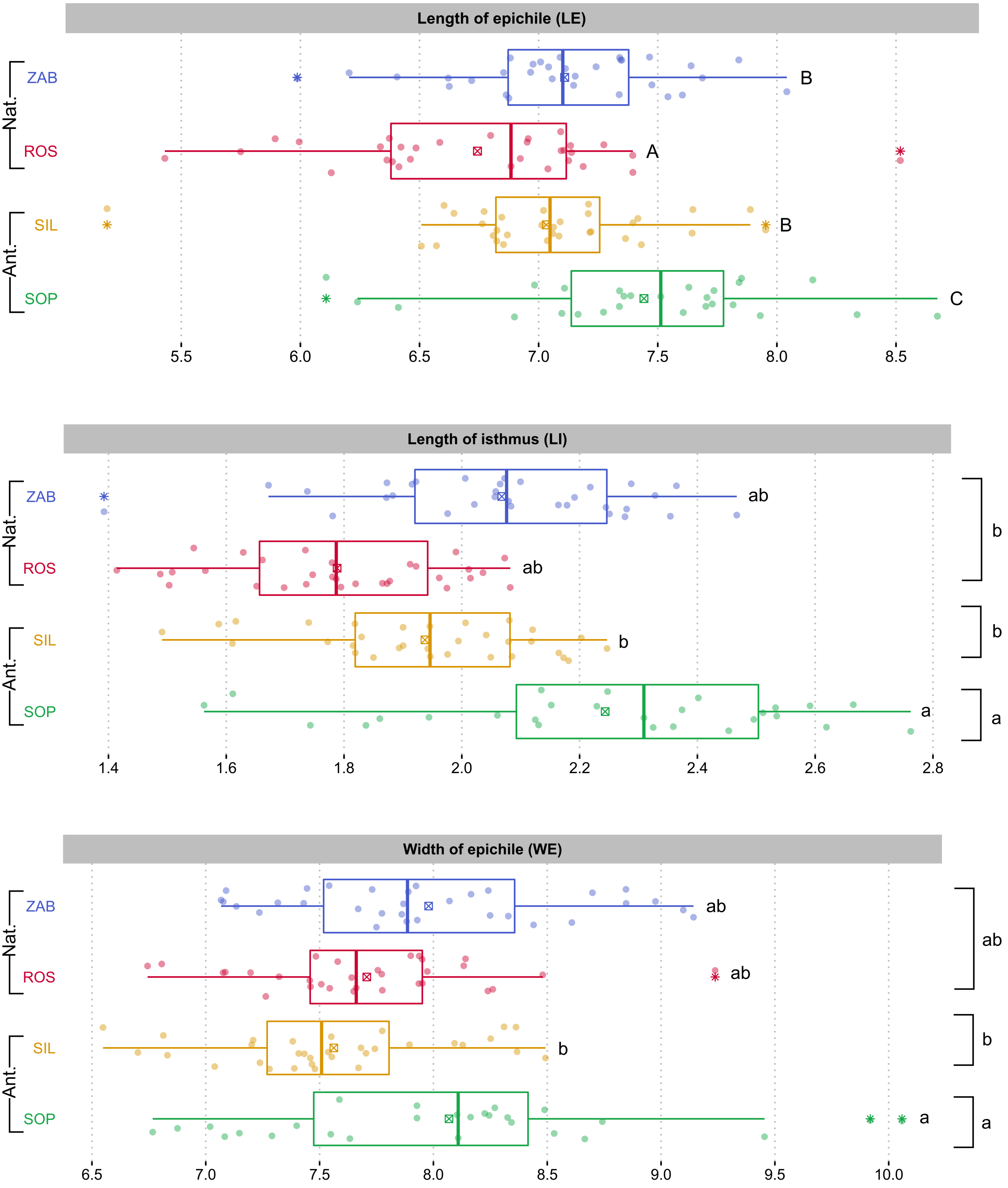
Length of epichile (LE)
Nat.
ZAB
ROS
Ant.
SIL
SOP
B
A
B
C
5.5
6.0
6.5
7.0
7.5
8.0
8.5
Length of isthmus (LI)
ab
ab
b
a
b
b
a
1.4
1.6
1.8
2.0
2.2
2.4
2.6
2.8
Width of epichile (WE)
ab
ab
b
a
ab
b
a
6.5
7.0
7.5
8.0
8.5
9.0
9.5
10.0

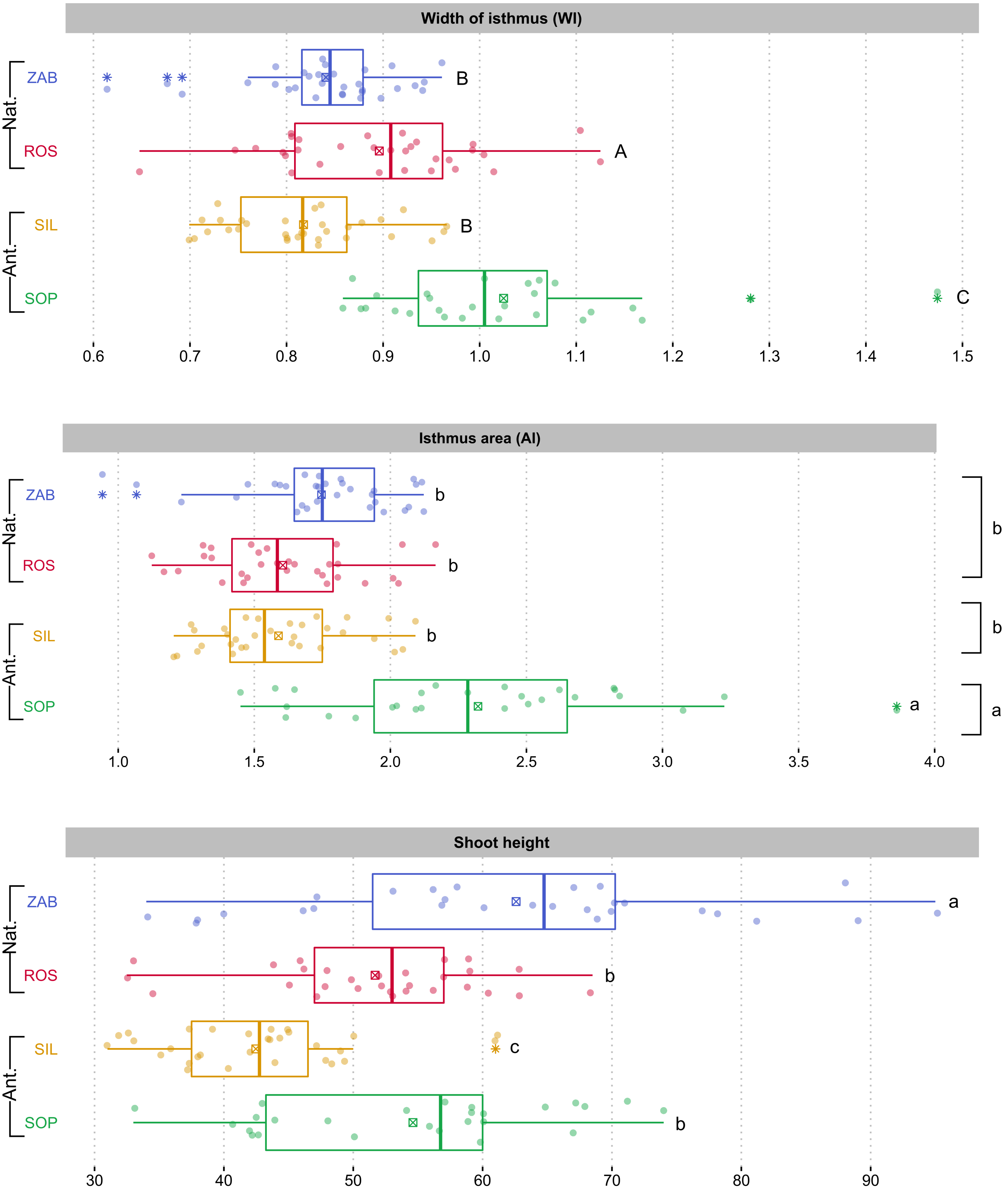
Width of isthmus (WI)
ZAB
ROS
SIL
SOP
Nat.
Ant.
B
A
B
C
0.6
0.7
0.8
0.9
1.0
1.1
1.2
1.3
1.4
1.5
Isthmus area (AI)
ZAB
ROS
SIL
SOP
Nat.
Ant.
b
b
b
a
b
b
a
1.0
1.5
2.0
2.5
3.0
3.5
4.0
Shoot height
ZAB
ROS
SIL
SOP
Nat.
Ant.
a
b
c
b
30
40
50
60
70
80
90

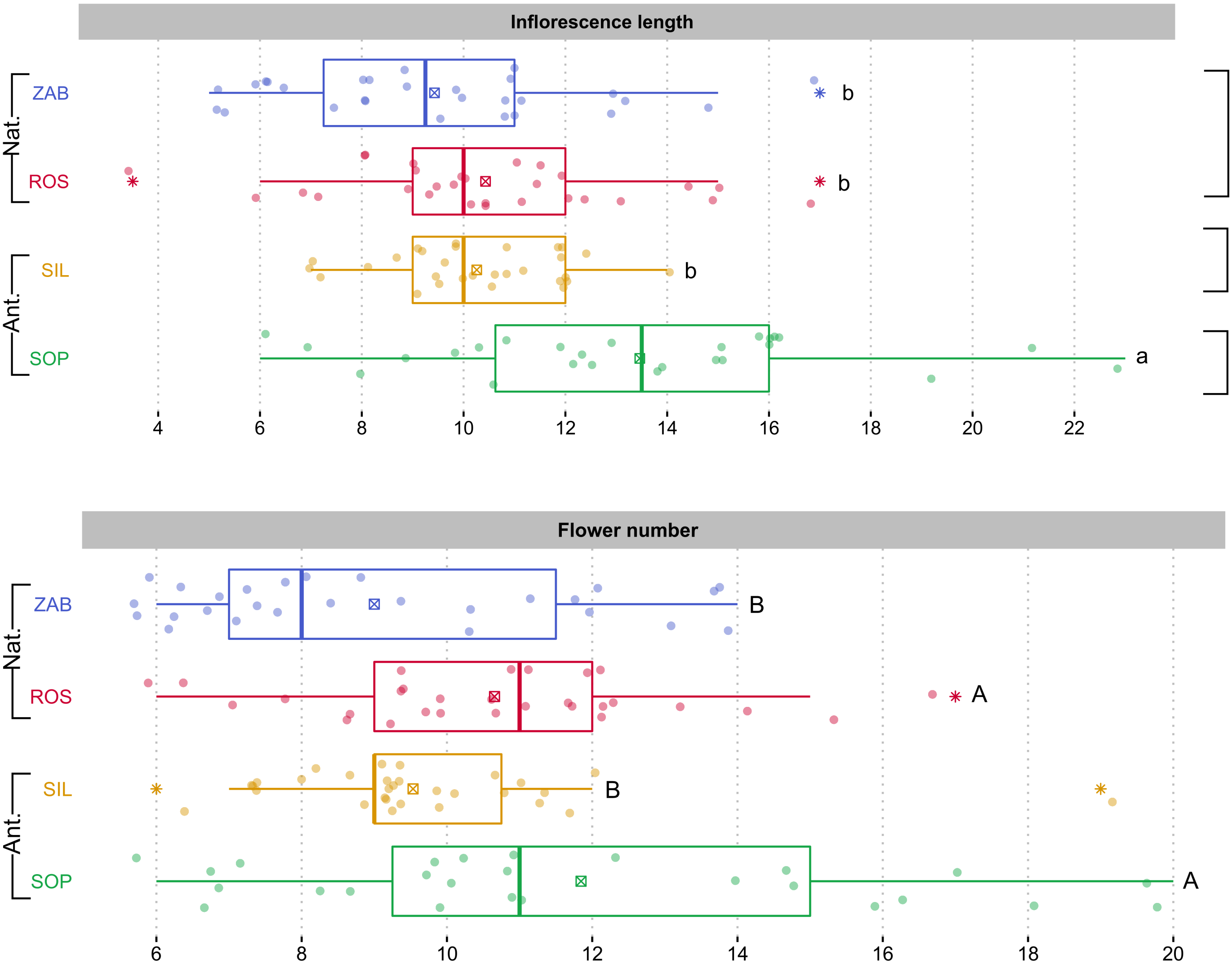
Inflorescence length
Nat.
Ant.
ZAB
ROS
SIL
SOP
b
b
b
a
b
b
a
4
6
8
10
12
14
16
18
20
22
Flower number
ZAB
ROS
SIL
SOP
B
A
B
A
6
8
10
12
14
16
18
20

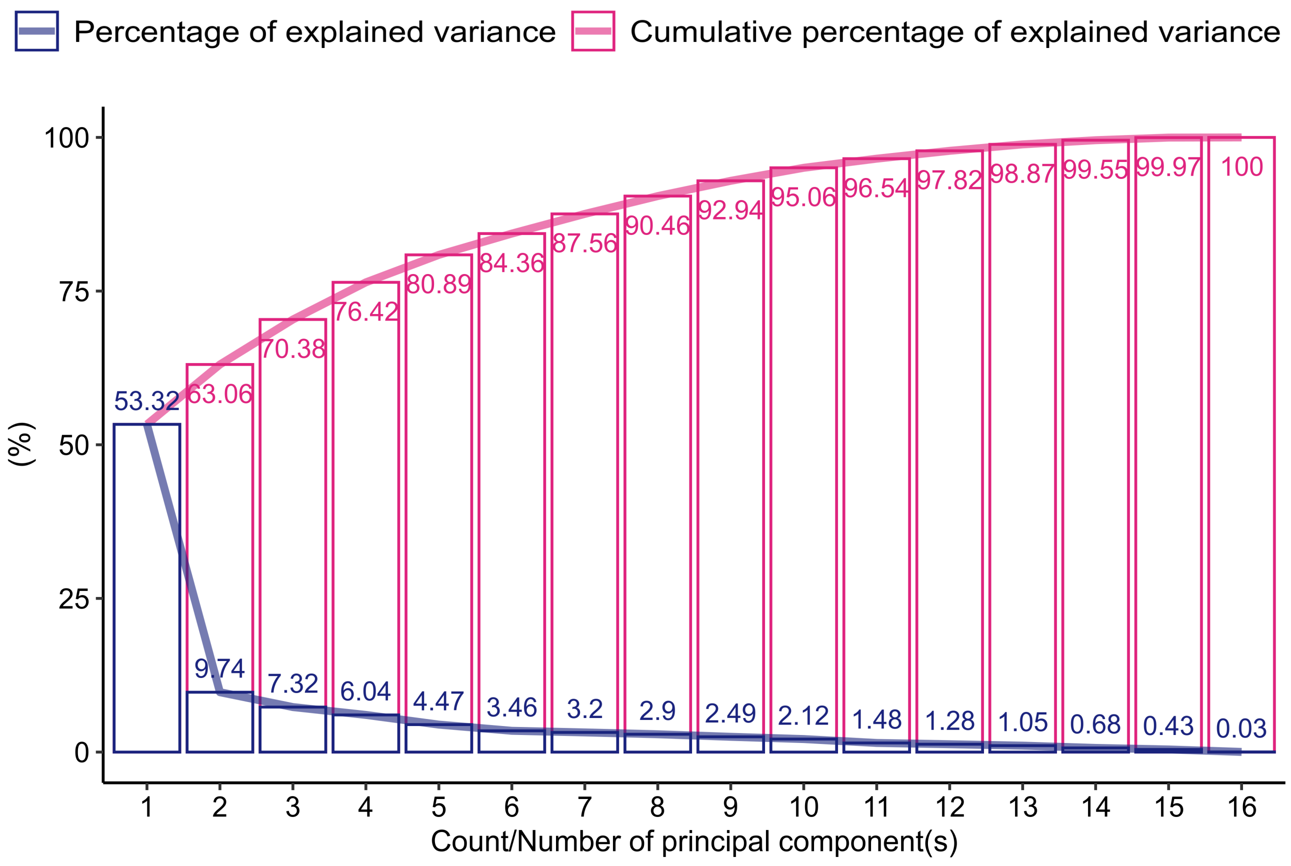

Figure S2. Scree plot showing the proportion of explained variance by the principal components of flower structure PCA model.

	LDS	WDS	LP	WP	LLS	WLS	FW	FH	LL	HW	HL	LE	LI	WE	WI	AI
Dim.1	0.797	0.711	0.633	0.48	0.646	0.578	0.646	0.773	0.48	0.289	0.195	0.498	0.472	0.378	0.365	0.59
Dim.2	0.072	0.043	0.15	0.147	0.005	0.029	0.045	0.07	0.041	0.116	0.04	0.002	0.169	0.027	0.303	0.299
Dim.3	0.01	0.018	0	0.094	0.148	0.007	0.071	0.016	0.095	0.059	0.402	0.006	0.012	0.096	0.088	0.051
Dim.4	0.001	0	0.037	0	0.001	0	0.004	0.001	0.013	0.218	0.032	0.213	0.109	0.288	0.047	0.003
Dim.5	0.008	0.008	0.001	0.08	0.001	0.079	0.02	0.004	0.098	0.149	0.096	0.074	0	0.001	0.067	0.029
Dim.6	0.003	0.019	0.012	0.067	0.105	0.001	0.024	0.01	0.001	0	0.198	0.001	0.084	0.014	0.003	0.012
Dim.7	0.001	0.057	0.037	0.009	0.003	0.168	0.014	0.003	0.003	0.083	0.027	0.027	0.059	0.003	0.007	0.012
Dim.8	0.012	0.005	0.039	0.024	0.003	0.025	0.018	0.002	0.104	0.001	0.004	0	0.052	0.105	0.07	0.001
Dim.9	0	0.001	0.005	0.024	0	0.082	0.006	0.001	0.094	0.066	0.003	0.009	0.036	0.036	0.036	0
Dim.10	0.002	0.013	0	0.001	0.003	0.002	0.03	0.007	0.069	0.012	0.001	0.162	0.001	0.036	0.001	0
Dim.11	0.055	0	0	0.012	0.013	0.003	0.073	0.074	0.001	0	0	0.001	0	0	0.003	0
Dim.12	0	0.112	0.003	0.042	0.009	0.011	0.012	0.005	0	0.007	0.002	0.001	0	0	0	0
Dim.13	0.002	0	0.057	0.013	0.034	0	0.03	0.005	0	0	0	0.004	0.001	0.017	0.003	0
Dim.14	0	0.012	0.026	0.007	0.03	0.014	0.004	0.001	0.001	0.001	0	0.002	0.004	0	0.006	0
Dim.15	0.038	0	0	0	0	0	0	0.029	0	0	0	0	0	0	0	0
Dim.16	0	0	0	0	0	0	0	0	0	0	0	0	0.001	0	0.001	0.003

Colour scale: 0.8, 0.72, 0.64, 0.56, 0.48, 0.4, 0.32, 0.24, 0.16, 0.08, 0

Figure S3. Cos² for the flower structure parameters selected as active variables in the principal component analysis model, representing the quality of representation for variables on the factor map (Dim1-16).

Figure S4. Boxplots of amino acids amounts for *Epipactis palustris* natural (Nat.) and anthropogenic (Ant.) populations (n = 30). Colored dots are individual samples. The crossed square shows the mean. The lower and upper hinges correspond to the lower (Q_1) and upper (Q_3) quartiles. Thus box length shows the interquartile range (IQR). The thicker line inside boxes corresponds to the median. The lower whisker extends from the hinge to the smallest value at most Q_1 - 1.5 × IQR of the hinge. The upper whisker extends from the hinge to the largest value no further than Q_3 + 1.5 × IQR. Data beyond the end of the whiskers, indicated with an asterisk symbol, are outliers. Different lowercase letters indicate statistically significant differences according to Tukey's post-hoc test ($p < 0.05$). Different uppercase letters indicate statistically significant differences according to the pairwise Wilcoxon Rank Sum test with Benjamini-Hochberg adjustment ($p < 0.05$). Symbol '≠' means 'differ significantly'. Additional comparisons on the right side were shown only when populations within Nat. and/or Ant. do not differ significantly.

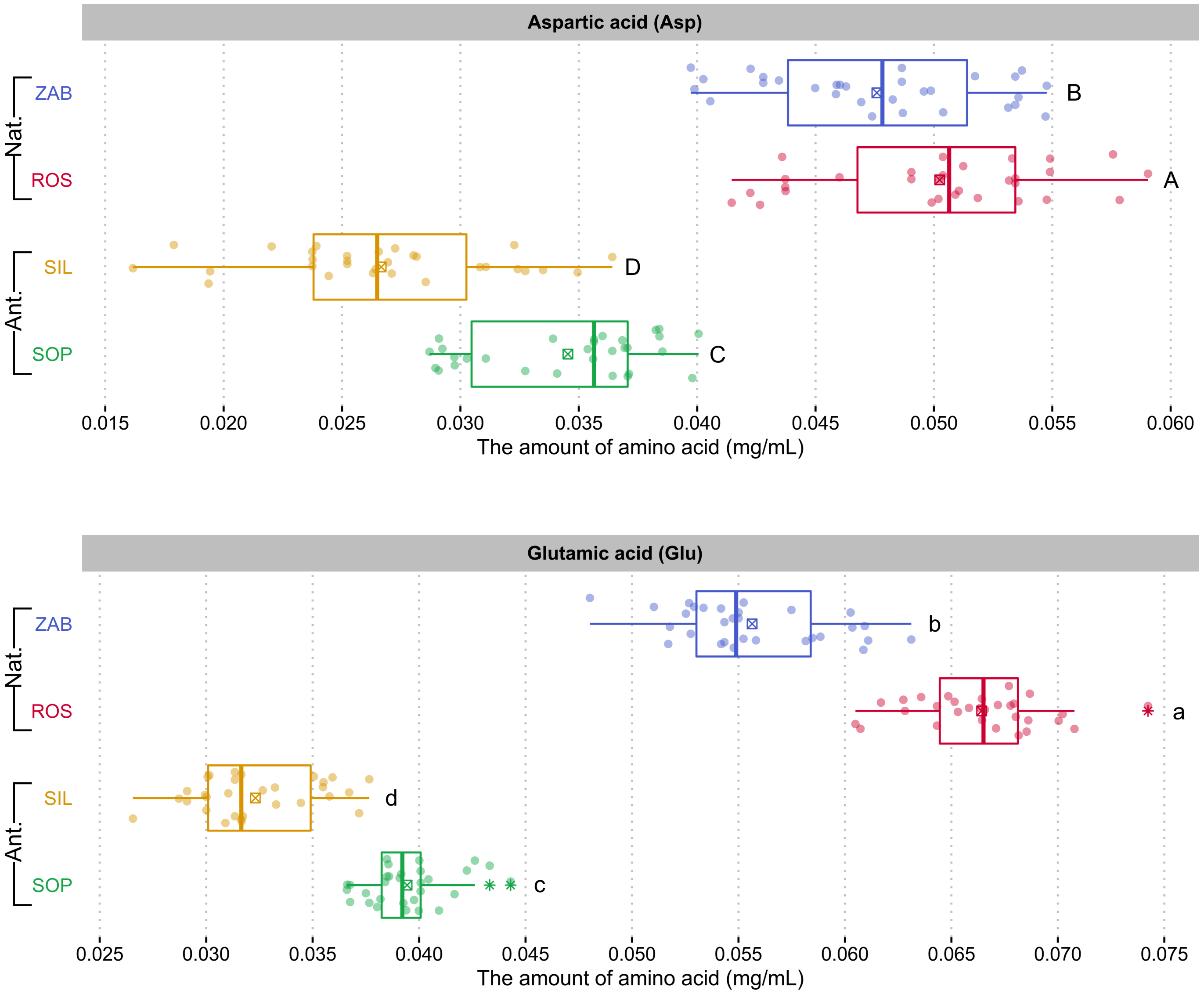

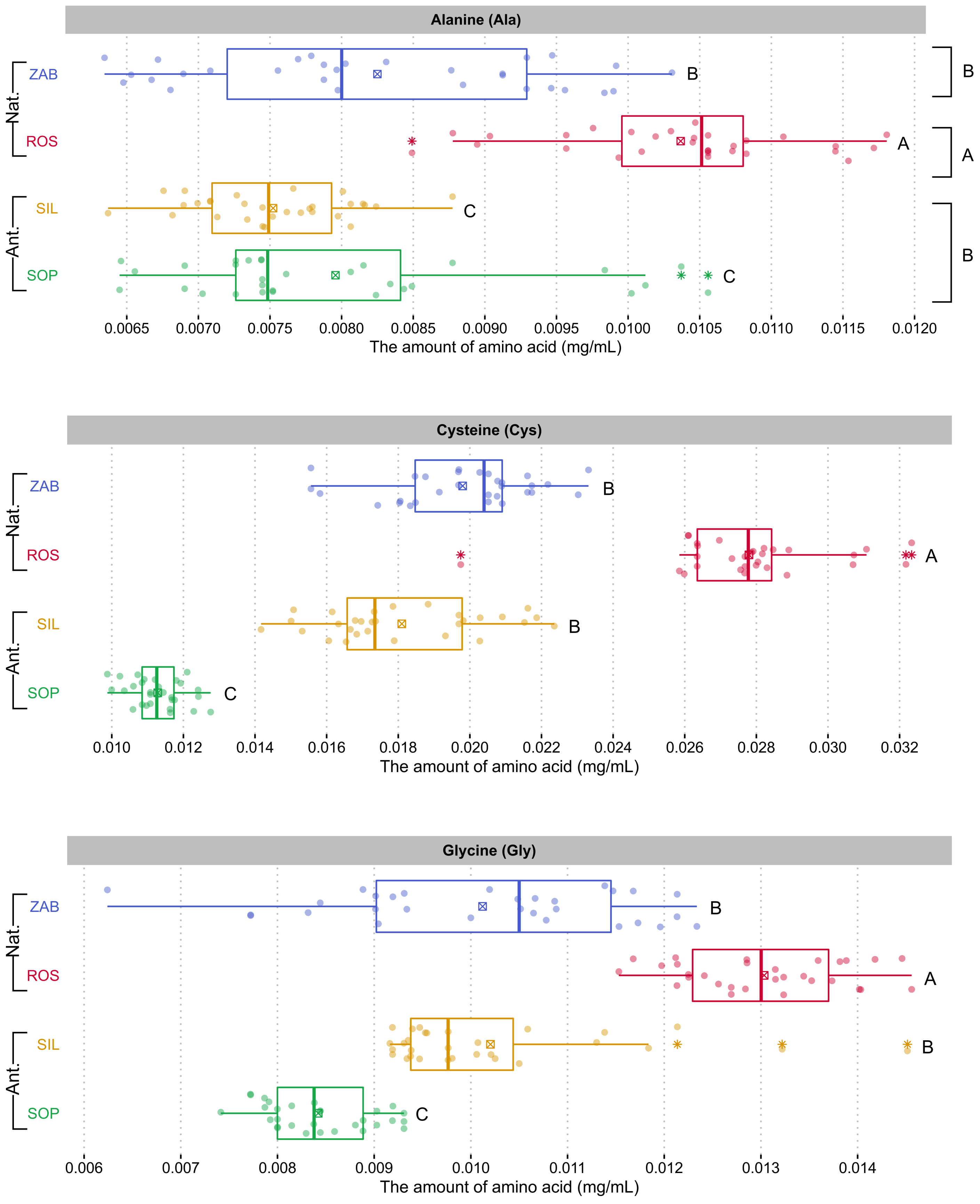
Alanine (Ala)
Nat.
ZAB
ROS
Ant.
SIL
SOP
A
B
C
0.0065
0.0070
0.0075
0.0080
0.0085
0.0090
0.0095
0.0100
0.0105
0.0110
0.0115
0.0120
The amount of amino acid (mg/mL)
Cysteine (Cys)
0.010
0.012
0.014
0.016
0.018
0.020
0.022
0.024
0.026
0.028
0.030
0.032
The amount of amino acid (mg/mL)
Glycine (Gly)
0.006
0.007
0.008
0.009
0.010
0.011
0.012
0.013
0.014
The amount of amino acid (mg/mL)

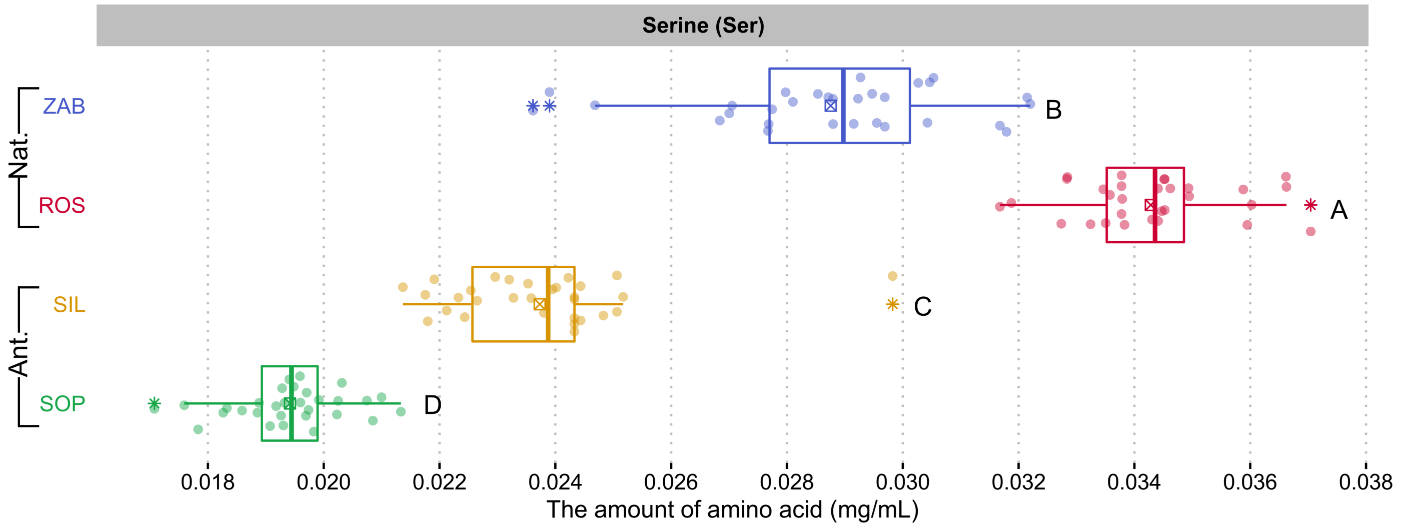
Serine (Ser)
Nat.
ZAB
ROS
Ant.
SIL
SOP
A
B
C
D
0.018
0.020
0.022
0.024
0.026
0.028
0.030
0.032
0.034
0.036
0.038
The amount of amino acid (mg/mL)

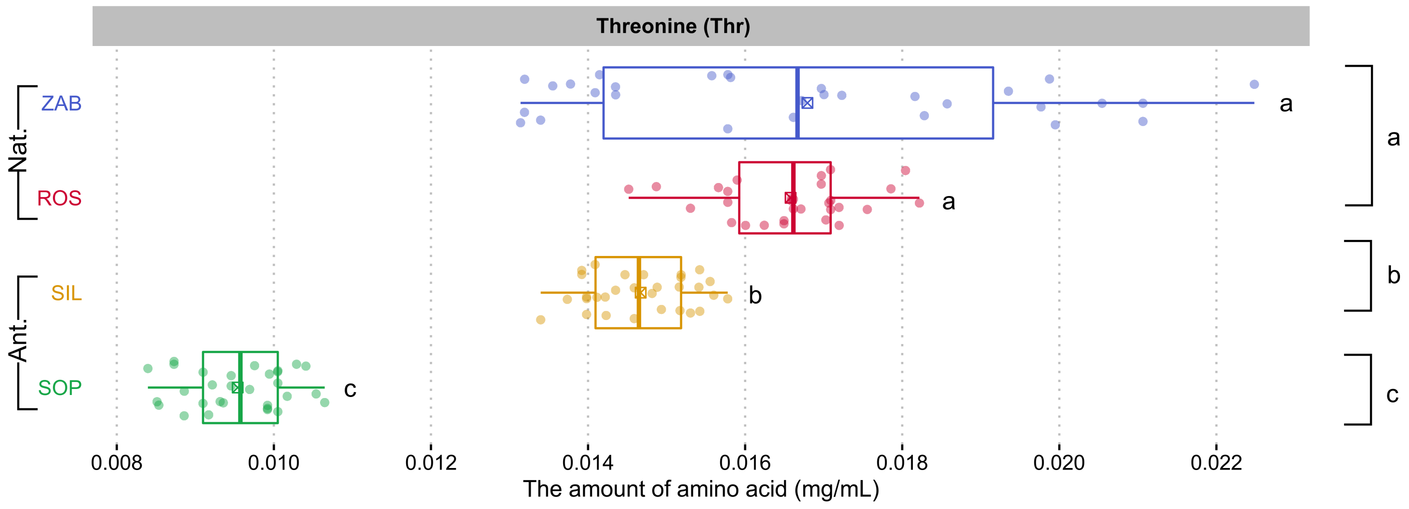
Threonine (Thr)
Nat.
ZAB
ROS
Ant.
SIL
SOP
a
a
b
c
a
b
c
0.008
0.010
0.012
0.014
0.016
0.018
0.020
0.022
The amount of amino acid (mg/mL)

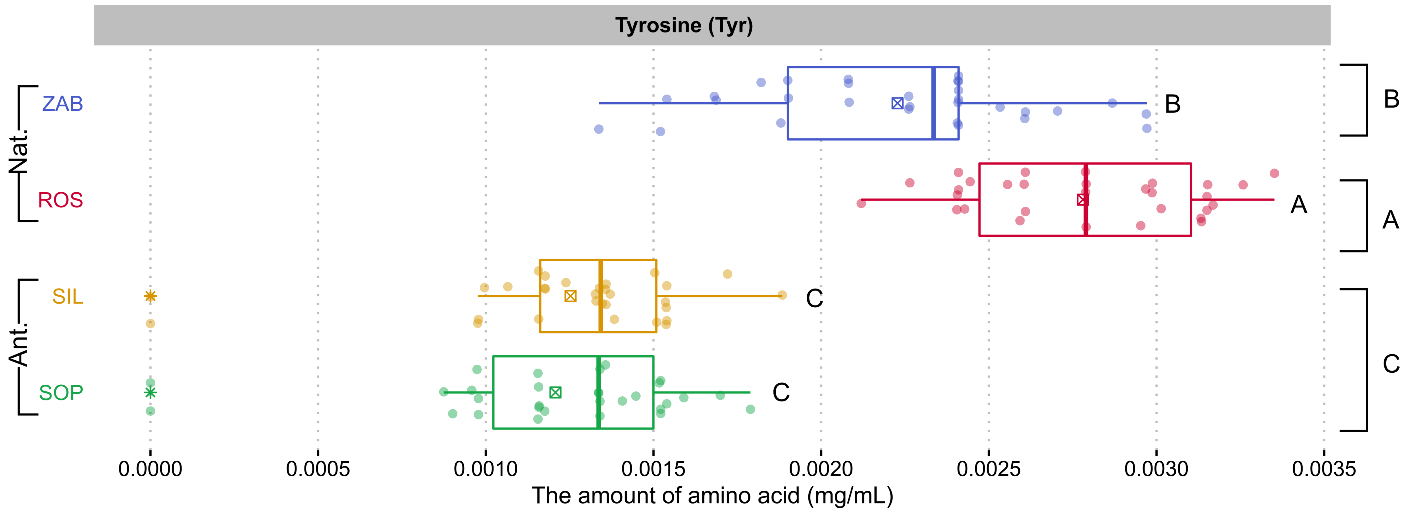
Tyrosine (Tyr)
Nat.
ZAB
ROS
Ant.
SIL
SOP
B
A
C
C
B
A
C
0.0000
0.0005
0.0010
0.0015
0.0020
0.0025
0.0030
0.0035
The amount of amino acid (mg/mL)

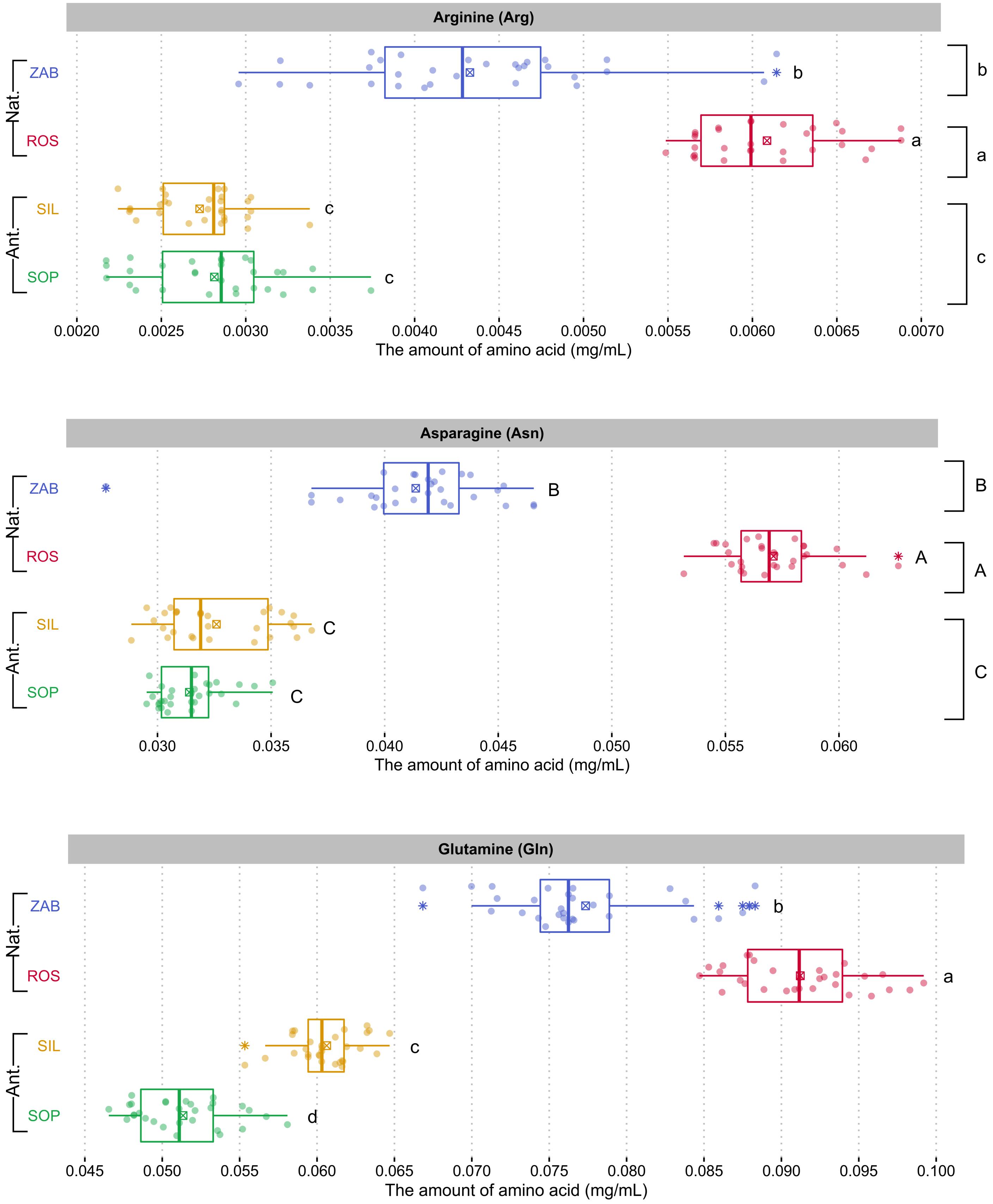
Arginine (Arg)
Nat.
ZAB
ROS
Ant.
SIL
SOP
b
a
c
c
b
a
c
0.0020
0.0025
0.0030
0.0035
0.0040
0.0045
0.0050
0.0055
0.0060
0.0065
0.0070
The amount of amino acid (mg/mL)
Asparagine (Asn)
Nat.
ZAB
ROS
Ant.
SIL
SOP
B
A
C
C
B
A
C
0.030
0.035
0.040
0.045
0.050
0.055
0.060
The amount of amino acid (mg/mL)
Glutamine (Gln)
Nat.
ZAB
ROS
Ant.
SIL
SOP
b
a
c
d
0.045
0.050
0.055
0.060
0.065
0.070
0.075
0.080
0.085
0.090
0.095
0.100
The amount of amino acid (mg/mL)

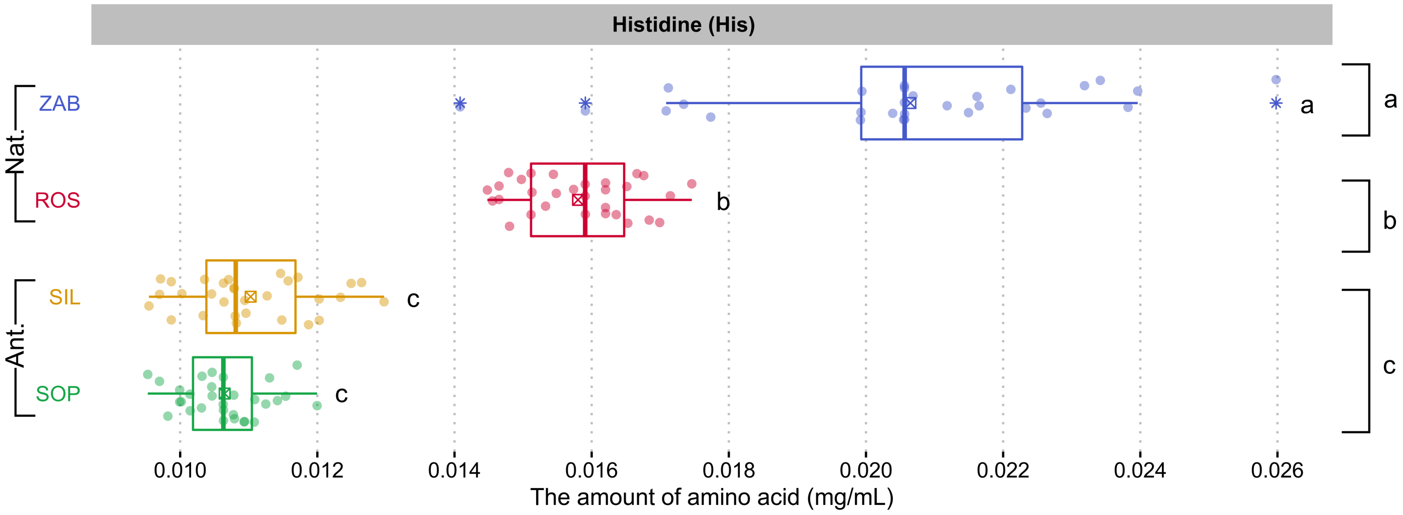
Histidine (His)
Nat.
ZAB
ROS
Ant.
SIL
SOP
a
b
c
c
a
b
c
0.010
0.012
0.014
0.016
0.018
0.020
0.022
0.024
0.026
The amount of amino acid (mg/mL)

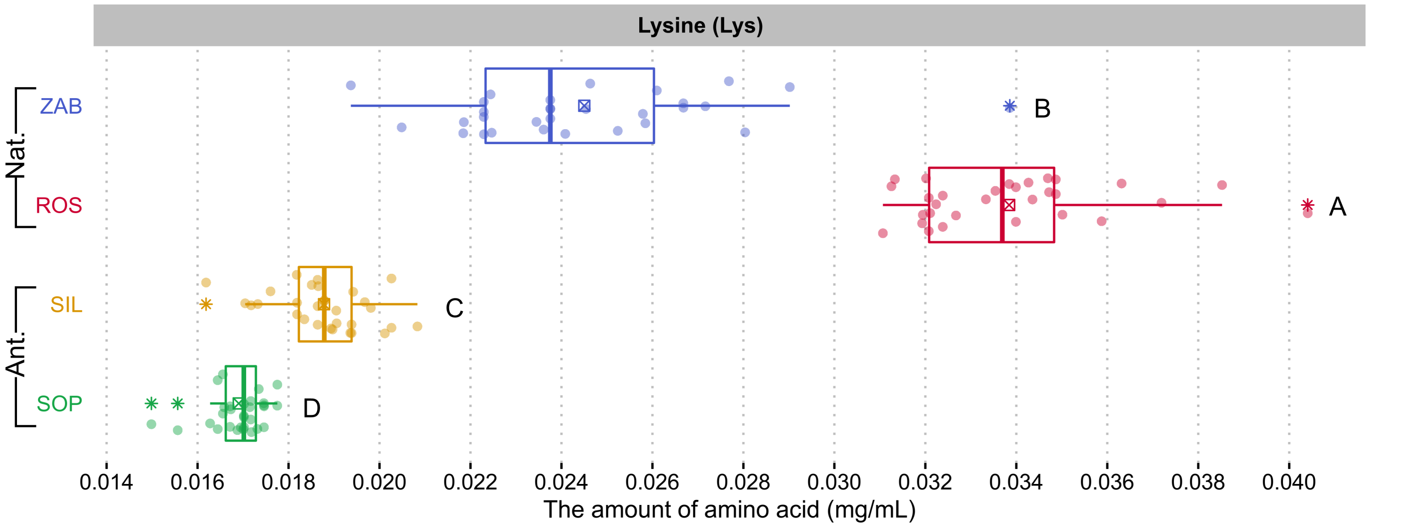
Lysine (Lys)
Nat.
ZAB
ROS
Ant.
SIL
SOP
B
A
C
D
0.014
0.016
0.018
0.020
0.022
0.024
0.026
0.028
0.030
0.032
0.034
0.036
0.038
0.040
The amount of amino acid (mg/mL)

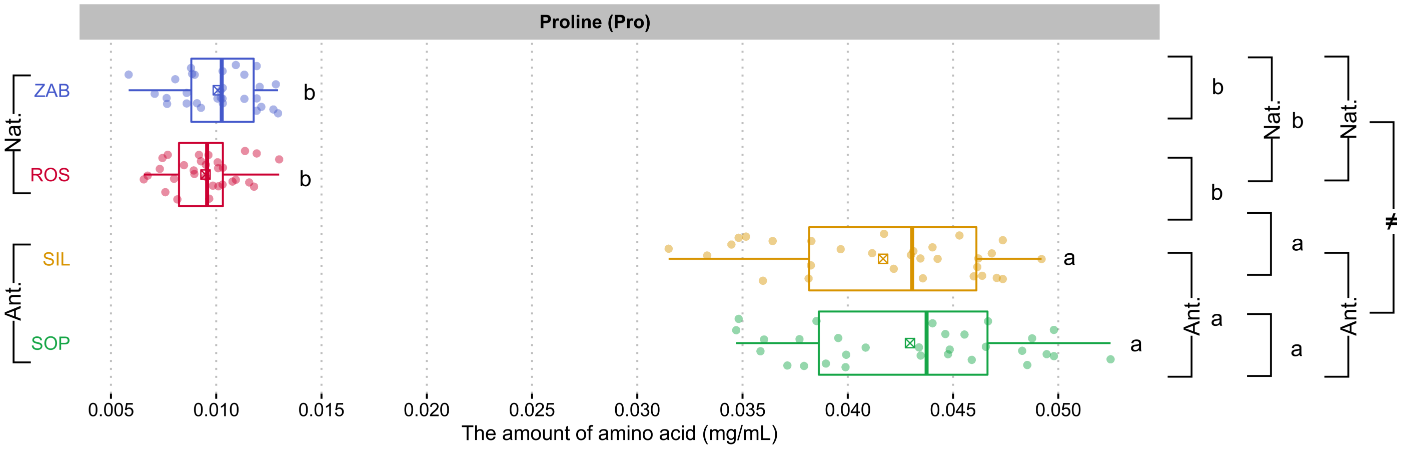
Proline (Pro)
Nat.
ZAB
ROS
Ant.
SIL
SOP
b
b
a
a
≠
0.005
0.010
0.015
0.020
0.025
0.030
0.035
0.040
0.045
0.050
The amount of amino acid (mg/mL)

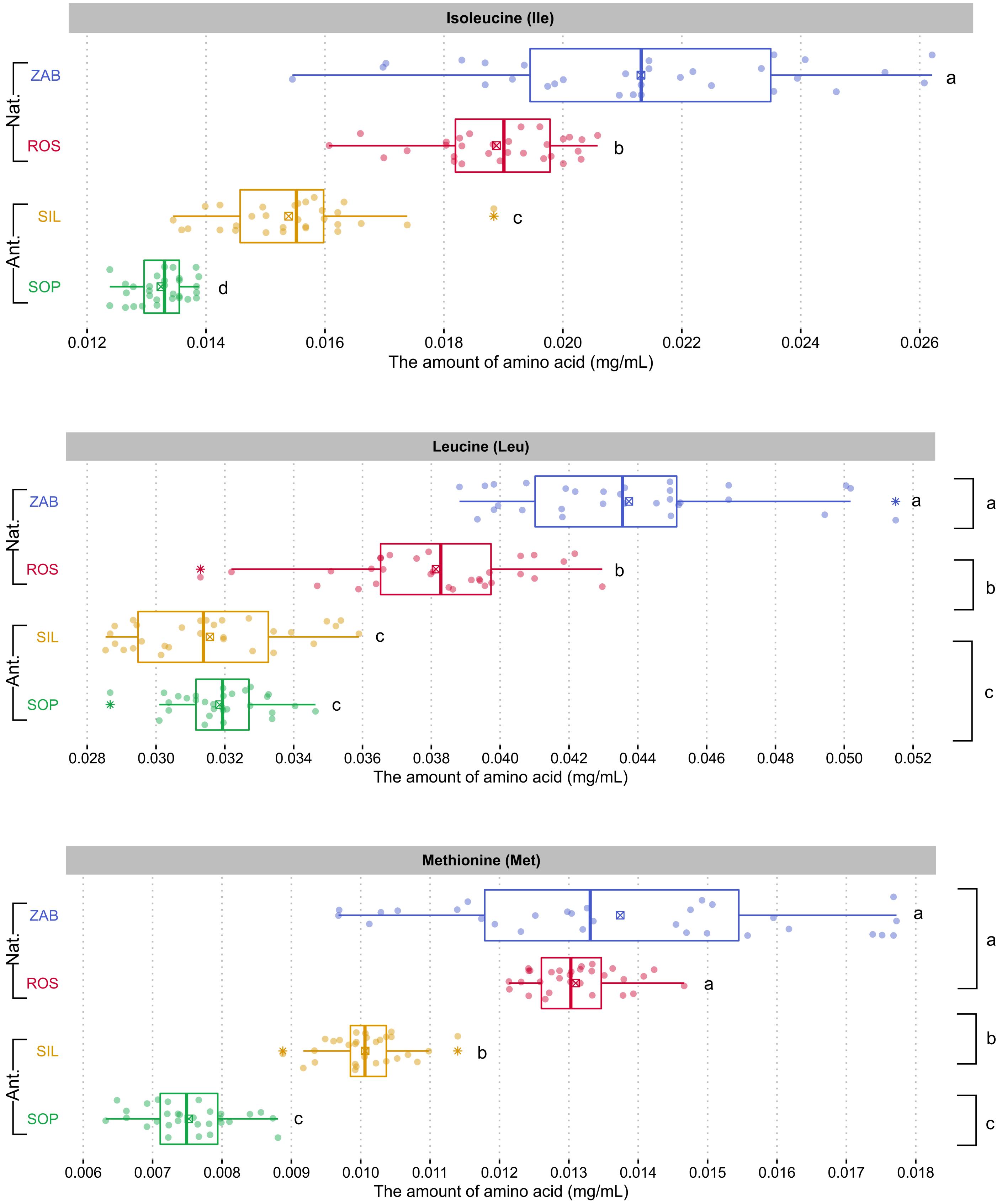
Isoleucine (Ile)
Nat.
ZAB
ROS
Ant.
SIL
SOP
a
b
c
d
0.012
0.014
0.016
0.018
0.020
0.022
0.024
0.026
The amount of amino acid (mg/mL)
Leucine (Leu)
Nat.
ZAB
ROS
Ant.
SIL
SOP
a
b
c
c
0.028
0.030
0.032
0.034
0.036
0.038
0.040
0.042
0.044
0.046
0.048
0.050
0.052
The amount of amino acid (mg/mL)
Methionine (Met)
Nat.
ZAB
ROS
Ant.
SIL
SOP
a
a
b
c
0.006
0.007
0.008
0.009
0.010
0.011
0.012
0.013
0.014
0.015
0.016
0.017
0.018
The amount of amino acid (mg/mL)

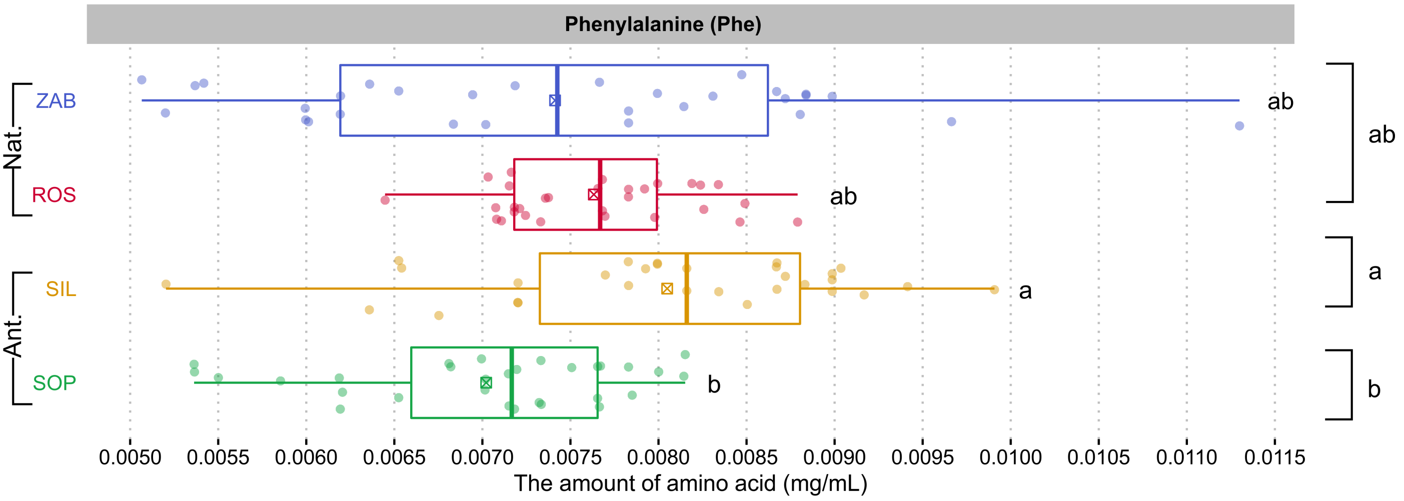
Phenylalanine (Phe)
ZAB
ROS
SIL
SOP
Nat.
Ant.
ab
ab
a
b
ab
a
b
0.0050 0.0055 0.0060 0.0065 0.0070 0.0075 0.0080 0.0085 0.0090 0.0095 0.0100 0.0105 0.0110 0.0115
The amount of amino acid (mg/mL)

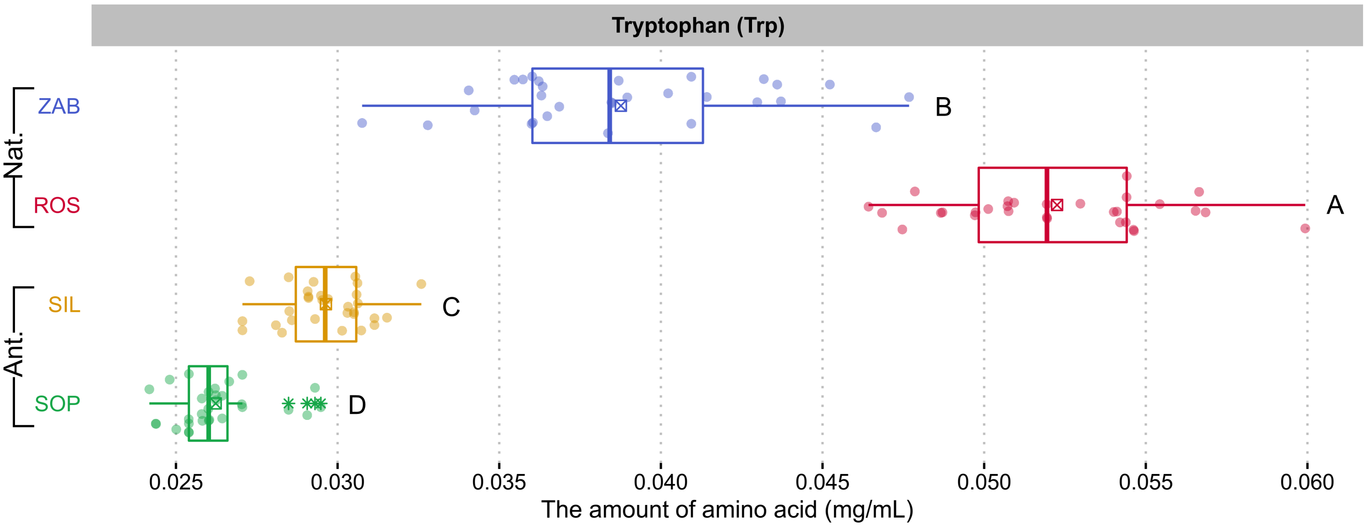
Tryptophan (Trp)
ZAB
ROS
SIL
SOP
Nat.
Ant.
B
A
C
D
0.025 0.030 0.035 0.040 0.045 0.050 0.055 0.060
The amount of amino acid (mg/mL)

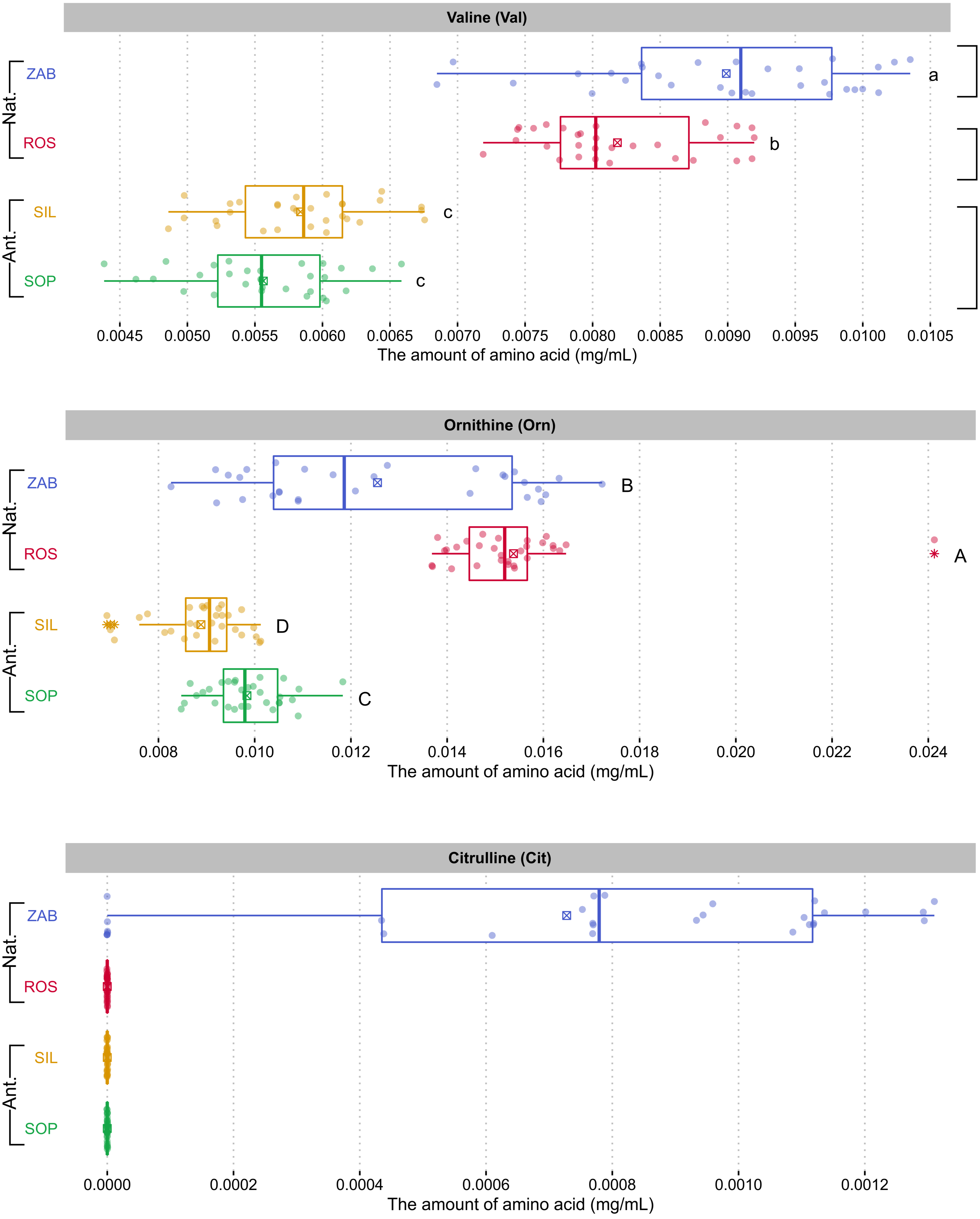
Valine (Val)
Nat.
ZAB
ROS
Ant.
SIL
SOP
a
b
c
c
a
b
c
0.0045
0.0050
0.0055
0.0060
0.0065
0.0070
0.0075
0.0080
0.0085
0.0090
0.0095
0.0100
0.0105
The amount of amino acid (mg/mL)
Ornithine (Orn)
ZAB
ROS
SIL
SOP
B
A
D
C
0.008
0.010
0.012
0.014
0.016
0.018
0.020
0.022
0.024
The amount of amino acid (mg/mL)
Citrulline (Cit)
ZAB
ROS
SIL
SOP
0.0000
0.0002
0.0004
0.0006
0.0008
0.0010
0.0012
The amount of amino acid (mg/mL)

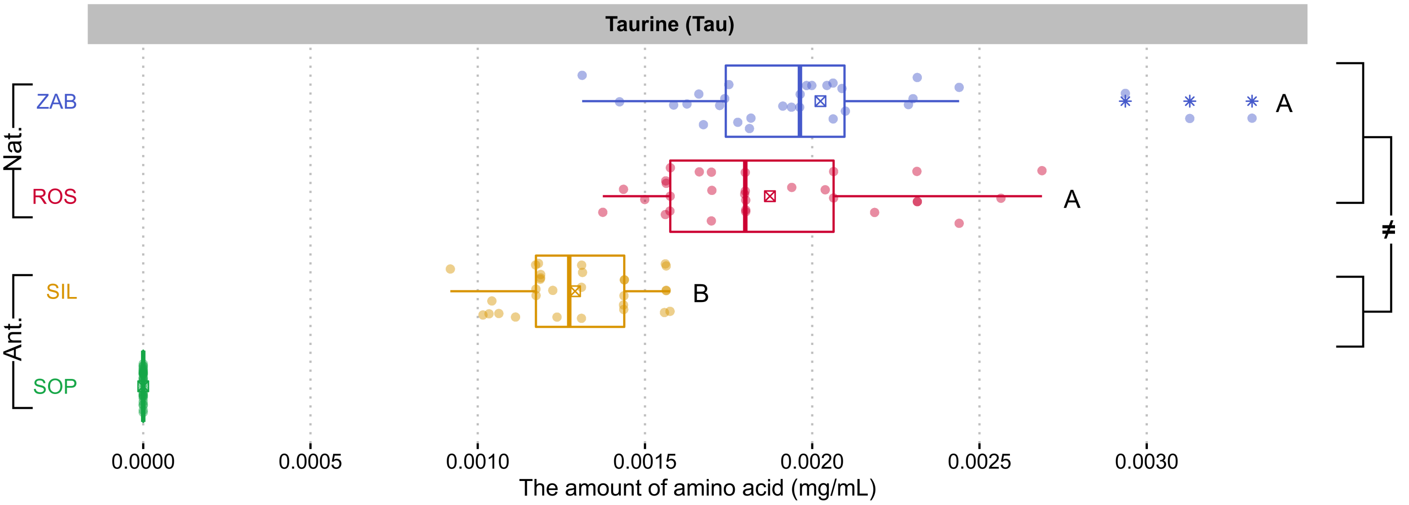
Taurine (Tau)
ZAB
ROS
SIL
SOP
Nat.
Ant.
A
A
B
≠
0.0000
0.0005
0.0010
0.0015
0.0020
0.0025
0.0030
The amount of amino acid (mg/mL)

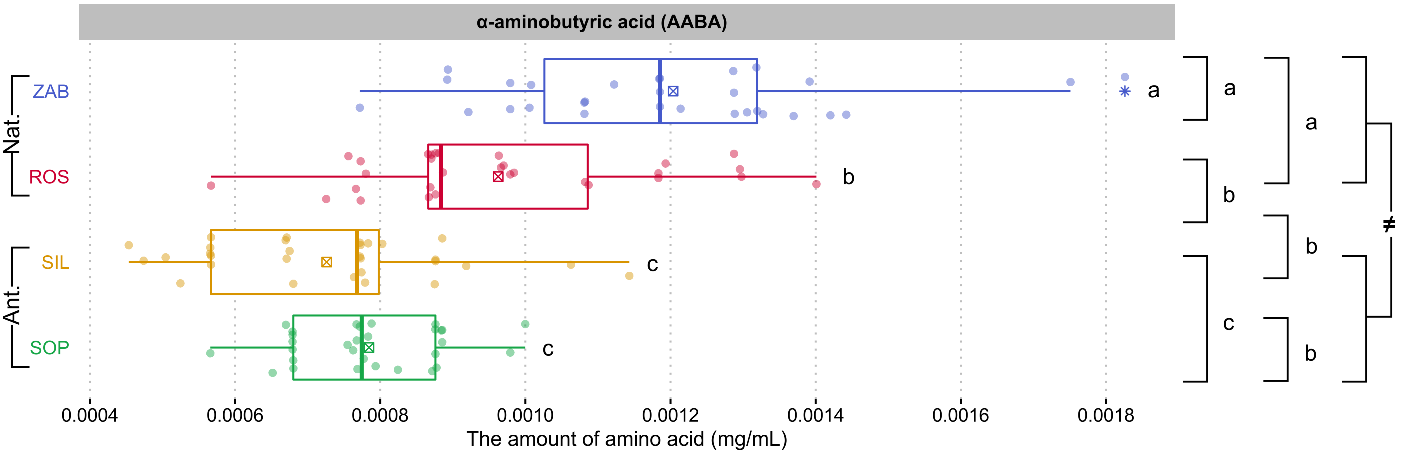
α-aminobutyric acid (AABA)
ZAB
ROS
SIL
SOP
Nat.
Ant.
a
b
c
c
a
b
c
a
b
b
a
≠
0.0004
0.0006
0.0008
0.0010
0.0012
0.0014
0.0016
0.0018
The amount of amino acid (mg/mL)

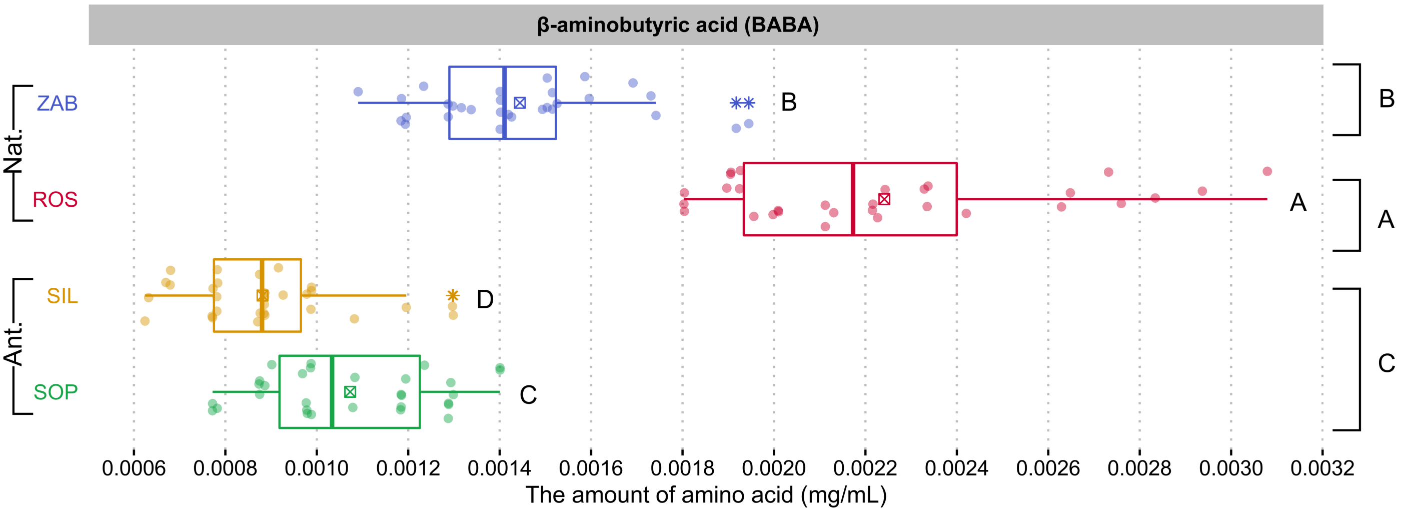
β-aminobutyric acid (BABA)
ZAB
ROS
SIL
SOP
Nat.
Ant.
B
A
D
C
B
A
C
0.0006
0.0008
0.0010
0.0012
0.0014
0.0016
0.0018
0.0020
0.0022
0.0024
0.0026
0.0028
0.0030
0.0032
The amount of amino acid (mg/mL)

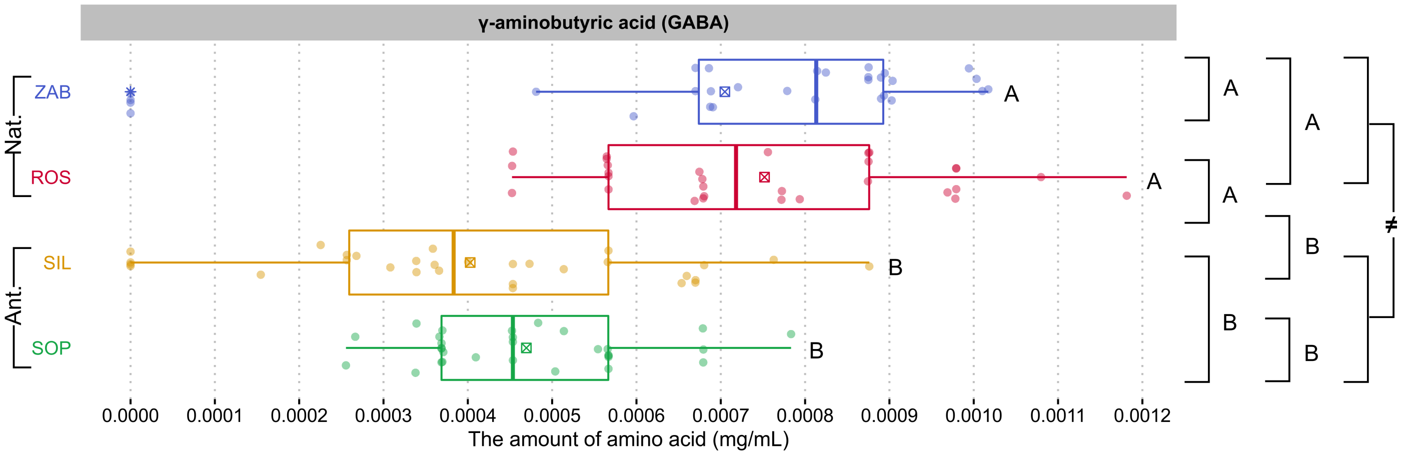
γ-aminobutyric acid (GABA)
Nat.
ZAB
ROS
Ant.
SIL
SOP
A
A
B
B
A
A
B
A
A
B
B
B
≠
0.0000
0.0001
0.0002
0.0003
0.0004
0.0005
0.0006
0.0007
0.0008
0.0009
0.0010
0.0011
0.0012
The amount of amino acid (mg/mL)

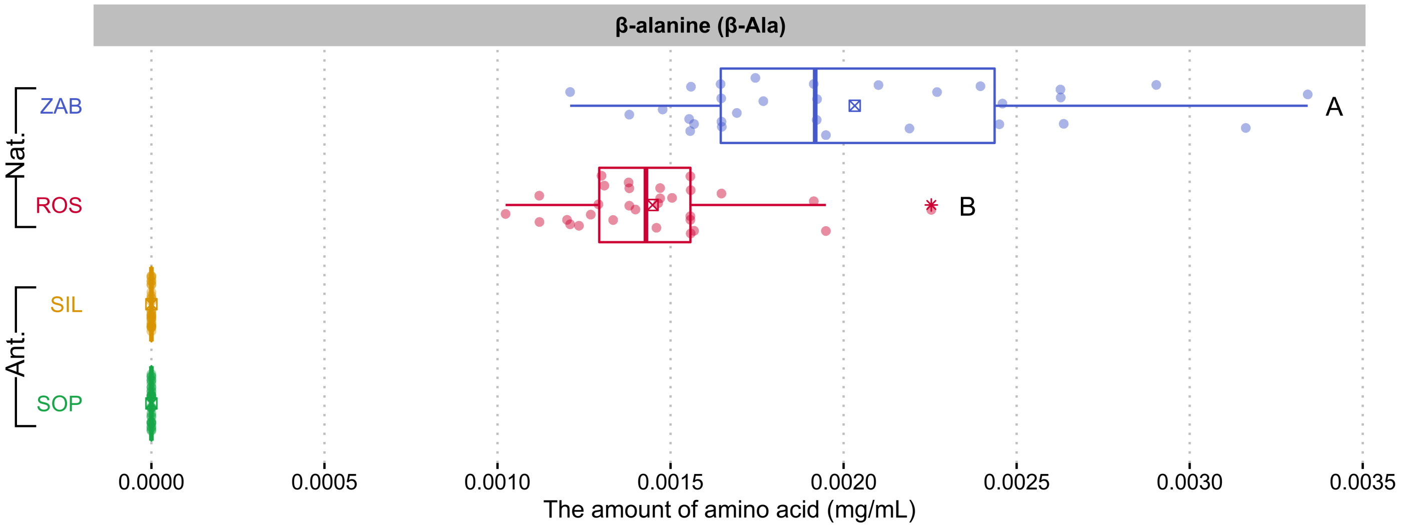
β-alanine (β-Ala)
Nat.
ZAB
ROS
Ant.
SIL
SOP
A
B
0.0000
0.0005
0.0010
0.0015
0.0020
0.0025
0.0030
0.0035
The amount of amino acid (mg/mL)

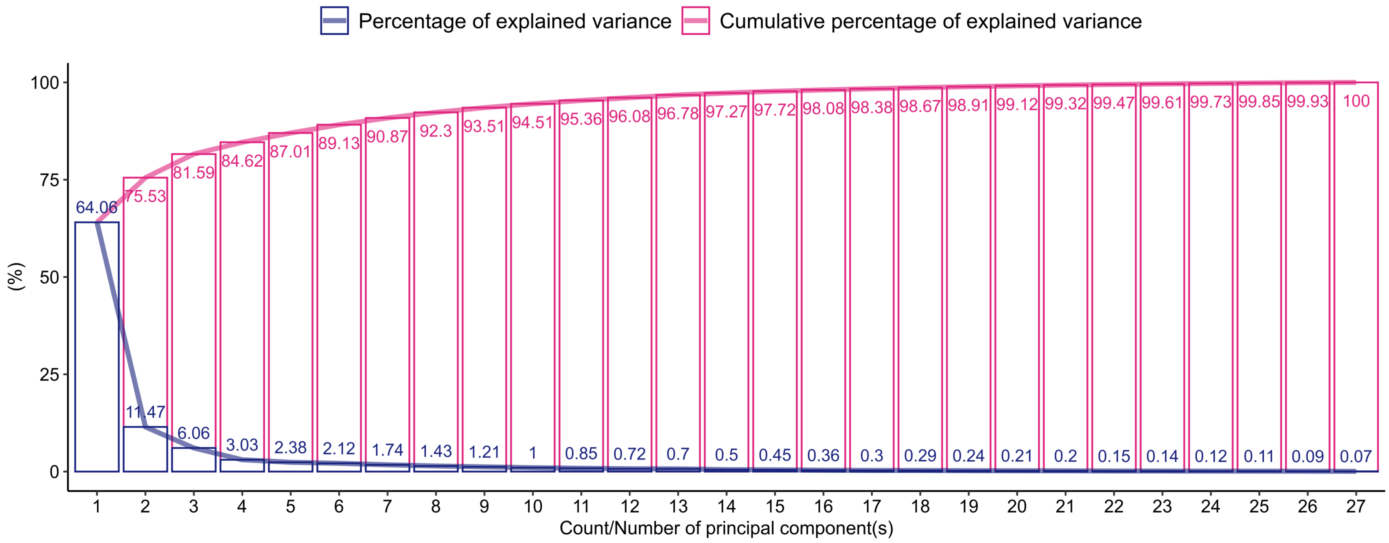

Figure S5. Scree plot showing the proportion of explained variance by the principal components of amino acids PCA model.

	Asp	Glu	Ala	Cys	Gly	Ser	Thr	Tyr	Arg	Asn	Gln	His	Lys	Pro	Ile	Leu	Met	Phe	Trp	Val	Orn	Cit	Tau	AABA	BABA	GABA	betaAla
Dim.1	0.726	0.868	0.407	0.713	0.464	0.863	0.578	0.77	0.814	0.794	0.906	0.597	0.775	0.908	0.667	0.585	0.712	0.007	0.845	0.763	0.629	0.123	0.647	0.333	0.667	0.366	0.767
Dim.2	0.007	0.012	0.188	0.1	0.176	0.039	0.019	0.015	0.087	0.141	0.027	0.279	0.13	0.017	0.158	0.258	0.057	0.008	0.077	0.116	0.038	0.604	0.034	0.238	0.126	0.009	0.138
Dim.3	0.121	0.058	0.054	0.064	0.099	0.025	0.209	0.007	0.013	0.006	0.005	0.001	0.002	0.002	0.011	0.009	0.09	0.516	0	0.005	0.028	0.001	0.171	0.043	0.029	0.065	0.001
Dim.4	0.004	0.001	0.121	0.005	0.052	0.008	0.021	0	0	0	0	0.006	0.014	0.002	0	0	0.001	0.373	0	0.001	0.016	0.001	0.041	0.002	0.014	0.133	0.003
Dim.5	0.017	0.014	0	0.01	0	0.001	0.046	0.007	0	0.004	0	0.02	0.007	0.015	0.003	0	0.002	0.039	0	0.003	0.001	0.028	0.025	0.004	0.004	0.373	0.019
Dim.6	0.01	0.004	0	0.022	0.049	0	0.004	0.001	0.002	0.001	0.013	0.018	0.008	0	0.036	0.014	0.001	0.035	0.002	0.013	0.104	0.009	0	0.219	0.002	0	0.006
Dim.7	0	0.003	0.042	0	0	0.001	0.001	0.009	0	0.001	0.003	0.006	0.003	0	0.032	0.024	0.012	0	0.002	0.004	0.028	0.141	0.001	0.084	0.031	0.036	0.003
Dim.8	0.005	0.006	0.117	0.006	0.006	0.005	0.035	0	0	0.002	0	0.002	0	0	0.022	0.036	0.011	0.014	0.001	0	0.039	0.05	0.002	0.012	0.012	0.004	0
Dim.9	0.016	0.001	0	0.004	0.056	0.003	0.012	0.079	0.011	0.013	0.006	0.007	0.003	0.002	0.006	0.007	0.013	0	0	0.001	0.031	0.001	0.002	0.018	0.03	0.002	0.002
Dim.10	0.015	0.005	0.046	0.009	0.005	0.003	0.006	0.005	0.003	0.003	0	0.003	0	0	0.011	0.017	0.027	0	0.018	0.002	0.052	0.001	0.02	0.014	0.007	0	0
Dim.11	0.015	0	0.013	0	0.066	0.001	0	0.005	0.003	0	0.003	0.001	0.011	0	0.002	0.001	0.022	0.004	0.001	0.009	0	0.004	0.02	0.026	0.022	0	0.001
Dim.12	0.007	0	0	0.028	0	0	0.043	0.011	0.016	0.007	0.001	0.001	0.002	0.006	0.004	0.001	0.008	0	0.007	0.001	0.002	0.017	0.001	0.006	0.013	0.001	0.008
Dim.13	0.008	0.003	0.003	0	0.016	0.005	0.002	0.064	0	0	0.002	0.001	0.008	0.002	0.009	0	0.012	0	0	0.019	0.021	0.004	0.002	0	0.003	0.003	0
Dim.14	0.001	0.001	0.004	0.006	0.003	0	0	0.009	0.009	0.001	0.005	0.021	0.003	0.009	0.009	0.013	0	0	0.001	0	0.001	0.012	0	0	0.014	0.006	0.005
Dim.15	0.031	0.001	0	0.006	0.001	0	0.001	0.013	0.001	0.001	0.003	0	0	0	0	0.001	0.003	0	0.003	0.035	0	0.001	0.006	0	0.011	0.001	0.001
Dim.16	0	0	0	0.001	0.002	0.003	0.013	0	0	0.007	0	0.021	0.002	0.002	0.003	0	0	0	0.003	0.001	0.007	0.001	0.014	0	0.001	0	0.013
Dim.17	0.001	0	0	0.003	0	0.001	0.001	0	0.006	0.001	0	0	0	0.015	0.001	0.002	0.018	0.001	0.008	0.013	0.001	0.001	0	0.001	0	0.001	0.006
Dim.18	0.002	0	0	0.005	0	0.001	0.001	0.001	0	0.001	0	0	0.007	0	0.011	0.017	0.001	0.001	0.014	0	0.001	0	0	0	0	0	0.013
Dim.19	0.006	0	0	0	0.001	0.02	0	0	0.006	0	0.001	0.003	0.002	0	0.003	0.005	0	0	0.003	0.009	0	0	0	0	0	0	0.002
Dim.20	0.004	0	0.002	0	0	0.005	0	0	0.015	0.001	0.001	0	0.001	0.002	0	0.001	0.004	0.001	0.001	0	0	0	0.005	0	0.011	0.001	0
Dim.21	0.002	0	0	0.001	0.001	0.002	0.002	0	0.008	0	0.006	0.003	0.007	0	0.001	0.004	0.003	0	0.001	0.005	0	0	0.002	0	0	0	0.007
Dim.22	0	0.002	0	0.005	0	0.004	0.001	0.001	0	0	0.002	0	0.002	0	0.007	0.003	0	0	0.006	0	0	0	0.003	0	0	0	0.003
Dim.23	0	0	0	0.007	0	0.006	0.002	0	0	0.004	0.002	0.005	0.003	0	0.003	0	0.001	0	0.001	0	0	0.001	0	0	0.002	0	0
Dim.24	0.002	0.017	0	0	0	0	0	0	0.001	0.001	0	0.001	0.007	0	0	0	0.001	0	0	0	0	0	0	0	0	0	0
Dim.25	0	0.001	0	0.001	0	0	0.001	0	0.002	0.005	0.002	0.001	0	0.011	0	0	0	0	0.001	0	0.001	0	0.002	0	0.001	0	0.001
Dim.26	0	0	0	0	0	0.002	0	0	0	0	0.012	0.001	0	0.003	0	0	0	0	0.003	0	0	0	0.002	0	0	0	0.001
Dim.27	0	0.004	0	0.001	0	0	0	0	0	0.006	0	0.001	0.002	0.001	0	0	0	0	0.001	0	0	0	0	0	0	0	0.002

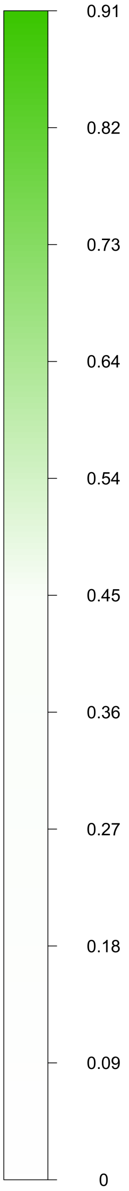

Figure S6. Cos² for the amino acids selected as active variables in the principal component analysis model, representing the quality of representation for variables on the factor map (Dim1-27).

Table S1. *P*-values for different tests on floral display and flower structure datasets (n = 30). M1 – Two-way ANOVA with Tukey's post-hoc test. M2 – Kruskall-Wallis rank sum test with Wilcoxon rank sum test with Benjamini-Hochberg *p*-value correction. Blank cell for *p*-value > 0.05; * for *p*-value ≤ 0.05; ** for *p*-value ≤ 0.01; *** for *p*-value ≤ 0.001. The symbol (-) indicates that the statistical test or M1/M2 analysis could not be performed.

Parameter	Shapiro-Wilk test				Bartlett's test	F test	Welch's F test	Kruskal-Wallis test	Analysis
	ZAB	ROS	SIL	SOP					
Length of dorsal sepal (LDS)					**	***	***	***	M1
Width of dorsal sepal (WDS)					***	***	***	***	M1
Length of petal (LP)					***	***	***	***	M1
Width of petal (WP)					***	***	***	***	M1
Length of lateral sepal (LLS)				*	**	-	-	***	M2
Width of lateral sepal (WLS)				**	***	-	-	***	M2
Width of flowers (FW)	**				***	-	-	***	M2
Length of flowers (FH)					***	***	***	***	M1
Length of labellum (LL)		**	***		*	-	-	**	M2
Width of hypochile (HW)						***	***	***	M1
Length of hypochile (HL)	***	**	**		***	-	-	***	M2
Length of epichile (LE)			**			-	-	***	M2
Length of isthmus (LI)					*	***	***	***	M1
Width of epichile (WE)					*	**	**	*	M1
Width of isthmus (WI)	*			**	**	-	-	***	M2
Isthmus area (AI)	*				***	-	-	***	M1
Shoot height					***	***	***	***	M1
Inflorescence length					***	***	**	***	M1
Flower number	**		***		**	-	-	**	M2

Table S2. Correlation matrices for floral display and flower structure acids dataset. The values show the Spearman's rank correlation coefficient r_s. Significant correlations ($p < 0.05$) are in red. 'x' indicates that correlation analysis could not be performed.

	ZAB population																		
**Floral display and flower structure**	**LDS**	**WDS**	**LP**	**WP**	**LLS**	**WLS**	**FW**	**FH**	**LL**	**HW**	**HL**	**LE**	**LI**	**WE**	**WI**	**AI**	**Shoot height**	**Inflorescence length**	**Flower number**
**LDS**	1.00																		
**WDS**	0.47	1.00																	
**LP**	0.78	0.28	1.00																
**WP**	0.09	0.21	0.24	1.00															
**LLS**	0.80	0.44	0.66	0.07	1.00														
**WLS**	0.47	0.43	0.32	0.40	0.39	1.00													
**FW**	0.70	0.46	0.52	-0.04	0.67	0.27	1.00												
**FH**	0.96	0.48	0.78	0.13	0.71	0.49	0.64	1.00											
**LL**	0.66	0.43	0.53	0.38	0.58	0.56	0.52	0.58	1.00										
**HW**	0.52	0.18	0.36	0.18	0.55	0.25	0.43	0.41	0.58	1.00									
**HL**	0.20	-0.13	0.13	0.26	0.07	0.20	0.12	0.20	0.38	0.27	1.00								
**LE**	0.45	0.55	0.36	0.29	0.45	0.32	0.41	0.38	0.68	0.41	0.05	1.00							
**LI**	0.45	0.05	0.46	0.26	0.22	0.42	0.24	0.51	0.40	0.16	0.44	0.02	1.00						
**WE**	0.06	0.16	0.05	0.25	-0.14	0.14	0.15	0.06	0.29	0.18	-0.09	0.19	-0.05	1.00					
**WI**	0.12	0.25	0.10	0.13	0.07	0.25	0.02	0.10	0.14	0.23	0.05	0.19	0.30	-0.04	1.00				
**AI**	0.38	0.17	0.37	0.28	0.17	0.47	0.17	0.42	0.36	0.22	0.31	0.13	0.85	-0.02	0.71	1.00			
**Shoot height**	0.12	0.52	0.00	0.15	-0.02	0.33	0.12	0.14	0.41	-0.05	0.14	0.34	0.16	0.18	0.03	0.15	1.00		
**Inflorescence length**	0.18	0.40	0.05	0.24	0.02	0.30	0.18	0.17	0.46	0.07	0.21	0.45	0.30	0.36	0.18	0.28	0.74	1.00	
**Flower number**	-0.06	0.39	0.08	0.25	-0.26	0.25	0.05	0.01	0.28	-0.17	-0.15	0.34	0.21	0.29	0.17	0.29	0.49	0.42	1.00

Floral display and flower structure	ROS population																		
	LDS	WDS	LP	WP	LLS	WLS	FW	FH	LL	HW	HL	LE	LI	WE	WI	AI	Shoot height	Inflorescence length	Flower number
LDS	1.00																		
WDS	0.57	1.00																	
LP	0.70	0.60	1.00																
WP	0.59	0.47	0.55	1.00															
LLS	0.74	0.52	0.68	0.51	1.00														
WLS	0.46	0.45	0.64	0.60	0.64	1.00													
FW	0.70	0.44	0.64	0.41	0.67	0.45	1.00												
FH	0.92	0.63	0.79	0.69	0.82	0.58	0.75	1.00											
LL	0.52	0.64	0.48	0.36	0.50	0.23	0.38	0.54	1.00										
HW	-0.03	0.12	0.22	0.14	0.14	0.01	0.16	0.09	0.37	1.00									
HL	0.12	0.51	0.32	0.18	0.25	0.24	0.09	0.21	0.69	0.35	1.00								
LE	0.34	0.57	0.28	0.18	0.44	0.12	0.26	0.38	0.75	0.34	0.35	1.00							
LI	-0.22	-0.06	-0.07	-0.16	0.02	-0.08	0.07	-0.20	0.30	0.58	0.38	0.13	1.00						
WE	0.31	0.47	0.19	0.26	0.31	0.05	0.21	0.31	0.67	0.40	0.37	0.61	0.16	1.00					
WI	0.03	0.24	-0.12	0.14	-0.07	-0.07	0.08	0.03	0.32	0.21	0.21	0.39	0.15	0.54	1.00				
AI	-0.14	0.14	-0.12	0.02	0.00	-0.04	0.12	-0.12	0.39	0.52	0.42	0.33	0.78	0.43	0.71	1.00			
Shoot height	0.30	0.04	0.03	0.07	0.30	0.15	0.15	0.23	0.28	-0.08	-0.05	0.26	-0.15	0.28	0.20	0.01	1.00		
Inflorescence length	0.35	-0.02	0.39	0.18	0.47	0.35	0.33	0.44	0.18	0.12	0.11	0.18	0.14	0.00	-0.04	0.05	0.29	1.00	
Flower number	0.31	0.15	0.31	0.11	0.32	0.23	0.24	0.39	0.25	-0.09	0.22	0.19	-0.04	0.16	0.37	0.13	0.17	0.53	1.00

	**SIL population**																		
**Floral display and flower structure**	**LDS**	**WDS**	**LP**	**WP**	**LLS**	**WLS**	**FW**	**FH**	**LL**	**HW**	**HL**	**LE**	**LI**	**WE**	**WI**	**AI**	**Shoot height**	**Inflorescence length**	**Flower number**
**LDS**	1.00																		
**WDS**	0.52	1.00																	
**LP**	0.69	0.47	1.00																
**WP**	0.66	0.52	0.65	1.00															
**LLS**	0.33	0.52	0.20	0.16	1.00														
**WLS**	0.24	0.76	0.29	0.36	0.45	1.00													
**FW**	0.56	0.41	0.59	0.49	0.49	0.37	1.00												
**FH**	0.95	0.51	0.72	0.63	0.38	0.23	0.55	1.00											
**LL**	0.53	0.65	0.55	0.54	0.34	0.44	0.53	0.52	1.00										
**HW**	-0.35	-0.05	0.01	-0.30	-0.05	0.02	0.02	-0.29	-0.01	1.00									
**HL**	0.14	0.43	0.33	0.43	0.18	0.53	0.43	0.19	0.59	0.25	1.00								
**LE**	0.70	0.38	0.51	0.57	0.43	0.21	0.48	0.67	0.58	-0.47	0.09	1.00							
**LI**	0.11	0.22	0.23	0.24	0.19	0.35	0.35	0.11	0.21	0.31	0.33	0.03	1.00						
**WE**	0.66	0.35	0.47	0.45	0.10	0.21	0.37	0.58	0.55	-0.16	0.12	0.62	0.25	1.00					
**WI**	0.16	0.39	0.13	0.12	0.46	0.38	0.21	0.13	0.06	0.16	0.03	0.22	0.41	0.22	1.00				
**AI**	0.12	0.32	0.18	0.16	0.35	0.40	0.29	0.08	0.14	0.27	0.22	0.11	0.84	0.22	0.80	1.00			
**Shoot height**	0.37	0.07	0.46	0.32	0.12	-0.03	0.31	0.35	0.26	0.02	0.04	0.37	0.16	0.40	-0.07	0.00	1.00		
**Inflorescence length**	0.28	-0.02	0.13	0.20	0.25	-0.19	0.14	0.34	0.14	-0.04	0.16	0.32	-0.02	0.13	-0.06	-0.06	0.58	1.00	
**Flower number**	0.15	-0.13	0.23	-0.01	-0.04	-0.14	-0.02	0.23	-0.11	-0.14	-0.10	0.11	-0.24	0.09	-0.36	-0.38	0.48	0.38	1.00

	SOP population																		
**Floral display and flower structure**	**LDS**	**WDS**	**LP**	**WP**	**LLS**	**WLS**	**FW**	**FH**	**LL**	**HW**	**HL**	**LE**	**LI**	**WE**	**WI**	**AI**	**Shoot height**	**Inflorescence length**	**Flower number**
**LDS**	1.00																		
**WDS**	0.86	1.00																	
**LP**	0.74	0.85	1.00																
**WP**	0.69	0.83	0.83	1.00															
**LLS**	0.72	0.82	0.82	0.75	1.00														
**WLS**	0.77	0.83	0.61	0.68	0.65	1.00													
**FW**	0.89	0.90	0.89	0.76	0.89	0.74	1.00												
**FH**	0.90	0.82	0.75	0.70	0.76	0.74	0.89	1.00											
**LL**	0.70	0.69	0.72	0.57	0.53	0.61	0.69	0.66	1.00										
**HW**	0.29	0.25	0.54	0.30	0.32	0.11	0.41	0.31	0.57	1.00									
**HL**	0.67	0.70	0.76	0.68	0.61	0.57	0.71	0.63	0.80	0.55	1.00								
**LE**	0.78	0.69	0.60	0.59	0.67	0.56	0.73	0.72	0.67	0.32	0.57	1.00							
**LI**	0.36	0.32	0.53	0.45	0.26	0.34	0.36	0.29	0.60	0.67	0.76	0.41	1.00						
**WE**	0.65	0.56	0.42	0.48	0.48	0.44	0.59	0.52	0.46	0.27	0.56	0.69	0.49	1.00					
**WI**	0.59	0.62	0.78	0.61	0.57	0.49	0.63	0.62	0.76	0.56	0.69	0.49	0.61	0.30	1.00				
**AI**	0.55	0.54	0.74	0.61	0.48	0.49	0.59	0.54	0.76	0.70	0.81	0.51	0.89	0.50	0.88	1.00			
**Shoot height**	0.48	0.45	0.48	0.53	0.61	0.44	0.56	0.46	0.27	0.27	0.43	0.20	0.19	0.17	0.19	0.23	1.00		
**Inflorescence length**	0.42	0.47	0.47	0.55	0.61	0.43	0.58	0.41	0.23	0.16	0.36	0.41	0.12	0.19	0.24	0.19	0.65	1.00	
**Flower number**	-0.01	-0.05	-0.13	0.03	0.00	-0.08	-0.06	-0.04	-0.28	-0.18	-0.11	-0.17	-0.23	-0.03	-0.17	-0.22	0.40	0.30	1.00

Table S3. Kaiser-Meyer-Olkin test results for flower structure dataset used for principal components analysis sorted in descending order by the measure of sampling adequacy (MSA) (overall MSA = 0.84).

Parameter	MSA
Width of flowers (FW)	0.96
Width of dorsal sepal (WDS)	0.93
Length of flowers (FH)	0.90
Width of hypochile (HW)	0.90
Length of dorsal sepal (LDS)	0.89
Length of petal (LP)	0.89
Width of lateral sepal (WLS)	0.89
Length of labellum (LL)	0.89
Length of hypochile (HL)	0.88
Width of epichile (WE)	0.86
Length of lateral sepal (LLS)	0.85
Length of epichile (LE)	0.85
Width of petal (WP)	0.82
Length of isthmus (LI)	0.68
Width of isthmus (WI)	0.48

Table S4. The sugar ratios in *Epipactis palustris* nectar. Data (n = 30) represent the mean ($\bar{x}$) ± standard error (SE). Different lowercase letters indicate statistically significant differences according to Tukey's post-hoc test ($p < 0.05$). Different uppercase letters indicate statistically significant differences according to the pairwise Wilcoxon Rank Sum test with Benjamini-Hochberg adjustment ($p < 0.05$).

Parameter	Natural populations		Anthropogenic populations	
	ZAB	ROS	SIL	SOP
Fructose / glucose	1.12 ± 0.024 ^{A}	1.07 ± 0.026 ^{A}	1.14 ± 0.040 ^{A}	1.03 ± 0.029 ^{A}
Sucrose / (fructose + glucose)	0.93 ± 0.033 ^{C}	0.86 ± 0.023 ^{A}	0.57 ± 0.017 ^{B}	0.58 ± 0.011 ^{B}
Sugar content in nectar (sum of sugars, mg/mL)	48.09 ± 7.49 ^{A}	40.68 ± 1.47 ^{B}	34.05 ± 1.36 ^{C}	35.00 ± 1.36 ^{C}

Table S5. *P*-values for different tests on sugars' datasets (n = 30). M1 – Two-way ANOVA with Tukey's post-hoc test. Blank cell for p-value > 0.05; * for p-value ≤ 0.05; ** for p-value ≤ 0.01; *** for p-value ≤ 0.001.

Parameter	Shapiro-Wilk test				Bartlett's test	F test	Welch's F test	Analysis
	ZAB	ROS	SIL	SOP				
Glucose					*	*	*	M1
Fructose						**	**	M1
Sucrose					***	***	***	M1

Table S6. *P*-values for different tests on amino acid′ datasets (n = 30). M1 – Two-way ANOVA with Tukey's post-hoc test. M2 – Kruskall-Wallis rank sum test with Wilcoxon rank sum test with Benjamini-Hochberg *p*-value correction. Blank cell for *p*-value > 0.05; * for *p*-value ≤ 0.05; ** for *p*-value ≤ 0.01; *** for *p*-value ≤ 0.001. The symbol (-) indicates that the statistical test or M1/M2 analysis could not be performed (AA not detected for required number of populations).

Parameter	Shapiro-Wilk test				Bartlett's test	F test	Welch's F test	Kruskal-Wallis test	Analysis
	ZAB	ROS	SIL	SOP					
Aspartic acid (Asp)				**		-	-	***	M2
Glutamic acid (Glu)					*	***	***	***	M1
Alanine (Ala)				**	**	-	-	***	M2
Cysteine (Cys)		**			***	-	-	***	M2
Glycine (Gly)			***		***	-	-	***	M2
Serine (Ser)			***		***	-	-	***	M2
Threonine (Thr)					***	***	***	***	M1
Tyrosine (Tyr)			***	***		-	-	***	M2
Arginine (Arg)					***	***	***	***	M1
Asparagine (Asn)	**				***	-	-	***	M2
Glutamine (Gln)					***	***	***	***	M1
Histidine (His)					***	***	***	***	M1
Lysine (Lys)		**		**	***	-	-	***	M2
Proline (Pro)					***	***	***	***	M1
Isoleucine (Ile)					***	***	***	***	M1
Leucine (Leu)					***	***	***	***	M1
Methionine (Met)					***	***	***	***	M1
Phenylalanine (Phe)					***	**	***	***	M1
Tryptophan (Trp)				**	***	-	-	***	M2
Valine (Val)					**	***	***	***	M1
Ornithine (Orn)	**	***			***	-	-	***	M2
Citrulline (Cit)	***	-	-	-	-	-	-	-	-
Taurine (Tau)	**	*		-	***	-	-	***	M2
α-aminobutyric acid (AABA)					**	***	***	***	M1
β-aminobutyric acid (BABA)		*	*		***	-	-	***	M2
γ-aminobutyric acid (GABA)	***				***	-	-	***	M2
β-alanine (β-Ala)	*	*	-	-	***	-	-	-	M2
Sum of amino acids					***	***	***	***	M1

Table S7. Correlation matrices for amino acids dataset. The values show the Spearman's rank correlation coefficient r_s. Significant correlations ($p < 0.05$) are in red. 'x' indicates that correlation analysis could not be performed.

Amino acid	ZAB population																										
	Asp	Glu	Ala	Cys	Gly	Ser	Thr	Tyr	Arg	Asn	Gln	His	Lys	Pro	Ile	Leu	Met	Phe	Trp	Val	Orn	Cit	Tau	AABA	BABA	GABA	ß-Ala
**Asp**	1.00																										
**Glu**	0.38	1.00																									
**Ala**	0.09	-0.09	1.00																								
**Cys**	0.38	0.20	-0.11	1.00																							
**Gly**	-0.11	-0.35	-0.29	-0.15	1.00																						
**Ser**	-0.03	-0.26	0.11	-0.02	0.15	1.00																					
**Thr**	0.39	0.42	0.08	0.10	0.14	0.22	1.00																				
**Tyr**	0.38	0.20	0.39	0.13	-0.20	0.09	0.09	1.00																			
**Arg**	0.41	0.29	0.34	0.00	-0.17	0.11	0.59	0.47	1.00																		
**Asn**	0.18	0.10	0.42	0.11	-0.17	-0.32	-0.09	0.06	0.04	1.00																	
**Gln**	0.06	-0.18	0.57	0.05	-0.40	-0.11	-0.32	0.19	-0.01	0.43	1.00																
**His**	0.23	0.03	0.28	0.19	-0.36	0.04	-0.04	0.11	-0.06	0.43	0.44	1.00															
**Lys**	-0.24	-0.16	0.06	-0.27	-0.15	0.15	-0.34	-0.04	-0.06	0.19	0.08	0.12	1.00														
**Pro**	-0.27	-0.26	-0.29	0.17	-0.15	-0.23	-0.61	-0.10	-0.33	0.06	-0.01	0.04	0.30	1.00													
**Ile**	0.22	0.11	0.21	0.35	-0.13	0.03	-0.22	0.60	0.09	0.23	0.31	0.09	-0.01	0.05	1.00												
**Leu**	0.27	0.04	0.27	0.28	0.03	0.12	-0.08	0.34	0.00	0.26	0.24	0.20	-0.04	-0.04	0.75	1.00											
**Met**	0.38	0.42	-0.11	0.56	0.06	-0.28	0.27	0.05	0.10	-0.06	-0.03	-0.14	-0.64	-0.25	0.33	0.28	1.00										
**Phe**	0.28	0.21	0.12	0.19	0.10	0.16	0.20	0.02	-0.12	-0.03	0.07	0.12	-0.37	-0.45	0.11	0.18	0.47	1.00									
**Trp**	0.33	0.28	0.09	0.36	-0.33	-0.25	0.02	0.12	-0.06	0.29	0.35	0.38	-0.22	0.16	0.14	0.11	0.32	0.28	1.00								
**Val**	0.25	0.32	0.34	0.34	-0.22	-0.26	0.27	0.16	0.26	0.20	0.36	0.05	-0.10	-0.18	0.27	0.27	0.42	-0.08	0.35	1.00							
**Orn**	0.04	0.29	-0.39	-0.12	0.29	0.03	0.25	0.11	0.07	-0.37	-0.78	-0.35	-0.02	0.10	-0.20	-0.24	0.04	-0.06	-0.17	-0.35	1.00						
**Cit**	0.01	0.06	-0.02	0.15	0.17	0.16	0.12	-0.23	-0.22	0.28	-0.22	0.24	0.05	0.22	-0.28	-0.19	-0.10	0.15	0.33	-0.08	0.15	1.00					
**Tau**	0.08	0.31	-0.06	0.02	0.23	0.28	0.31	0.33	0.17	-0.20	-0.60	-0.28	-0.17	-0.18	0.13	0.23	0.14	0.25	-0.21	-0.32	0.55	0.17	1.00				
**AABA**	-0.19	0.23	0.16	-0.17	0.03	-0.10	0.10	0.08	0.24	0.23	-0.26	-0.32	-0.17	-0.03	0.26	0.22	0.14	-0.06	-0.19	-0.04	0.14	-0.01	0.47	1.00			
**BABA**	0.03	0.15	0.43	0.17	-0.20	-0.16	0.04	0.06	0.02	0.49	0.51	0.43	0.14	-0.04	0.18	0.29	0.02	0.21	0.45	0.54	-0.55	0.28	-0.25	-0.02	1.00		
**GABA**	0.29	0.50	0.22	0.21	-0.25	-0.07	0.35	0.33	0.39	0.20	0.09	0.07	-0.21	-0.13	0.42	0.40	0.35	-0.04	0.35	0.47	0.09	-0.21	0.08	0.42	0.17	1.00	
**ß-Ala**	0.42	0.16	0.25	0.30	-0.04	0.20	0.21	0.35	0.15	0.04	0.22	0.33	-0.10	-0.30	0.20	0.34	0.24	0.39	0.40	0.31	-0.18	0.24	0.22	-0.31	0.49	0.03	1.00

	ROS population																										
**Amino acid**	**Asp**	**Glu**	**Ala**	**Cys**	**Gly**	**Ser**	**Thr**	**Tyr**	**Arg**	**Asn**	**Gln**	**His**	**Lys**	**Pro**	**Ile**	**Leu**	**Met**	**Phe**	**Trp**	**Val**	**Orn**	**Cit**	**Tau**	**AABA**	**BABA**	**GABA**	**ß-Ala**
**Asp**	1.00																										
**Glu**	0.55	1.00																									
**Ala**	0.24	0.28	1.00																								
**Cys**	-0.15	0.06	0.07	1.00																							
**Gly**	-0.22	-0.04	-0.17	0.06	1.00																						
**Ser**	0.18	0.18	0.03	-0.29	-0.32	1.00																					
**Thr**	0.17	-0.19	-0.30	-0.16	0.18	-0.02	1.00																				
**Tyr**	0.09	-0.11	0.15	0.00	0.01	0.19	-0.18	1.00																			
**Arg**	0.07	-0.15	-0.17	0.16	-0.11	0.19	0.33	-0.21	1.00																		
**Asn**	-0.32	0.00	-0.07	0.07	-0.30	0.29	-0.36	0.22	-0.05	1.00																	
**Gln**	-0.09	0.09	-0.24	0.05	0.47	-0.20	-0.06	-0.31	-0.14	-0.13	1.00																
**His**	0.06	0.32	-0.06	-0.28	0.16	0.10	0.09	-0.31	-0.03	-0.11	0.01	1.00															
**Lys**	-0.23	-0.06	-0.07	-0.07	-0.16	0.25	-0.38	0.16	-0.24	0.48	0.15	-0.21	1.00														
**Pro**	0.14	0.19	-0.01	0.49	0.07	-0.08	0.14	-0.17	0.34	0.00	-0.02	-0.03	-0.29	1.00													
**Ile**	-0.12	-0.03	-0.56	0.15	0.19	0.07	0.20	-0.34	0.11	-0.07	0.40	0.15	-0.29	0.30	1.00												
**Leu**	0.05	0.15	-0.36	0.20	0.41	-0.19	0.01	-0.21	-0.06	-0.22	0.64	0.06	-0.19	0.16	0.74	1.00											
**Met**	0.15	0.40	-0.06	-0.03	0.26	-0.11	-0.11	0.36	-0.37	0.02	0.16	-0.03	0.11	-0.21	-0.10	0.32	1.00										
**Phe**	-0.20	-0.23	0.10	0.10	0.01	-0.01	0.26	-0.14	0.38	0.00	-0.01	0.22	-0.25	0.34	0.09	-0.03	-0.36	1.00									
**Trp**	0.19	0.00	-0.16	-0.03	0.04	-0.01	0.67	-0.32	0.11	-0.24	0.07	0.16	-0.23	0.02	0.24	-0.05	-0.15	0.01	1.00								
**Val**	-0.01	0.36	0.16	-0.01	0.36	-0.01	-0.03	-0.04	-0.18	-0.19	0.51	0.24	0.02	-0.05	0.06	0.19	0.28	0.23	0.01	1.00							
**Orn**	-0.21	0.17	-0.29	0.06	0.67	0.08	0.10	-0.20	0.07	-0.17	0.60	0.34	-0.02	0.11	0.40	0.54	0.23	0.05	-0.01	0.42	1.00						
**Cit**	x	x	x	x	x	x	x	x	x	x	x	x	x	x	x	x	x	x	x	x	x	1.00					
**Tau**	-0.37	0.06	-0.35	0.16	0.48	-0.13	-0.09	-0.41	0.07	-0.14	0.69	0.02	0.09	0.03	0.33	0.40	0.14	-0.02	0.02	0.41	0.63	x	1.00				
**AABA**	0.60	0.29	0.20	-0.14	0.04	-0.15	0.21	0.04	-0.09	-0.23	0.15	0.28	-0.24	-0.16	-0.06	0.13	0.11	0.00	0.36	0.15	-0.05	x	-0.21	1.00			
**BABA**	-0.26	-0.25	-0.46	-0.18	0.18	0.34	0.22	-0.28	0.32	-0.01	0.32	0.15	0.13	0.19	0.49	0.30	-0.36	0.34	-0.03	-0.02	0.48	x	0.36	-0.32	1.00		
**GABA**	0.05	-0.01	-0.04	-0.02	0.20	-0.17	-0.05	-0.35	0.03	-0.53	0.49	0.03	-0.03	0.03	0.17	0.38	-0.08	0.10	-0.10	0.23	0.30	x	0.47	-0.19	0.41	1.00	
**ß-Ala**	0.03	-0.14	0.26	-0.10	0.09	-0.10	0.14	-0.10	-0.25	-0.38	0.00	-0.11	-0.06	-0.12	-0.10	-0.24	-0.16	-0.06	0.29	0.17	-0.17	x	0.07	0.01	-0.15	0.12	1.00

	SIL population																										
**Amino acid**	**Asp**	**Glu**	**Ala**	**Cys**	**Gly**	**Ser**	**Thr**	**Tyr**	**Arg**	**Asn**	**Gln**	**His**	**Lys**	**Pro**	**Ile**	**Leu**	**Met**	**Phe**	**Trp**	**Val**	**Orn**	**Cit**	**Tau**	**AABA**	**BABA**	**GABA**	**ß-Ala**
**Asp**	1.00																										
**Glu**	0.45	1.00																									
**Ala**	0.10	-0.15	1.00																								
**Cys**	-0.07	-0.08	0.22	1.00																							
**Gly**	-0.06	0.20	0.21	-0.43	1.00																						
**Ser**	0.04	0.46	0.07	-0.29	0.33	1.00																					
**Thr**	-0.01	0.20	0.59	0.13	0.36	0.47	1.00																				
**Tyr**	0.18	0.31	0.17	0.21	0.24	0.17	0.58	1.00																			
**Arg**	0.10	0.12	-0.33	0.04	-0.12	-0.29	-0.13	0.33	1.00																		
**Asn**	0.20	0.30	0.45	-0.23	0.33	0.70	0.46	0.27	-0.26	1.00																	
**Gln**	0.08	0.53	-0.19	0.33	-0.08	0.04	-0.06	0.30	0.30	0.00	1.00																
**His**	0.03	0.33	0.50	0.22	0.24	0.59	0.46	0.20	-0.18	0.66	0.05	1.00															
**Lys**	-0.22	0.00	-0.23	0.16	-0.12	-0.24	-0.20	0.02	-0.09	-0.30	0.14	-0.12	1.00														
**Pro**	-0.06	-0.41	0.42	0.53	-0.26	-0.17	0.17	0.02	0.10	-0.06	0.01	0.17	-0.15	1.00													
**Ile**	0.26	-0.05	0.70	0.01	0.21	0.11	0.24	-0.05	-0.12	0.43	-0.23	0.42	-0.42	0.39	1.00												
**Leu**	0.36	0.06	0.55	0.14	-0.04	-0.16	0.25	-0.03	-0.22	0.07	-0.27	0.07	-0.31	0.08	0.46	1.00											
**Met**	-0.01	0.30	0.19	0.11	0.15	0.12	0.38	0.37	0.16	0.12	-0.03	0.35	-0.22	-0.29	-0.03	0.20	1.00										
**Phe**	-0.19	0.33	-0.02	-0.14	0.46	0.48	0.34	0.21	-0.16	0.40	0.28	0.37	0.12	-0.40	-0.22	-0.25	0.26	1.00									
**Trp**	0.24	-0.16	0.37	0.36	-0.26	-0.37	0.05	0.02	0.00	-0.22	-0.23	-0.06	-0.28	0.38	0.27	0.51	0.11	-0.66	1.00								
**Val**	0.27	-0.02	0.42	0.42	-0.21	-0.13	0.25	0.07	-0.02	-0.02	-0.26	0.21	-0.26	0.47	0.38	0.72	0.20	-0.39	0.60	1.00							
**Orn**	0.25	0.38	0.48	0.37	0.17	0.24	0.54	0.44	-0.10	0.38	0.05	0.67	-0.07	0.13	0.29	0.30	0.49	0.11	0.36	0.43	1.00						
**Cit**	x	x	x	x	x	x	x	x	x	x	x	x	x	x	x	x	x	x	x	x	x	1.00					
**Tau**	-0.26	-0.02	-0.18	0.07	0.02	-0.13	-0.01	0.23	0.11	-0.22	0.06	-0.15	0.45	-0.29	-0.22	-0.35	0.23	0.08	-0.13	-0.42	0.04	x	1.00				
**AABA**	0.55	0.25	0.08	-0.10	0.17	-0.04	0.10	0.09	0.03	-0.01	-0.12	-0.14	-0.38	-0.17	0.14	0.31	0.11	-0.13	0.39	0.14	0.21	x	-0.06	1.00			
**BABA**	0.01	0.06	-0.21	-0.17	0.04	0.31	-0.12	-0.07	-0.07	0.12	0.09	0.16	0.26	-0.28	-0.14	-0.45	-0.12	0.46	-0.58	-0.58	-0.24	x	0.24	-0.23	1.00		
**GABA**	0.08	-0.09	0.41	0.47	0.01	-0.16	0.11	0.12	-0.02	-0.04	-0.06	0.36	0.06	0.21	0.29	0.13	0.35	-0.13	0.44	0.13	0.51	x	0.38	0.10	0.11	1.00	
**ß-Ala**	x	x	x	x	x	x	x	x	x	x	x	x	x	x	x	x	x	x	x	x	x	x	x	x	x	x	1.00

	SOP population																										
**Amino acid**	**Asp**	**Glu**	**Ala**	**Cys**	**Gly**	**Ser**	**Thr**	**Tyr**	**Arg**	**Asn**	**Gln**	**His**	**Lys**	**Pro**	**Ile**	**Leu**	**Met**	**Phe**	**Trp**	**Val**	**Orn**	**Cit**	**Tau**	**AABA**	**BABA**	**GABA**	**ß-Ala**
**Asp**	1.00																										
**Glu**	0.07	1.00																									
**Ala**	0.07	-0.06	1.00																								
**Cys**	0.21	0.63	0.34	1.00																							
**Gly**	0.25	-0.01	-0.15	-0.13	1.00																						
**Ser**	0.07	-0.36	0.42	-0.24	0.31	1.00																					
**Thr**	0.20	0.12	-0.09	0.04	0.31	0.06	1.00																				
**Tyr**	0.02	0.38	0.12	0.50	0.09	-0.09	-0.09	1.00																			
**Arg**	0.16	0.04	0.71	0.30	-0.24	0.43	-0.14	0.13	1.00																		
**Asn**	-0.12	-0.27	0.28	-0.08	-0.21	0.25	0.21	0.02	0.46	1.00																	
**Gln**	0.00	0.10	0.40	0.47	-0.20	-0.16	0.21	0.17	0.17	0.15	1.00																
**His**	-0.12	0.03	0.12	0.14	-0.23	-0.06	-0.59	-0.04	0.31	0.10	0.13	1.00															
**Lys**	-0.12	0.11	0.43	0.20	-0.04	0.37	-0.17	0.23	0.16	-0.01	0.27	0.08	1.00														
**Pro**	-0.23	0.05	-0.38	-0.13	-0.16	-0.26	0.12	-0.06	-0.30	-0.05	-0.25	-0.24	-0.43	1.00													
**Ile**	-0.10	-0.02	0.26	0.17	0.11	0.29	0.04	0.25	0.16	0.45	0.33	0.30	0.33	-0.24	1.00												
**Leu**	-0.07	-0.19	0.27	0.01	-0.12	-0.13	-0.17	0.08	0.29	0.43	0.03	0.37	-0.24	0.09	0.25	1.00											
**Met**	-0.17	0.10	0.42	0.30	-0.55	-0.15	-0.02	0.18	0.38	0.33	0.25	0.01	0.18	0.17	-0.01	0.38	1.00										
**Phe**	-0.01	-0.10	0.48	0.14	-0.43	0.17	-0.11	0.12	0.53	0.26	0.32	0.08	-0.08	0.07	0.05	0.07	0.47	1.00									
**Trp**	-0.22	-0.15	0.38	-0.01	-0.48	-0.13	-0.22	-0.04	0.32	0.37	0.19	0.42	0.00	0.08	0.13	0.61	0.66	0.39	1.00								
**Val**	-0.04	-0.24	0.04	-0.17	-0.31	0.05	-0.34	0.02	0.13	0.36	0.02	0.34	0.01	-0.25	0.31	0.15	0.12	0.33	0.35	1.00							
**Orn**	0.08	0.13	0.26	0.23	0.26	0.51	0.05	0.25	0.04	-0.13	-0.08	-0.08	0.72	-0.25	0.29	-0.28	-0.05	-0.24	-0.26	-0.24	1.00						
**Cit**	x	x	x	x	x	x	x	x	x	x	x	x	x	x	x	x	x	x	x	x	x	1.00					
**Tau**	x	x	x	x	x	x	x	x	x	x	x	x	x	x	x	x	x	x	x	x	x	x	1.00				
**AABA**	0.10	-0.17	-0.09	-0.26	-0.31	-0.14	0.08	-0.12	-0.17	0.17	0.02	-0.25	0.07	0.16	0.00	-0.18	-0.15	0.01	-0.02	-0.02	-0.04	x	x	1.00			
**BABA**	0.22	0.24	0.05	0.01	-0.11	0.18	0.04	-0.20	0.35	0.12	-0.34	0.02	-0.05	0.17	-0.11	-0.09	0.01	0.03	-0.03	0.03	-0.06	x	x	0.07	1.00		
**GABA**	0.12	-0.05	0.03	-0.11	0.12	0.34	0.51	-0.39	0.10	0.00	-0.04	-0.40	-0.16	0.06	-0.25	-0.40	-0.20	0.20	-0.24	-0.18	-0.07	x	x	0.03	0.38	1.00	
**ß-Ala**	x	x	x	x	x	x	x	x	x	x	x	x	x	x	x	x	x	x	x	x	x	x	x	x	x	x	1.00

Table S8. Kaiser-Meyer-Olkin test results for amino acids′ dataset used for principal components analysis sorted in descending order by the measure of sampling adequacy (MSA) (overall MSA = 0.95).

Parameter	MSA
Tyrosine (Tyr)	0.98
Tryptophan (Trp)	0.98
Valine (Val)	0.98
Threonine (Thr)	0.97
Arginine (Arg)	0.97
Asparagine (Asn)	0.97
Leucine (Leu)	0.97
α-aminobutyric acid (AABA)	0.97
Aspartic acid (Asp)	0.96
Serine (Ser)	0.96
Glutamine (Gln)	0.96
Histidine (His)	0.96
Proline (Pro)	0.96
Isoleucine (Ile)	0.96
Methionine (Met)	0.96
β-alanine (β-Ala)	0.96
Cysteine (Cys)	0.95
Taurine (Tau)	0.95
Lysine (Lys)	0.94
Glutamic acid (Glu)	0.93
Alanine (Ala)	0.93
β-aminobutyric acid (BABA)	0.93
Glycine (Gly)	0.92
Ornithine (Orn)	0.92
γ-aminobutyric acid (GABA)	0.92
Citrulline (Cit)	0.89
Phenylalanine (Phe)	0.44
